# Chinese patent medicine combined with calcium channel blockers in the treatment of essential hypertension:a Bayes network meta-analysis and systematic review

**DOI:** 10.3389/fphar.2024.1321405

**Published:** 2024-03-15

**Authors:** Liangyu Cui, Xingfang Liu, Yukun Li, Tianyue Jing, Dasheng Liu, Cong Ren, Tong Yin, Yu Wang, Zhiwei Zhao, Jiaheng Wang, Xuejie Han, Liying Wang

**Affiliations:** ^1^ Institute of Basic Research in Clinical Medicine, China Academy of Chinese Medical Sciences, Beijing, China; ^2^ Research Department, Swiss University of Traditional Chinese Medicine, Bad Zurzach, Switzerland; ^3^ Shaanxi University of Chinese Medicine, Xianyang, China

**Keywords:** ethnopharmacological research, calcium channel Blockers(CCBs), hypertension, network meta-analysis, review studies, therapeutic efficacy

## Abstract

**Backgroud:** The co-administration of Chinese patent medicine with calcium channel blockers (CCBs) is a prevalent practice in China for treating essential hypertension (EH). However, robust evidence supporting the efficacy and safety of tailored combinations of different Chinese patent medicines with CCBs, according to individual patient conditions, is still limited. This study sought to elucidate the efficacy and safety of these combinations using a systematic review and network meta-analysis.

**Materials and methods:** Relevant studies were sourced from established databases, incorporating randomized controlled trials published up to 1 February 2023. The ROB2 tool from the Cochrane Collaborative Network was employed to independently assess and cross-verify the quality of the included literature. A network meta-analysis was conducted in accordance with the Preferred Reporting Items for Systematic Reviews and Meta-Analysis (PRISMA) 2020 and PRISMA-Network Meta-Analyses (PRISMA-NMA) guidelines. A Bayesian network meta-analysis was utilized to gauge the efficacy and safety of distinct integrations of Chinese patent medicine and CCBs. Primary outcomes were interpreted using a paired fixed-effect meta-analysis. Publication bias was appraised through Egger’s test and represented with funnel plots. All statistical analyses were executed within the R statistical framework.

**Results:** Following rigorous selection, data extraction, and bias evaluation, 36 articles were incorporated. Tianma Gouteng Granule, when combined with CCBs, displayed superior efficacy in reducing systolic blood pressure (SBP). In terms of diastolic blood pressure (DBP) reduction, Songling Xuemaikang Capsule combined with CCBs emerged as the most effective. Regarding enhancement of antihypertensive effective rates, Qinggan Jiangya Capsule paired with CCBs demonstrated optimal results. For diminishing Traditional Chinese Medicine syndrome scores, the Qiangli Dingxuan Tablet and CCBs combination proved most beneficial. When aiming to reduce total cholesterol (TC), triglycerides (TG), and low-density lipoprotein cholesterol (LDL-C) levels, Tianma Gouteng Granule and CCBs showcased superior results. In contrast, the combination of Songling Xuemaikang Capsule and CCBs was more effective in reducing LDL-C, tumor necrosis factor-alpha (TNF-α), and interleukin-6 (IL-6).

**Conclusion:** This study underscores variability in outcomes from combining Chinese patent medicine and CCBs for hypertension, emphasizing the importance of personalized medicinal combinations, especially Tianma Gouteng Granule and Songling Xuemaikang Capsule. The results offer robust evidence to inform clinical guidelines for essential hypertention and significantly aid clinician in seleting appropriate Chinese patent medicines for treatment.

## 1 Introduction

Essential hypertension (EH) stands as the leading preventable risk factor for cardiovascular disease (CVD), contributing significantly to conditions such as coronary heart disease, heart failure, stroke, and cognitive impairment (Forouzanfar et al., 2017). The 2017 Global Burden of Disease Study revealed that high systolic blood pressure (SBP) is a primary risk factor, responsible for 10.4 million deaths and impacting 218 million people in terms of disability-adjusted life years (Stanaway, 2017). Among those aged 18 and above, the incidence rate of EH is 27.9%, with a pre-hypertension rate of 39.1% (Forouzanfar et al., 2015). Age plays a crucial role in EH prevalence: it is around 34.38% in middle-aged individuals and surges to nearly 90% in those over 80 years (Beaney et al., 2020), posing a substantial financial and psychological burden on societies and families.

While the primary treatment for EH centers around conventional medications known for their efficacy, ACE inhibitors (ACEI), angiotensin II receptor blockers (ARB), also referred to as sartans, Calcium Channel Blockers (CCBs), and thiazide diuretics are recognized as first-line antihypertensive drugs (James et al., 2014). However, specific challenges like particular contraindications and inadequate control in certain hypertension variants persist. For instance, ACEI and ARB are not advised for pregnant women due to heightened risks linked to renal teratogenicity (Mancia et al., 2013). CCBs target calcium channels situated at the plasma membrane, inducing cell depolarization (Godfraind, 2005). They cause vasodilation by blocking calcium entry, hence diminishing the active tone of vascular smooth muscle. This property has rendered CCBs especially beneficial for EH treatment (Morel and Godfraind, 1987). Nevertheless, despite the availability of such potent antihypertensive drugs, only one in every four women and one in five men with EH attain their treatment objectives (Ncd-RisC, 2021).

Traditional Chinese Medicine (TCM) offers a unique therapeutic perspective, especially with its holistic stance and syndrome differentiation-centric treatments. Leveraging antihypertensive Chinese patent medicine tailored to specific syndromes can efficiently regulate blood pressure and relieve related symptoms. For instance, Ilex hainanensis Merr., also known as Shan-Lv medicine, when paired with antihypertensive drugs, exhibits enhanced efficacy in blood pressure reduction than standalone drug treatments (Yang et al., 2018). Similarly, the Songling Xuemaikang Capsule combined with conventional medicine has demonstrated clear efficacy and safety for EH management (Meng et al., 2022). Over the years, pharmacological management of hypertension has advanced. In the management of EH, combining Chinese patent medicines with western medicines provides superior efficacy and safety compared to conventional treatment alone. Clinical studies have endorsed the blending of traditional Chinese medicine with conventional medicine in treating EH, underscoring mutual benefits, sustained pressure-relief effects, minimized adverse reactions, and fewer cardiovascular complications (Chen et al., 2013). CCBs play an important role in the treatment of EH as CCBs is one of the first recommended antihypertensive drugs. The combination of Chinese patent medicines with CCBs has been extensively utilized as an alternative treatment strategy for essential hypertension (EH) and dizziness in China. This strategy could improve the traditional Chinese medicines symptoms of EH patients, including dizziness, impetuosity, insomnia, tinnitus, etc., which is particularly important for improving the quality of life of EH patients (Lin et al., 2023).

Though numerous hypertension guidelines promote combination pharmacotherapy, solid evidence elucidating the clinical effectiveness and safety of Chinese patent medicine when integrated with CCBs for EH treatment remains scarce. Past meta-analyses have typically compared only two treatment methodologies, failing to provide a comprehensive overview of potential synergies between various Chinese patent medicines and CCBs (Yongcheng et al., 2022; Tong et al., 2023). An extant network meta-analysis did not distinctly categorize the conventional medicines, hindering its clinical application (Zhaochen et al., 2022).

Network meta-analysis (NMA) amalgamates both direct and indirect evidence, offering estimates for every treatment pair (Mavridis, 2019). It serves clinicians by ranking interventions based on their efficacy for each assessed outcome (Nikolakopoulou et al., 2020). This research aims to offer an exhaustive systematic review of RCTs focused on the synergy of Chinese patent medicine and CCBs in EH treatment, strictly adhering to the PRISMA 2020 and PRISMA-NMA guidelines (Hutton et al., 2015; Page et al., 2021). By employing the Bayes network meta-analysis, we aspire to gauge the efficacy and safety of different Chinese patent medicine-CCBs combinations, thereby offering robust evidence to guide clinical decisions.

## 2 Materials and methods

### 2.1 Search strategy

All relevant randomized controlled trials (RCTs) including one or more interventions were identified through extensive searches of databases including PubMed, Cochrane Library, Web of Science, ClinicalTrials.gov, CNKI, WANFANG, VIP, and Sinomed. The timeline for the search spanned from the inception of each database up to 1 February 2023. To ensure a thorough examination of studies related to CCBs, the search began with a broad focus on RCTs that explored the integrative use of Chinese patent medicine with conventional medicine. The subsequent screening refined the results to spotlight studies specifically utilizing CCBs as the antihypertensive treatment. The primary search criteria were defined as: (subject = hypertension OR essential hypertension) AND (subject = Chinese and Western medicine OR Chinese patent medicine OR capsule OR tablet OR scatter OR pill OR ointment OR dan OR dropping pill OR granule OR oral liquid). For a more comprehensive retrieval of relevant studies, synonym expansion was also incorporated into the search formula.

### 2.2 Inclusion and exclusion criteria

We did meta-analysis and bayes network meta-analysis, which means the integration of direct and indirect comparisons, to compare 6 types of Chinese patent medicine for essential hypertension. Our analysis included studies of people with essential hypertension. Because multiple-treatments meta analysis require a reasonably homogeneous sample, we excluded RCTs done in patients with severe cardiovascular, cerebrovascular, and renal diseases; patients with special types of hypertension, such as gestational hypertension, menopausal hypertension, H-type hypertension, etc.

Studies selected were required to be peer-reviewed, published in either Chinese or English, and to employ a RCT design focusing on patients diagnosed with essential hypertension. Interventions assessed combinations of Chinese patent medicine and CCBs, while control groups received only CCBs. The literature we excluded including unable to obtain full text or incomplete information.

### 2.3 Outcome measures

Outcomes were chosen based on the《Guidelines for clinical application of Chinese patent medicine in treating essential hypertension》and subsequently, RCTs were reviewed. Primary outcomes comprised Systolic and Diastolic Blood Pressure (SBP and DBP), antihypertensive effective rate, and adverse drug reaction events. If data from this scale were not available, SBP& DBP or antihypertensive effective rate is essential. Secondary outcomes were Traditional Chinese Medicine Syndrome Score (TCM syndrome score), Total Cholesterol (TC), Triglyceride (TG), Low-Density Lipoprotein Cholesterol (LDL-C), Quality of Life Score, Tumor Necrosis Factor-α (TNF-α), Interleukin-6 (IL-6), and Vascular Endothelin-1 (ET-1). Notably, outcomes such as high-density lipoprotein, cardiac function, and others were excluded owing to insufficient comparative data. Because bayes network meta-analysis requires reasonable homogeneity we focused on 8-week duration, and if this is not available, we used data from between 4 and 12 weeks (closest to 8 weeks).

### 2.4 Data extraction

Data from each study were meticulously extracted, encompassing study details (authorship and year of publication), participant demographics (group classifications, sample size, age), interventions (specific drug names and dosing durations), and a broad spectrum of outcomes including measures like SBP, DBP, TCM syndrome score, and various biochemical markers. Outcomes with continuous data were represented by both pre-intervention and post-intervention means and standard deviations, while dichotomous data were tabulated in 2 × 2 formats. To ensure accuracy and objectivity, two independent reviewers undertook the extraction. In cases of disagreement, resolution was sought with the intervention of a third reviewer. Subsequent to extraction, data were reformatted for compatibility with the R package’s requirements.

### 2.5 Risk of bias assessment

The quality of the selected literature was evaluated using the ROB2 tool from the Cochrane Collaborative Network. Two researchers independently carried out the assessment and cross-checked their evaluations. In cases of disagreement, a third researcher made the decision. The ROB2 tool focuses on assessing potential risks of bias in these areas: bias arising from the randomization process, bias due to deviations from intended interventions, bias due to missing outcome data, bias in measurement of the outcome, and bias in selective reporting of outcomes (Sterne et al., 2019). Bias arising from the randomization process is about whether the allocation sequence was random, adequately concealed, or baseline differences between intervention groups suggest a problem with the randomization process. Bias due to deviations from intended interventions included whether participants, carers and people were aware of participants’assigned intervention during the trial. The purpose of this domain is also to address if applicable deviations from the intended intervention are a result of experimental context in addition to the effect of assignment to intervention. Bias due to missing outcome data is about whether data for this outcome were available for all, or nearly all, participants randomized, (if applicable) the result was not biased by missing outcome data; (if applicable) missingness in the outcome was likely to be influenced by its true value. Bias in measurement of the outcome is about whether the method of measuring the outcome was inappropriate; different intervention groups could used different mesurement or ascertainment for outcome; outcome assessors were aware of the intervention received by study participants; (if applicable) assessment of the outcome was likely to have been influenced by knowledge of intervention received. And at last, bias in selection of the report result addressed whether the trial was analysed according to a pre-specified plan which was finalized before unblinded outcome data were available for analysis; the numerical result being assessed may have been selected, on the basis of the results, from multiple outcome measurements within the outcome domain; the numerical result being assessed is likely to have been selected, on the basis of the results, from multiple analyses of the data. The response options are yes, probably yes, probably no, no, no information. Some signalling questions were logically related each other, i.e., it may be possible to skip next question because an option was selected for a previous signalling question; if a signalling question was skipped due to this logic setting, it was noted as not applicable (NA). If the risk of bias assessment results of all domains were “low risk”, then the overall risk of bias was “Low” risk. If the risk of bias evaluation results of some areas were “Some concers” risk and there was no area of “High” risk, then the overall risk of bias was “Some concers” risk. Whenever there was an area where the risk of bias was assessed as “High” risk, the overall risk of bias is “High” risk. Up-to-date information from the developers on RoB 2 and more detail is available via the Risk of Bias tools website: www.riskofbias.info and Cochrane Scientific Committee.

For each source of bias, studies were categorized as having a high, low, or some concerns risk based on, and summarise the answers to signalling questions (Higgins and Page, 2019).

### 2.6 Statistical analysis

This study conducted a NMA according to the Preferred Reporting Items for Systematic Reviews and Meta-Analysis (PRISMA) 2020 and PRISMA-Network Meta-Analyses (PRISMA-NMA) guidelines (Hutton et al., 2015; Page et al., 2021). Bayes network meta-analysis was used to evaluate the efficacy and safety of various Chinese patent medicine combined with CCBs, aiming to inform clinical decisions.

The initial phase entailed the design of multiple network geometries to decipher the comparative dynamics between different medications, each integrated with distinct Chinese patent medicines. Subsequently, a consistency analysis juxtaposed direct and indirect therapeutic effect estimates. Given the absence of closed-loop networks in this research, only indirect comparative estimates apply. Anticipating study heterogeneity, we deploy a random-effect model.

We focused on constructing a Bayes model for an NMA concerning primary and secondary outcomes. We guided the model by four Markove chains on R software to perform NMA. When calculating the effect size, dichotomous data was expressed as odds ratio (OR), continuous variables were expressed as mean difference (MD), bayes network meta-analysis set 95% credible interval (CrI). Using *I* ^*2*^ to test heterogeneity, and *p* < 0.05 was considered statistically significant.

Gelman-Rubin-Brooks plots were conducted to examine the convergence in diagnostic model combining interval-based graphical evaluation and quantitative analysis of PSRF. After *n* iterations, the curve was formed and observed whether the curve is fit with each other and kept stable. A Potential Scale Reduction Factor (PSRF) tended to 1.00 indicating that the degree of convergence was satisfactory (Jiahao et al., 2021), otherwise increase the number of iterations to achieve model convergence. When calculating the rank probability intervention the parameter preferred direction was equal to 1 means a higher value which indicates a better result in the antihypertensive effective rate and the quality of life score. Other outcomes took preferred direction equaling to −1 to indicate a lower value which means better results.

Trace plot was used to test whether the Markov chain reach stable and overlap during the caluation proces. It always showed the fluctuation process of Markove chain during the iterative computation process, which can be expressed in different forms depending on the number of iterations and preset distributions (Dodds and Vicini, 2004; Toft et al., 2007).

We conducted density plots to evaluate the consistent. Density plots were based on a predefined distribution, and after numerical simulation, the distribution of the *a posteriori* values is observed to see if it is consistent with the predefined distribution. The bandwidth value be used as a quantitative assessment. And the smaller the bandwidth, the smaller difference between distribution range of the parameter *a posteriori* values and the preset distribution range. After enough iterations, the Bandwidth tends to 0 and stabilizes (Harpole et al., 2014).

Then the efficacy of the interventions was compared, and then they were ranked. The Surface Under the Cumulative Ranking (SUCRA) score, visualized through a heatmap, offered a perspective on the probable efficacy of an intervention based on the ranking of all interventions. A SUCRA of x% meant that the drug achieves x% of the effectiveness of this imaginary drug, thus larger SUCRAs denoted more eff ective interventions (Leucht et al., 2013).

We conducted sensitivity analyses on the primary outcome to explore potential reasons for heterogeneity or inconsistency. Leave-one-out sensitivity analysis was used by metafor package in R software to perform a “leave-one-out” function, which examined the robustness of the results by repeatedly fitting the specified model, omitting one study at a time. The size of the forest plot squares was plotted in proportion to the sample size of each RCT to see which RCT carried more weight.

Subgroup analyses were used to examine whether there are significant differences in the effectiveness of interventions between different subgroups to explore which interventions are more effective. Contour-enhanced funnel plot was drawn to detect publication bias due to the suppression of non-significant findings (Peters et al., 2008). Plot of influence diagnostics was drawn to compute outlier and influential case diagnostics, which include externally standardized residuals, DFFITS values, Cook’s distances, covariance ratios, leave-one-out estimates of the amount of heterogeneity, leave-one-out values of the test statistics for heterogeneity, hat values, and weights (Viechtbauer and Cheung, 2010).

The network meta-analyses were facilitated by the netmeta and gemtc packages (Gert van Valkenhoef, 2021; Guido Schwarzer, 2023). All statistical evaluations are conducted in the R statistical environment (R 4.2.2, www.r-project.org).

## 3 Results

### 3.1 Literature search process

**FIGURE 1:**
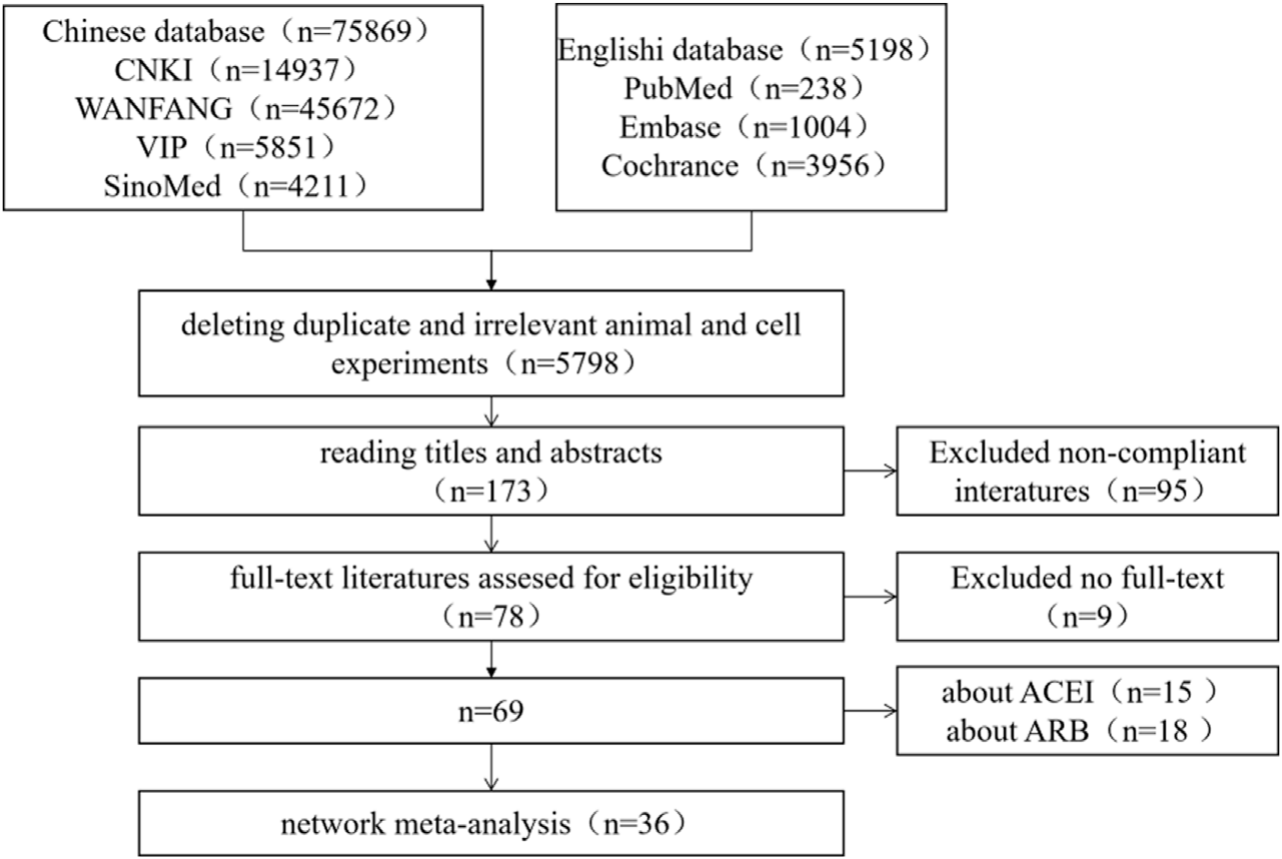
Prisma flow diagram for literature screening.

An initial search of the literature database yielded 75,869 documents. After removing duplicates in Endnote, Prisma flow diagramand helped to identify RCTs for the remaining 70,756 records. 64,958 records were rejected as animal and cell experiment studies, and 5,798 papers were left. Upon reviewing the titles and abstracts, 173 articles that matched the study criteria were shortlisted. A thorough reading of the full text and further screening based on the inclusion criteria left 78 relevant Chinese papers. Studies with fewer than 2 outcomes were excluded, leaving 69 papers. Out of these, 36 articles focusing on the combination of Chinese patent medicine with CCBs were ultimately selected for this study. The detailed screening process is illustrated in Figure 1.

### 3.2 Included literature quality evaluation

A total of 3,740 patients participated in studies involving six distinct types of Chinese patent medicine. These medicines include: Songling Xuemaikang Capsule (SLXM), Qiangli Dingxuan Tablet (QLDX), Tianma Gouteng Granule (TMGT), Qiju Dihuang Pill (QJDH), Qinggan Jiangya Capsule (QGJY), and Xinmaitong Capsule (XMT). The essential characteristics of the studies under consideration are detailed in Table 1. Exact information about Chinese patent medicines in our studies can be found in Supplementary Material S1. All the plant names have been checked with http://www. worldfloraonline.org mentioning on 25 May 2023.

**TABLE 1:** Characteristics of included studies.

Study	n		Age		Interventions		T	Outcome
					C:Western medicine			
					E:Chinese patent medicine			
					Combine with western mecine			
	C	E	C	E	CPM	WM		
Yu (2019)	47	47	51.19 ± 6.92	50.42 ± 7.23	SLXM	Amlodipine Besylate	4W	①②④⑩
Sun (2015)	58	58	61.5 ± 8.2	61.5 ± 8.2	SLXM	Nifedipine	8W	①②③
Liu et al. (2020)	50	50	49.06 ± 8.77	51.52 ± 10.20	SLXM	Benidipine Hydrochloride	4W	①②④⑤⑥⑦⑨⑬
Li (2017)	49	49	56.39 ± 6.71	56.42 ± 6.70	SLXM	Levoamlodipine Maleate Dispersible Tablet	8W	①②③⑤⑥⑨⑧
Wang (2011)	70	68	52.1 ± 9.3	50.3 ± 8.9	SLXM	Amlodipine Besylate Tablet	4W	①②⑩
Qiu et al. (2021)	50	50	69.01 ± 4.17	68.74 ± 4.25	SLXM	Nicardipine Hydrochloride Tablet	4W	①②③⑪⑫
Xin and Lin (2016)	50	50	—	—	SLXM	Amlodipine Besylate Tablet	12W	①②
Gao and Jin (2013)	60	60	66.31 ± 10.75	65.24 ± 11.32	SLXM	Nifedipine	2W	①②③
Zhang et al. (2014a)	60	60	55.1 ± 15.2	54.2 ± 14.9	SLXM	L-levamlodipine Besylate Tablet	8W	⑤⑥⑦⑨
Zhang et al. (2014b)	81	81	56.2 ± 13.9	54.8 ± 14.6	SLXM	L-levamlodipine Besylate Tablet	8W	①②③⑧
He et al. (2013)	29	30	62.74 ± 3.67	62.42 ± 3.82	SLXM	Nifedipine	4W	①②
Liu 2013)	48	48	43–70	46–67	QLDX	NifedipineSustained-release Tablet	12W	①②③
Xiong (2018)	63	63	59.0 ± 3.1	61.0 ± 3.1	QLDX	NifedipineSustained-release Tablet	12W	①②③⑧
Chen and Qiu (2018)	40	40	50–80	50–80	QLDX	Lacidipine Tablet	6W	③
Huang et al. (2018)	39	39	55.9 ± 5.20	55.1 ± 5.67	QLDX	Lacidipine Tablet	6W	①②③④
Jia and Liu (2004)	34	32	56 ± 10	57 ± 9	TMGT	Levamlodipine Besylate Tablet	4W	③
Ye et al. (2021)	60	60	61.19 ± 5.36	7.21 ± 1.87	TMGT	Levoamlodipine Maleate	8W	①②③⑩⑬
Zhu et al. (2015)	54	53	55.8 ± 5.4	56.3 ± 7.2	TMGT	Nifedipine	12W	③
Zhou (2013)	48	49	65.4 ± 9.5	64.9 ± 10.1	TMGT	Nifedipine	12W	①②③④⑧
Sun (2019)	75	75	69.29 ± 1.31	69.19 ± 2.21	TMGT	Nifedipine	8W	①②③⑧
Hao et al. (2020)	30	30	—	—	TMGT	Felovipine	4W	③⑨
Wang (2020)	55	55	53.81 ± 8.12	52.48 ± 7.29	TMGT	NifedipineSustained-release Tablet	8W	③⑧⑬
Yuan (2017)	52	52	76.28 ± 3.56	76.89 ± 3.71	TMGT	Nifedipine Sustained-release Tablet	12W	①②③⑪⑫⑬
Huang and Fu (2014)	51	48	28.16 ± 7.34	30.94 ± 6.23	TMGT	L-levamlodipine Besylate Tablet	4W	③⑤⑥⑦
Wang (2019)	57	57	74.2 ± 6.9	72.5 ± 7.1	TMGT	Nifedipine	12W	③⑧⑪⑫⑬
Hang et al. (2018)	47	47	57.89 ± 2.35	57.67 ± 2.42	TMGT	L-levamlodipine Besylate Tablet	8W	①②③
Zhang (2014)	34	34	48.56 ± 6.32	46.78 ± 5.71	QJDH	Amlodipine Besylate Tablet	4W	①②③
Du et al. (2019)	30	30	51.2 ± 6.6	51.5 ± 6.9	QJDH	NifedipineSustained-release Tablet	4W	①②③
Jiao et al. (2018)	40	40	64.7 ± 7.2	65.2 ± 6.5	QJDH	Amlodipine Besylate	4W	①②
Du et al. (2009)	60	60	64.63 ± 8.01	63.37 ± 8.16	QJDH	Felodipine Sustained Release Tablet	4W	①②⑬
Zhao (2012)	40	40	65–80	65–80	QGJY	NifedipineSustained-release Tablet	4W	①②⑧
Yan (2017)	100	100	66.2 ± 11.4	65.9 ± 12.4	QGJY	NifedipineSustained-release Tablet	12W	①②③
Wang et al. (2022)	70	70	70.97 ± 7.85	70.56 ± 8.13	XMT	Benidipine Hydrochloride	8W	①②④⑧⑬
Liu (2021)	64	64	53.48 ± 7.12	54.36 ± 7.86	XMT	Verapamil	4W	①②③⑧
Han (2014)	45	45	55	55	XMT	Amlodipine Besylate Tablet	8W	③
Liu et al. (2019)	33	33	68.9 ± 4.2	69.4 ± 4.5	XMT	L-levamlodipine Besylate Tablet	12W	①②③⑤⑥

*Notes*: E: experimental group, C: control group; T:treatment time; W:week; D:day; SLXM:Soling Xuemaikang Capsule; QLDX:Qiangli Dingxuan Tablet; TMGT:Tianma Gouteng Granule; QJDH:Qiju Dihuang Pill; QGJY:Qinggan Jiangya Capsule; XMT:Xinmaitong Capsule.①systolic blood pressure (SBP); ②diastolic blood pressure (DBP); ③antihypertensive effective rate; ④TCM, syndrome score; ⑤total cholesterol (TC); ⑥triglyceride (TG); ⑦low-density lipoprotein cholesterol (LDL-C); ⑧adverse drug reaction events; ⑨quality of life score; ⑩blood pressure variability; ⑪tumor necrosis factor-α (TNF-α); ⑫interleukin-6 (IL-6); ⑬vascular endothelin-1 (ET-1).

### 3.3 Risk of bias assessment of included studies

Domain 1 assessed the bias stemming from the randomization process. Out of 36 articles, 10 (Gao and Jin, 2013; Zhou, 2013; Li, 2017; Yuan, 2017; Yu, 2019; Liu et al., 2020; Wang, 2020; Liu, 2021; Ye et al., 2021) used the term “random” to describe their stratified block randomization method without more details. Furthermore, one study (Hao et al., 2020) employed a stratified random method. Both these approaches were categorized as having a “Low” risk of bias. 8 (Jia and Liu, 2004; He et al., 2013; Han, 2014; Huang and Fu, 2014; Sun, 2015; Xin and Lin, 2016; Sun, 2019; Qiu et al., 2021) had “High” risk because of the bias arsing from the randomization process. Other 17 literature were “Some concerns” risk.

The assessment result of domain 2 showed that 17 (Du et al., 2009; Zhao, 2012; Gao and Jin, 2013; Liu, 2013; Zhou, 2013; Zhang et al., 2014a; Zhang, 2014; Hang et al., 2018; Huang et al., 2018; Jiao et al., 2018; Xiong, 2018; Liu et al., 2019; Wang, 2019; Yu, 2019; Hao et al., 2020; Ye et al., 2021; Wang et al., 2022) were “Low” risk, the other 17 literature (Jia and Liu, 2004; Wang, 2011; He et al., 2013; Han, 2014; Huang and Fu, 2014; Sun, 2015; Zhu et al., 2015; Li, 2017; Yan, 2017; Yuan, 2017; Chen and Qiu, 2018; Sun, 2019; Du et al., 2019; Liu et al., 2020; Wang, 2020; Liu, 2021; Qiu et al., 2021) were “Some concerns” risk, and the rest were “High” risk.

Assessments for the domain “bias due to missing outcome data” differed according to the integrity and missingness of the data, and the result showed that 33 (Du et al., 2009; Wang, 2011; Zhao, 2012; Gao and Jin, 2013; He et al., 2013; Liu, 2013; Zhou, 2013; Zhang et al., 2014a; Zhang et al., 2014b; Han, 2014; Huang and Fu, 2014; Zhang, 2014; Sun, 2015; Zhu et al., 2015; Xin and Lin, 2016; Li, 2017; Yan, 2017; Chen and Qiu, 2018; Hang et al., 2018; Huang et al., 2018; Jiao et al., 2018; Xiong, 2018; Du et al., 2019; Liu et al., 2019; Wang, 2019; Yu, 2019; Hao et al., 2020; Liu et al., 2020; Wang, 2020; Liu, 2021; Qiu et al., 2021; Ye et al., 2021; Wang et al., 2022)were “Low” risk, 3 (Jia and Liu, 2004; Yuan, 2017; Sun, 2019) were “Some concerns” risk.

The assignment to the measurement of the outcome showed 23 (Du et al., 2009; Wang, 2011; Zhao, 2012; Gao and Jin, 2013; Zhou, 2013; Zhang et al., 2014a; Han, 2014; Zhu et al., 2015; Li, 2017; Yan, 2017; Hang et al., 2018; Huang et al., 2018; Xiong, 2018; Du et al., 2019; Liu et al., 2019; Wang, 2019; Yu, 2019; Hao et al., 2020; Liu et al., 2020; Wang, 2020; Liu, 2021; Ye et al., 2021) with “Low” risk, 6 (Liu, 2013; Zhang et al., 2014a; Xin and Lin, 2016; Yuan, 2017; Chen and Qiu, 2018; Jiao et al., 2018) with “Some concerns” risk and 7 (Jia and Liu, 2004; He et al., 2013; Huang and Fu, 2014; Zhang, 2014; Sun, 2015; Sun, 2019; Qiu et al., 2021)with “High” risk.

Domain 5 was about the bias in selection of the reported result with 5 (Yan, 2017; Hao et al., 2020; Wang, 2020; Liu, 2021; Ye et al., 2021) “Low”, 29 (Jia and Liu, 2004; Du et al., 2009; Wang, 2011; Zhao, 2012; Gao and Jin, 2013; Liu, 2013; Zhou, 2013; Zhang et al., 2014a; Zhang et al., 2014b; Han, 2014; Huang and Fu, 2014; Sun, 2015; Zhu et al., 2015; Xin and Lin, 2016; Li, 2017; Yuan, 2017; Chen and Qiu, 2018; Hang et al., 2018; Huang et al., 2018; Jiao et al., 2018; Xiong, 2018; Du et al., 2019; Liu et al., 2019; Wang, 2019)"Some concerns” and 2 (He et al., 2013; Zhang, 2014) “High” risk. At last, overall bias with 10 (Gao and Jin, 2013; Zhou, 2013; Li, 2017; Yu, 2019; Hao et al., 2020; Liu et al., 2020; Wang, 2020; Liu, 2021; Ye et al., 2021; Wang et al., 2022) “Low” risk, 17 (Du et al., 2009; Wang, 2011; Zhao, 2012; Liu, 2013; Zhang et al., 2014a; Han, 2014; Zhu et al., 2015; Yan, 2017; Yuan, 2017; Chen and Qiu, 2018; Hang et al., 2018; Huang et al., 2018; Jiao et al., 2018; Xiong, 2018; Du et al., 2019; Liu et al., 2019; Wang, 2019)"Some concerns” risk and 9 (Jia and Liu, 2004; Zhang et al., 2014b; Huang and Fu, 2014; Zhang, 2014; Sun, 2015; Xin and Lin, 2016; Sun, 2019; Qiu et al., 2021)"High” risk which was an overall judgment for the result from the above 5 areas. More information was available in Supplementary Material.

**FIGURE 2:**
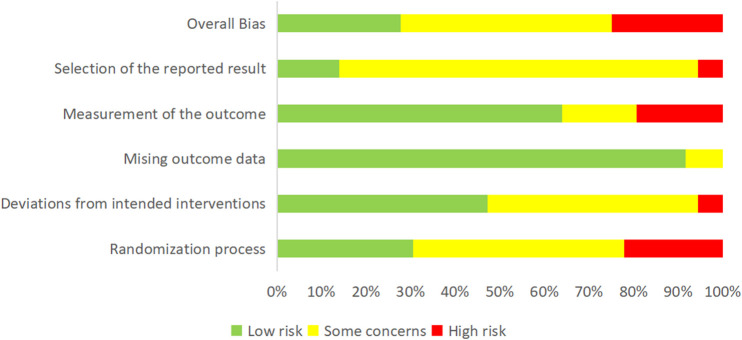
Risk of bias assessment in the included studies.

Based on the evaluations across the aforementioned five domains, the results were determined in line with the ROB2 operational logic. Specifically, 30.56% of the studies exhibited low risk, 47.22% presented medium risk, and 22.22% had high risk. These findings were depicted in Figure 2. For a more detailed risk of bias assessment for the RCTs, please refer to Table 2 and Table 3.

**TABLE 2:** The risk of bias assessment of the RCTs.

	Randomization process (%)	Deviations from intended interventions (%)	Mising outcome data (%)	Measurement of the outcome (%)	Selection of the reported result (%)	Overall Bias (%)
Low risk	30.56	47.22	91.67	63.89	13.89	27.78
Some concerns	47.22	47.22	8.33	16.67	80.56	47.22
High risk	22.22	5.56	-	19.44	5.56	25.00

**TABLE 3:** The data of ROB2 of risk of bias of included studies.

Study	Randomization process	Deviations from intended interventions	Mising outcome data	Measurement of the outcome	Selection of the reported result	Overall bias
Yu (2019)	Low	Low	Low	Low	Some concerns	Low
Sun (2015)	High	Some concerns	Low	High	Some concerns	High
Liu et al. (2020)	Low	Some concerns	Low	Low	Some concerns	Low
Li (2017)	Low	Some concerns	Low	Low	Some concerns	Low
Wang (2011)	Some concerns	Some concerns	Low	Low	Some concerns	Some concerns
Qiu et al. (2021)	High	Some concerns	Low	High	Some concerns	High
Xin and Lin (2016)	High	High	Low	Some concerns	Some concerns	High
Gao and Jin (2013)	Low	Low	Low	Low	Some concerns	Low
Zhang et al. (2014a)	Some concerns	Low	Low	Some concerns	Some concerns	Some concerns
Zhang et al. (2014b)	Some concerns	High	Low	Low	Some concerns	High
He et al. (2013)	High	Some concerns	Low	High	High	High
Liu (2013)	Some concerns	Low	Low	Some concerns	Some concerns	Some concerns
Xiong (2018)	Some concerns	Low	Low	Low	Some concerns	Some concerns
Chen and Qiu (2018)	Some concerns	Some concerns	Low	Some concerns	Some concerns	Some concerns
Huang et al. (2018)	Some concerns	Low	Low	Low	Some concerns	Some concerns
Jia and Liu (2004)	High	Some concerns	Some concerns	High	Some concerns	High
Ye et al. (2021)	Low	Low	Low	Low	Low	Low
Zhu et al. (2015)	Some concerns	Some concerns	Low	Low	Some concerns	Some concerns
Zhou (2013)	Low	Low	Low	Low	Some concerns	Low
Sun (2019)	High	Some concerns	Some concerns	High	Some concerns	High
Hao et al. (2020)	Low	Low	Low	Low	Low	Low
Wang (2020)	Low	Some concerns	Low	Low	Low	Low
Yuan (2017)	Low	Some concerns	Some concerns	Some concerns	Some concerns	Some concerns
Huang et al. (2018)	High	Some concerns	Low	High	Some concerns	High
Wang (2019)	Some concerns	Low	Low	Low	Some concerns	Some concerns
Hang et al. (2018)	Some concerns	Low	Low	Low	Some concerns	Some concerns
Zhang (2014)	Some concerns	Low	Low	High	High	High
Du et al. (2019)	Some concerns	Some concerns	Low	Low	Some concerns	Some concerns
Jiao et al. (2018)	Some concerns	Low	Low	Some concerns	Some concerns	Some concerns
Du et al. (2009)	Some concerns	Low	Low	Low	Some concerns	Some concerns
Zhao (2012)	Some concerns	Low	Low	Low	Some concerns	Some concerns
Yan (2017)	Some concerns	Some concerns	Low	Low	Low	Some concerns
Wang et al. (2022)	Low	Low	Low	Low	Some concerns	Low
Liu (2021)	Low	Some concerns	Low	Low	Low	Low
Han (2014)	High	Some concerns	Low	Low	Some concerns	Some concerns
Liu et al. (2019)	Some concerns	Low	Low	Low	Some concerns	Some concerns

### 3.4 Paired meta-analysis of the antihypertensive effective rate

**FIGURE 3:**
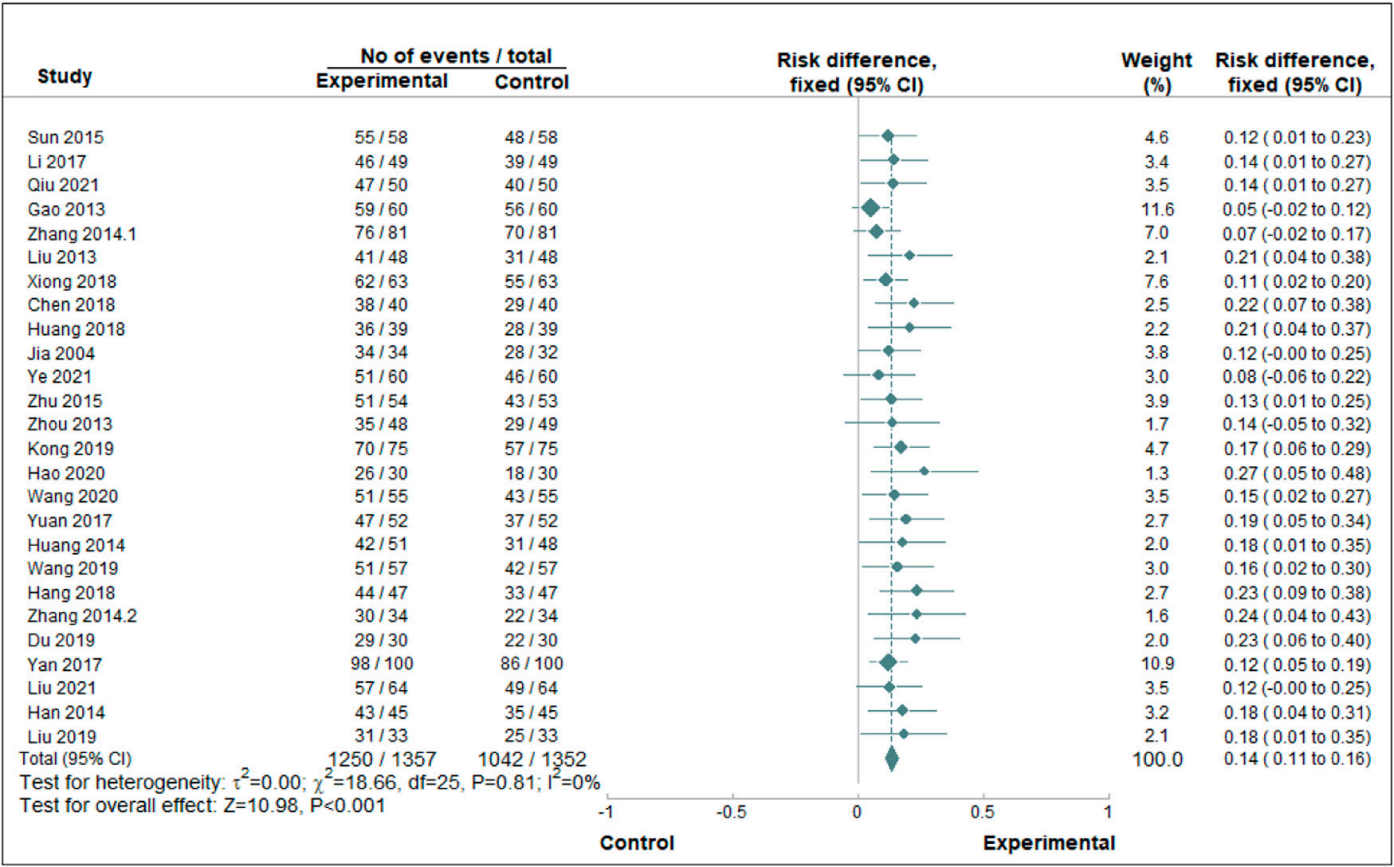
Paired meta-analysis of the antihypertensive effective rate. *Notes:* Chinse patent medicine + CCB vs. CCB.

**FIGURE 4:**
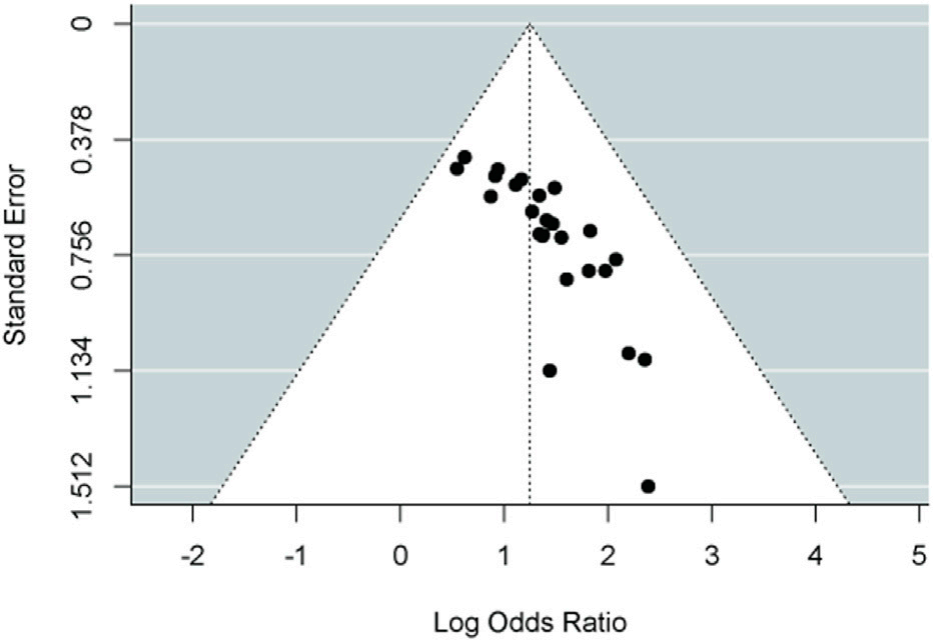
Funnel plot of paired meta-analysis of the antihypertensive effective rate.

A paired meta-analysis was conducted on the included studies using a fixed-effect model. The analysis indicated that the combination of Chinese patent medicine with CCBs yields greater efficacy in enhancing the antihypertensive effective rate compared to CCBs alone. This difference was statistically significant, as illustrated in Figure 3. To assess publication bias for the primary outcome’s effective rate, a funnel plot was generated. The plot revealed that most data points cluster around the center and top but display asymmetry, suggesting potential publication bias, as presented in Figure 4.

### 3.5 Evidence network map for outcomes

**FIGURE 5:**
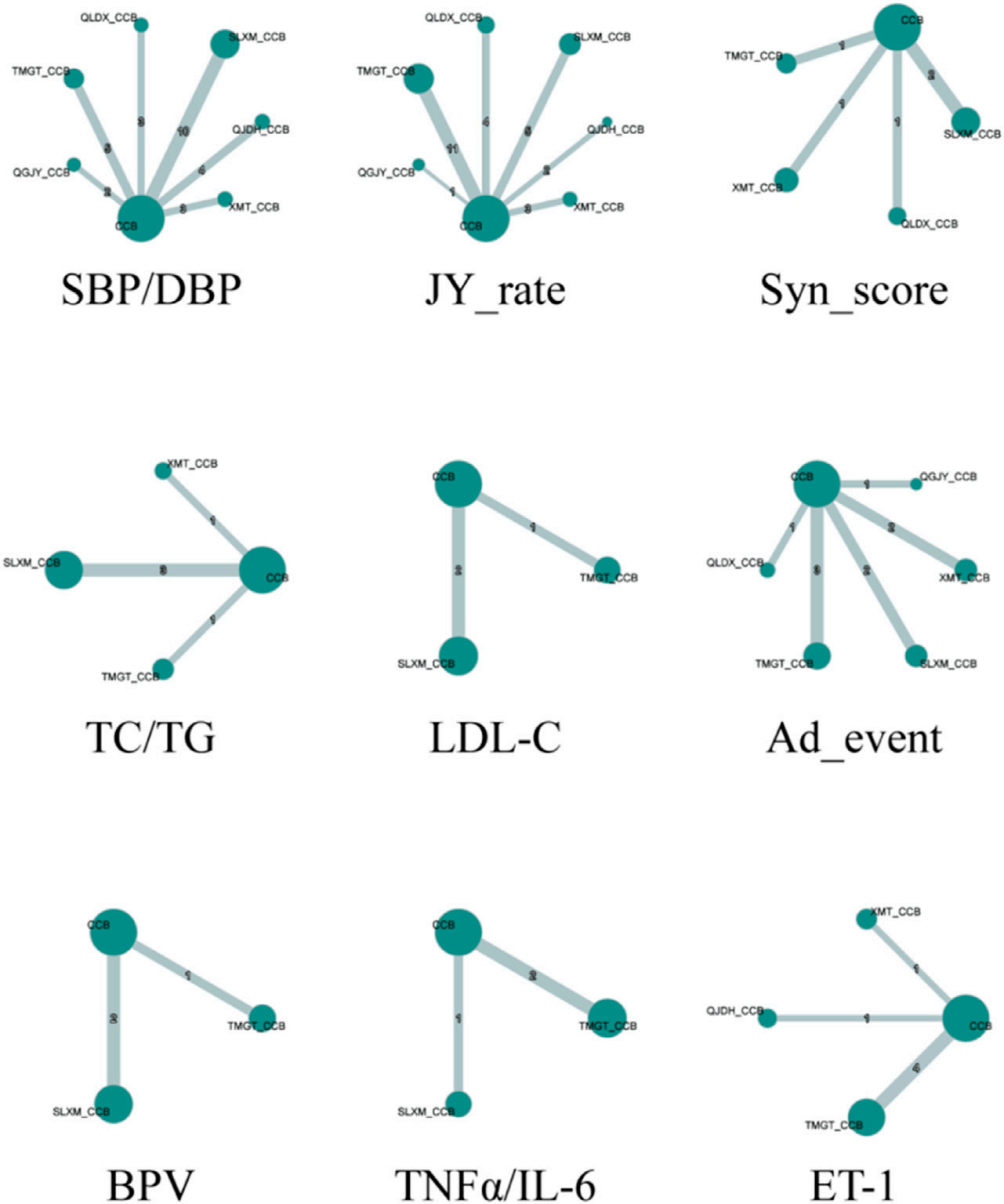
Network plot for the antihypertensive effective rate. *Notes*: SBP/DBP:Systolic blood pressure/Diastolic blood pressure; JY_rate: Rate of blood pressure decreases; Syn_score: TCM Syndrome score; TC/TG: Total cholesterol/Triglycerides; LDL-C: Low-density lipoprotein cholesterol; Ad_event: Adverse events; BPV: Blood pressure variability; TNFα/IL-6: tumor necrosis factor α/Interleukin- 6; ET-1: Endothelin-1.

An evidence network diagram was constructed to represent the 12 types of outcomes for 6 Chinese patent medicines combined with CCBs antihypertensive medications. Outcomes with consistent literature information were merged into a single diagram. Edges in the diagram were thicker when more RCTs are included. Points were larger when there’s a greater number of patients involved in those studies. All evidence networks and corresponding data are presented in Figure 5.

### 3.6 Bayesian network model using gemtc package

**FIGURE 6:**
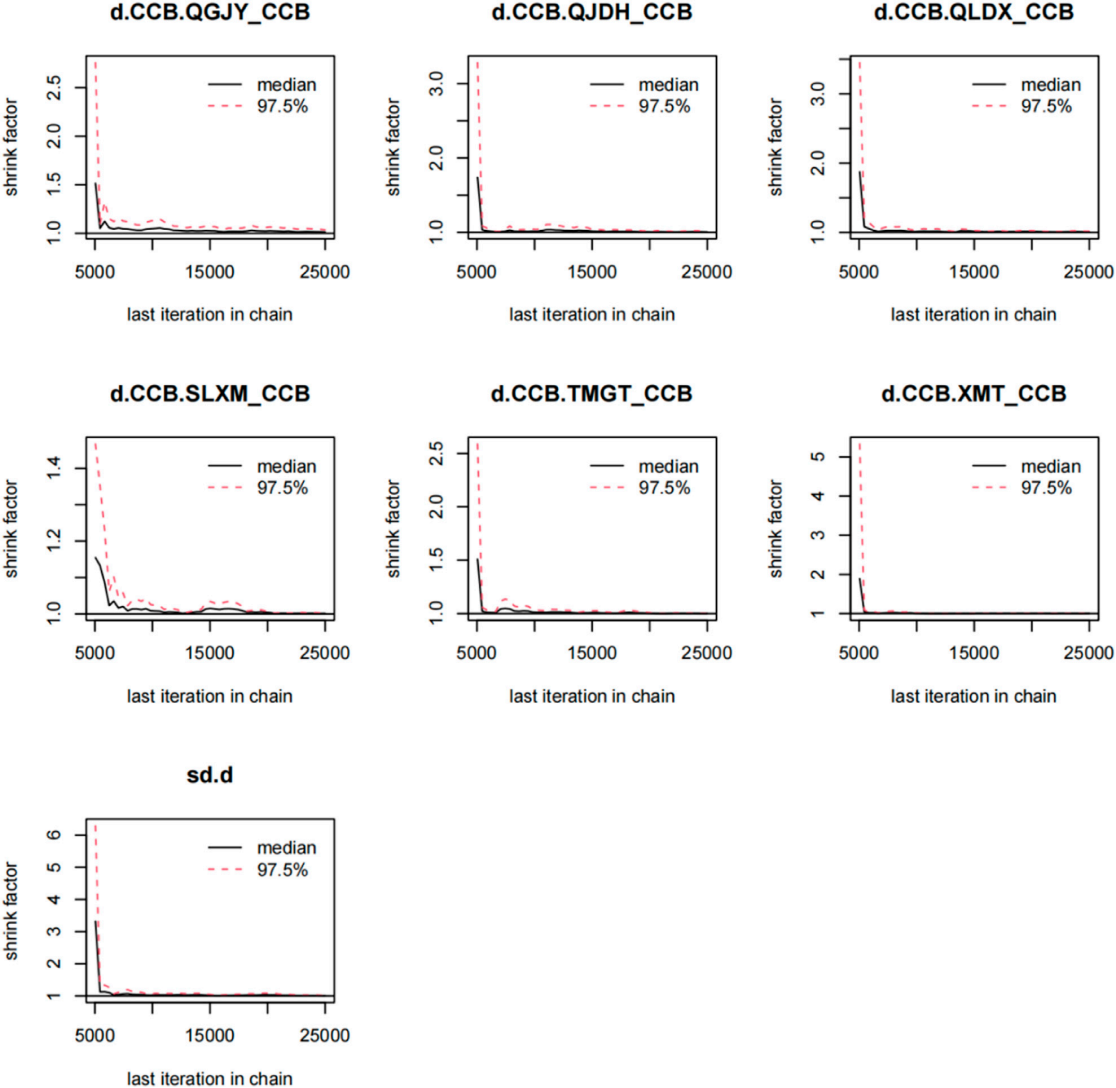
Gelman-Rubin-Brooks plot for rate of blood pressure decreases. *Notes*:Chinese patent drugs + CCB vs. CCB.

**FIGURE 7:**
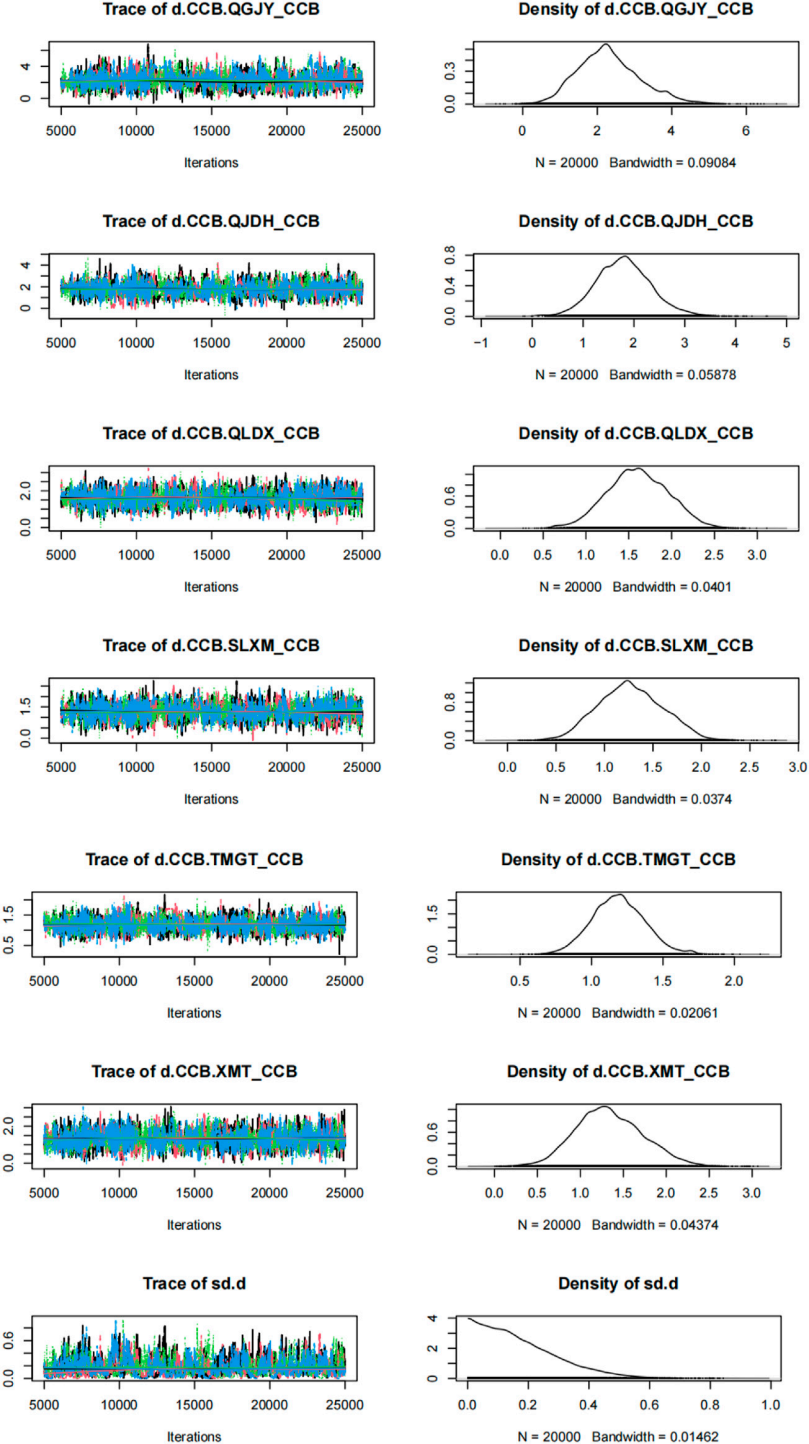
Time-series and density plots for rate of blood pressure decreases. *Notes*: Chinese patent drugs + CCB vs. CCB.

Utilizing the gemtc package, we built a Bayesian network model with the antihypertensive effective rate reduction as our focal point. After 5,000 preliminary iterations, the observed fluctuations in each Markov chain were minimal, signifying satisfactory convergence of the model. Consequently, there was no necessity to augment the calculation coefficient. Post the 5,000 iterations, the PSRF for every group rapidly converged to 1. In conjunction with the gemtc software, the calculated PSRF for all groups equaled 1.00. This indicated that the computational outcomes between distinct chains are consistent. The level of convergence was deemed satisfactory, ensuring that the established Bayesian model can effectively forecast subsequent results. These findings are illustrated in Figures 6, 7; Supplementary Figure S7; Supplementary Figure S8.

### 3.7 Results of the network meta-analysis

#### 3.7.1 Systolic blood pressure (SBP) analysis

**FIGURE 8:**
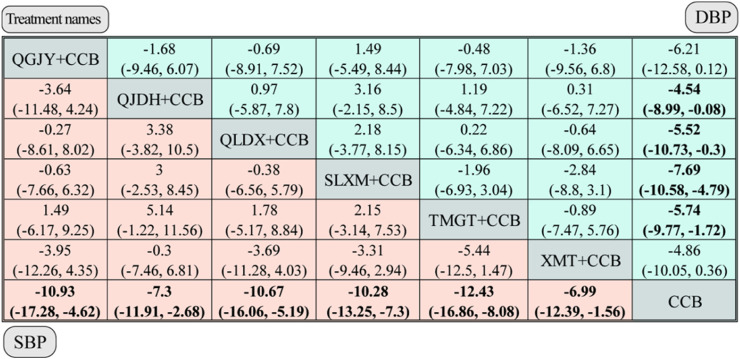
Network meta-analysis for SBP and DBP. *Notes*: different Chinese patent medicine + CCB vs. CCB. The comparison between different interventions is represented by MD and 95%*CrI*, and the ones in bold mean *p* < 0.05.

Our study, depicted in Figure 5, evaluated the impact of CCBs with various Chinese patent medicines on systolic blood pressure (SBP). The comprehensive results of the network meta-analysis are as follows:

QGJY [MD = −10.93, 95%*CrI* (−17.28,-4.62)], QJDH [MD = −7.3, 95%*CrI* (−11.91, −2.68)],QLDX [MD = −10.67,95%*CrI*(-16.06,-5.19)],SLXM [MD = −10.28,95%*CrI*(-13.25,−7.3)],TMGT [MD = −12.43,95%*CrI*(-16.86,−8.08)],XMT [MD = −6.99,95%*CrI*(-12.39,-1.56)]. These data are visualized in Figure 8. Each of the aforementioned combinations showcased a superior antihypertensive effect compared to the sole administration of CCBs, with statistical significance at *p* < 0.05.

**FIGURE 9:**
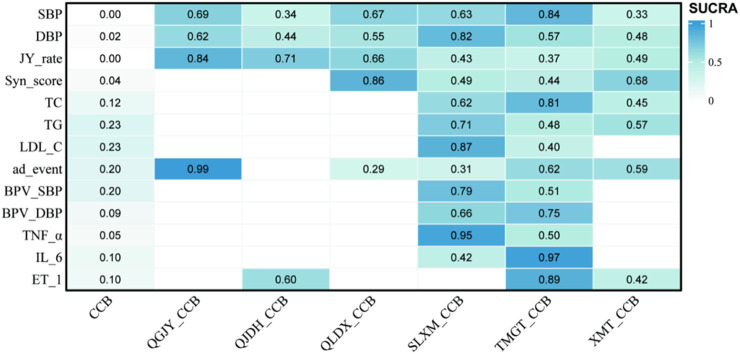
SUCRA score heat map of outcomes. *Notes*: SBP: systolic blood pressure; DBP: diastolic blood pressure; JY_rate: antihypertensive effect rate; Syn_score: TCM syndrome score; TC: total cholesterol; TG: triglyceride; LDL-C: low-density lipoprotein cholesterol; ad_event: adverse reactions/events; BPV_SBP: systolic blood pressure variability; BPV_DBP: diastolic blood pressure variability; TNF_α: tumor necrosis factor-α; IL_6: interleukin-6; ET_1: vascular endothelin-1.

Further insights are provided by the Surface Under the Cumulative Ranking (SUCRA) scores, which indicate the probable efficacy of each combination in reducing SBP. Tianma Gouteng Granule combined with CCB (TMGT + CCB) emerged as the most likely superior intervention, boasting an 83.99% score. In terms of efficacy, the ranking of the Chinese patent medicine combined with CCB is as follows:

TMGT + CCB (83.99%) > QGJY + CCB (68.51%) > QLDX + CCB (66.73%) > SLXM + CCB (63.29%) > QJDH + CCB (34.06%)>XMT + CCB (33.26%). These comprehensive rankings and data are visualized in Figure 9 and Supplementary Material.

Table 4 shows combined treatments based on outcomes, analyzed in conjunction with SUCRA.

**TABLE 4:** Combined treatments according to different outcomes ranked by SUCRA.

Outcomes	Recommended interventions
SBP	TMGT + CCB
DBP	SLXM + CCB
JY_rate	QGJY + CCB
Syn_score	QLDX + CCB
TC	TMGT + CCB
TG	SLXM + CCB
LDL_C	SLXM + CCB
ad_event	QGJY + CCB
BPV_SBP	SLXM + CCB
BPV_DBP	TMGT + CCB
TNF-α	SLXM + CCB
IL-6	TMGT + CCB
ET-1	TMGT + CCB

*Notes*: SBP: systolic blood pressure; DBP: diastolic blood pressure; JY_rate: antihypertensive effect rate; Syn_score: TCM, syndrome score; TC: total cholesterol; TG: triglyceride; LDL-C: low-density lipoprotein cholesterol; ad_event: adverse reactions/events; BPV_SBP: systolic blood pressure variability; BPV_DBP: diastolic blood pressure variability; TNF-α: tumor necrosis factor-α; IL-6: interleukin−6; ET-1: vascular endothelin-1.

#### 3.7.2 Diastolic blood Pressure (DBP) analysis

The network meta-analysis, depicted in Figure 5, assessed the impact of various Chinese patent medicines combined with CCBs on diastolic blood pressure (DBP). Here’s a breakdown of the findings: QJDH [MD = −4.54, 95%*CrI*(−8.99, −0.08)], QLDX [MD = −5.52, 95%*CrI*(-10.73, −0.3)], SLXM [MD = −7.69, 95%*CrI* (−10.58, −4.79)], TMGT [MD = −5.74, 95% *CrI* (−9.77, −1.72)]. These data are visualized in Figure 8.

However, two combinations, namely, QGJY [MD = −6.21, 95%CrI (−12.58, 0.12)] and XMT [MD = −4.86, 95%CrI (−10.05, 0.36)], did not show a statistically significant improvement over using CCB alone. Despite this, most of these combinations indicated a statistically significant superior antihypertensive effect compared to using only CCB (*p* < 0.05).

A deep dive into the SUCRA scores provided insights into the probable efficacy of each combination in reducing DBP. The results spotlighted SLXM + CCB as the most promising intervention with a score of 82.44%. In terms of efficacy, the Chinese patent medicines combined with CCB are ranked as:

SLXM + CCB(82.44%)>QGJY + CCB(62.17%)>TMGT + CCB(57.41%)>QLDX + CCB(54.85%)>XMT + CCB(47.76%)>QJDH + CCB (43.56%). These comprehensive findings and rankings are visualized in Figure 9.

#### 3.7.3 Antihypertensive effective rate analysis

**FIGURE 10:**
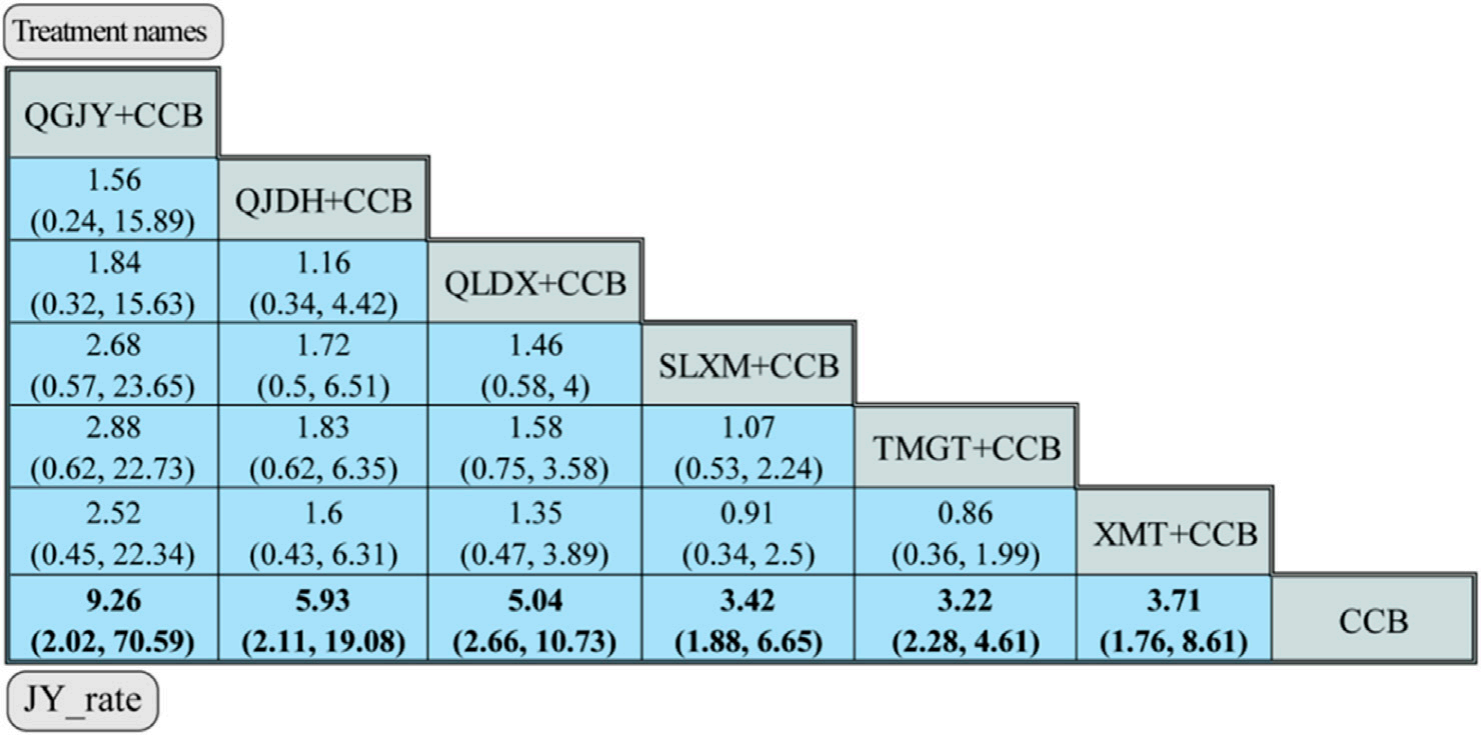
Network meta-analysis for JY_rate. *Notes*: different Chinese patent medicine + CCB vs. CCB. The comparison between different interventions is represented by MD and 95%*CrI*, and the ones in bold mean *p* < 0.05.

As presented in Figure 10, the network meta-analysis delved into the efficacy of various Chinese patent medicines when combined with calcium channel blockers (CCBs) to improve antihypertensive effective rates. Here are the key findings:

QGJY [OR = 9.26, 95% CrI (2.02, 70.59)], QJDH [OR = 5.93, 95% CrI (2.11, 19.08)], QLDX [OR = 5.04, 95%CrI (2.66, 10.73)], SLXM [OR = 3.42, 95%CrI (1.88, 6.65)], TMGT [OR = 3.22, 95%CrI (2.28, 4.61)], XMT [OR = 3.71, 95% CrI (1.76, 8.61)]. These combinations showcased a statistically significant advantage in antihypertensive efficacy when compared to using only CCB (*p* < 0.05).

The SUCRA scores further shed light on the probable efficiency of each combination in enhancing antihypertensive rates. Notably, QGJY + CCB emerged as the top contender with a remarkable score of 84.48%. Here’s the hierarchy based on efficacy:

QGJY + CCB (84.48%) >QJDH + CCB (70.89%) > QLDX + CCB (66.34%)>XMT + CCB (48.69%)>SLXM + CCB (42.62%)> TMGT + CCB (36.87%). These insightful findings are vividly portrayed in Figure 9.

See the secondary outcomes in Supplementary Material.

### 3.8 Safety evaluation

Safety is paramount when evaluating the efficacy of any medical treatment. In our analysis, 10 out of the total studies discussed potential adverse drug reactions/events. There was no significant difference in the rage of adverse events with no heterogeneity (Supplementary Figure S14). The most common adverse events included facial blushing, nauseous, palpitations, dizziness.

However, one of the challenges in consolidating these findings was the varied criteria each study used to determine and classify these adverse reactions/events. Due to these inconsistencies and to ensure clarity, we’ve opted for a descriptive analysis approach rather than a quantitative one.

For a detailed breakdown of the reported adverse reactions/events from each of these 10 studies, please refer to Table 5. This table provides insight into the type, frequency, and severity of reactions, giving both researchers and medical professionals a clearer picture of the safety profile of these treatments.

**TABLE 5:** Overview of drug safety information.

Study	Intervention	n	Adverse effects/events		P	Outcome
		E/C	E	C		
Li (2017)	SLXM + CCB vs. CCB	49/49	mild diarrhea、dizziness、palpitations in 3patients	nauseous、headache in 2 patients	0.646	Not mentioned
Zhang et al. (2014b)	SLXM + CCB vs. CCB	81/81	Palpitations、nauseous、dizziness、fatigue in 2 patients	diarrhoea、abdominal pain、insomsonia、facial blushing、sweat in 4 patients	>0.05	Not mentioned
Xiong (2018)	QLDX + CCB vs. CCB	63/63	facial blushing in 3 patients, palpitations in 2 patients, lower limb oedema in 2 patients, gingival hypertrophy in 1 patient	facial blushing in 4 patients, palpitations in 3 patients, lower limb oedema in 1 patient, gingival hypertrophy in 1 patient	>0.05	Not mentioned
Zhou (2013)	TMGT + CCB vs. CCB	63/64	hypotension in 2 patients, dry cough in 3 patients, facial blushing in 4 patients, ankle oedema in 3 patients	hypotension in 2 patients, dry cough in 4 patients, facial blushing in 3 patients, ankle oedema in 3 patients	>0.05	Not mentioned
Sun (2019)	TMGT + CCB vs. CCB	75/75	headache in 1 patient, nauseous in 2 patients, angina pectoris in 1 patient	Headache in 5 patients, nauseous in 6 patients, angina pectoris in 4 patients	0.012	Not mentioned
Wang (2020)	TMGT + CCB vs. CCB	55/55	adverse effects in 2 patients	adverse effects in 9 patients	<0.05	Not mentioned
Wang (2019)	TMGT + CCB vs. CCB	57/57	no obvious side effects	no obvious side effects	—	Not mentioned
Zhao (2012)	QGJY + CCB vs. CCB	40/40	no obvious side effects	facial blushing in 1 patient, headache in 3 patients	—	Not mentioned
Wang et al. (2022)	XMT + CCB vs. CCB	70/70	palpitations in 2 patients, headache in 1 patient, facial blushing in 1 patient, gastric distention in 1 patient	diarrhoea in 2 patients, gastric distention in 2 patients, palpitations in 1 patient, headache in 1 patient	0.753	Not mentioned
Liu (2021)	XMT + CCB vs. CCB	64/64	facial blushing in 1 patient, increased heart rate in 1 patient	facial blushing in 1 patient, increased heart rate in 6 patients, abdominal distension in 5 patients	<0.05	Not mentioned

### 3.9 Sensitivity analysis

Using the leave-one-out sensitivity anlysis, we have revealed and examined that no single study altered the pooled effect of RR and MD. Most of the summary results remained the same after systematically excluding specific studies one bye one proving the robustness of our results. Additional results of sensitivity analyses of SBP, DBP, antihypertensive and adverse rate were provided in Supplement.

### 3.10 Subgroup analysis

As the amount of data for subgroup analysis was insufficient, subgroup analysis in our study were conducted of SBP, DBP and antihypertensive effective rate. Specific of Chinese patent medicines included SLXM, TMGT, QLDX, QGJY, QJDH and XMT. Statistically significant differences were observed between subgroups, and heterogeneity was reduced in the subgroups (Supplementary Material), suggesting that the type of Chinese patent medicines may be the source of heterogeneity. Other results of the bias analysis were presented in Supplementary Material.

## 4 Discussion

### 4.1 Global prevalence of EH and insights into modern and traditional therapeutic approaches

The global implications of elevated blood pressure are concerning. Among the 3.5 billion adults worldwide, blood pressure levels in many are below the optimal range. Alarmingly, about 874 million adults have systolic blood pressure exceeding 140 mmHg, translating to nearly one-quarter of the global adult population grappling with EH (1). The underpinnings of abnormal blood pressure can primarily be attributed to irregular blood pressure regulation. Ensuring physiological blood pressure requires the synchronized functioning of various elements within an intricate neurohumoral system. This includes factors such as the renin-angiotensin-aldosterone system, natriuretic peptides, endothelium, sympathetic nervous system, as well as inflammation and immune responses (Oparil et al., 2018).

Modern medicine has recognized the importance of vascular tone in regulating blood pressure and distributing blood flow within the body. Among the therapeutic arsenal, CCBs are particularly effective for hypertensive vessels, owing to their specificity to increased inactivated channels and the presence of a greater number of these channels (Morel and Godfraind, 1987). Beyond mere blood pressure control, CCBs also play a pivotal role in preventing the structural changes induced by EH in both the heart and arteries.

Complementing this modern understanding, TCM provides a unique perspective. While EH is not directly named in TCM, its clinical manifestations align with symptoms described as “dizziness” and “headache”. The TCM diagnosis points to various pathogenic factors, such as hyperactivity of liver yang, syndrome of static blood blocking collaterals, liver wind stirring, and kidney essence deficiency. Guided by its foundational principles of holistic treatment and syndrome differentiation, TCM emphasizes tailored treatments. Consequently, antihypertensive Chinese patent medicines, when prescribed in line with syndrome differentiation, have demonstrated efficacy in reducing blood pressure and alleviating clinical symptoms.

Recognizing and addressing EH is paramount, not just for individual health, but from a broader public health perspective. As supported by a 2009 meta-analysis, optimal management of EH, whether through modern antihypertensive drugs or TCM approaches, is instrumental in curtailing cardiovascular risks and fostering longevity in the global populace (Law et al., 2009).

### 4.2 Comparative efficacy of combined treatments for EH

Building on our understanding of the global prevalence of EH and the synergy of modern and traditional therapeutic approaches, it is essential to delve into the efficacy of combined treatments.

In the paired meta-analysis, our data presents compelling evidence supporting the enhanced therapeutic efficacy of combining Chinese patent medicine with CCBs in addressing EH. Specifically, when compared to the CCB-only group, the combined treatment approach exhibited a notably improved curative effect (RD = 0.14, 95%CI = 0.11, 0.16). Remarkably, this outcome demonstrated minimal heterogeneity (I^2^ = 0%).

Broadening our lens to the network meta-analysis, further differentiations emerge regarding the efficacy of various combinations:

SBP Reduction: Tianma Gouteng Granule combined with CCB stands out in its efficacy.

DBP Reduction: The combination of Songling Xuemaikang Capsule with CCB takes the lead.

Antihypertensive Efficacy: Qinggan Jiangya Capsule, when combined with CCB, showcases superior results.

TCM Syndrome Score: Patients treated with Qiangli Dingxuan Tablet combined with CCB observed the most significant reduction.

Lipid Profile Improvements: While Tianma Gouteng Granule and CCB excelled in reducing TC, the pairing of Songling Xuemaikang Capsule with CCB emerged as more potent in lowering LDL-C.

Inflammatory Marker Reduction: In terms of mitigating TNF-α and IL-6 levels, the combination of Songling Xuemaikang Capsule with CCB proved most effective.

It is crucial to note that the number of RCTs concerning secondary clinical outcomes was limited, which may impact the robustness of comparisons in those areas.

These insights underscore the importance of nuanced, individualized therapeutic strategies. The differential efficacy outcomes of the varied combinations offer clinicians a spectrum of options, allowing them to tailor EH management according to patient-specific needs, bridging the time-tested wisdom of TCM with the precision of modern pharmacology.

### 4.3 Mechanisms underpinning the efficacy of TCM capsules in EH management

Having highlighted the comparative efficacy of combined treatments for EH, it becomes imperative to probe deeper into the mechanisms and metabolites of key TCM capsules that stood out in the meta-analysis. This can provide insights into how these traditional remedies potentiate the effects of modern drugs like CCBs.

Xinmaitong Capsule: This capsule is known for promoting blood circulation, clearing blood stasis, and nourishing the heart. This intricate formulation boasts a blend of medicinal botanical drugs, including but not limited to, *Angelica sinensis (Oliv.) Diels, Salvia miltiorrhiza Bunge, Ilex pubescens Hook. and Arn.*, and *Pueraria lobata (Willd.) Ohwi.* Research by Wei Chengke (Wei et al., 2017) showed that this oil can protect vascular cells and help reduce blood pressure. Another significant metabolite, Tanshinone ⅡA from *S. miltiorrhiza Bunge*, aids in blood circulation (Li, 2017). Li Wendi’s study (Li and Sun, 2021)further highlighted its ability to regulate blood pressure, possibly by interacting with certain cellular pathways.

Tianma Gouteng Granule: Recognized for its calming and heat-clearing properties, its primary botanical drugss are *Gastrodia elata Bl.* and *Uncaria rhynchophylla (Miq.) Miq*., from Gastrodia, has potent antihypertensive effects (Chen et al., 2016). It has been shown to counteract certain vasoconstricting agents and promote vasodilation, leading to reduced blood pressure (Shan et al., 2017). Additionally, Cheng Xiankun’s research (Cheng et al., 2019) highlighted rhynchophylline’s capacity to optimize internal circulation and manage blood pressure in hypertensive model rats.

Songling Xuemaikang Capsule: This capsule calms the liver and subsiding yang, tranquillizing with heavy prescription, promotes blood circulation, and removes blood stasis. Comprising mainly of Pinea *Wolf* and Pueraria lobata (*Willd.*) *Ohwi*, etc., clinical studies have shown its efficacy in treating mild hypertension, with the potential to replace control drugs (Lai et al., 2022). Its mechanism involves regulating the RAAS pathway (Liu et al., 2015), modulating gene expression (Zhao et al., 2013), and safeguarding vascular endothelial cells by inhibiting specific cellular pathways. It further mitigates oxidative stress injuries in rat aorta through modulating CAV1 and IGF1R gene expressions (Shi et al., 2018).

Qinggan Jiangya Capsule: Known for its liver-calming and heat-clearing effects, its main botanical drugs are *Polygonum muliflorum Thunb*. and *S. miltiorrhiza Bunge.* Wang Xiayun’s study (Wang et al., 2017) emphasized its role in vascular dilation and reducing specific vascular growth factors. Liang Yanfei (Liang et al., 2021) further showcased its ability to decrease blood pressure while ensuring liver, kidney, and heart functions remain intact.

Qiangli Dingxuan Tablet: This tablet is designed to calm the liver wind. Main botanical drugs include *G. elata Blume* and Eucommia ulmoides Oliv., which have numerous antihypertensive metabolites like lignans and phenylpropanoids. Research has indicated that these metabolites can work through various pathways to manage blood pressure, such as inhibiting specific enzymes and combating vasoconstriction (Taubert et al., 2002; Gu et al., 2011).

### 4.4 Clinical significance

Past meta-analyses have compared Chinese patent medicines combined with western medicines in the treatment of EH, which showd significant reduction in both SBP and DBP compared to western medicines. There were significant beneficial effects in SLXM combined with antihypertensive drugs compared to the antihypertensive drugs using alone. However, no compelling evidence was found to demonstrate its superior efficacy compared to other Chinese patent medicines. RCTs about QLDX for the treatment of EH showed that SBP and DBP were significantly lower than in the placebo group (Yang et al., 2015; Ji et al., 2022; Zhang et al., 2022; Lin et al., 2023). While our results showed that TMGT and SLXM combined with CCB showed more effective in our NMA, and QLDX played an important role in reducing TCM Syndrome Score.

In furnishing healthcare professionals with a solid, evidence-based framework for treatment decision-making, the research challenges the prevailing one-size-fits-all medical paradigm, advocating for an alignment more intimately connected with the foundational doctrines of TCM. Based on our evidence, clinicians choose the most appropriate treatment plan based on the patient’s specific situation, enabling individualized medicine. The ideal integration of Chinese patent medicine and CCBs pivots on specific symptomatology, as discerned through the seasoned expertise of traditional Chinese medical practitioners. Grounded in the comprehensive principles of TCM and reflecting a tangible application in ethnopharmacological exploration, the findings accentuate the indispensability of personalized medical interventions.

Based on the results of our study, not only can provide high quality evidence for the formulation of clinical practice guidelines for EH, but also can guide clinical practice, and is of great significance for clinicians to choose proprietary Chinese patent medicines.

### 4.5 Limitations, biases, and implications for future trials

While this study offers the most updated review and network meta-analysis concerning Chinese patent medicine combined with CCB in the treatment of EH, several limitations merit attention. Firstly, the included RCTs did not retrieve the registration scheme, with the allocation concealment and blinding methods often omitted, potentially leading to biases. Furthermore, there’s a limited number of RCTs available for certain secondary outcomes, emphasizing the need for more comprehensive research and potentially undermining the credibility of some findings due to potential publication bias. And the number of studies and the patients in studies was limited, and many studies were not high quality and showed a degree of heterogeneity. There was an asymmetry in funnel plots, which is not necessarily a result of publication bias, but rather higher efficacy in small trials than in large trials for a variety of reasons.

It is also worth noting that all the included RCTs are exclusively Chinese literature, which means the study lacks data in other languages. This linguistic focus might introduce inherent biases or overlook crucial global perspectives. Additionally, potential heterogeneity in the clinical environments of the trials could introduce inconsistencies, although the consistency of the paired meta-analysis results of combined western medicine mitigates some of these concerns.

In addition, most of the patients in literature were middle-aged and elderly, and there were few studies on the efficacy of young EH patients which could lead to bias. Besides, whether the different duration of intervention causes the difference in efficacy should be studied which was absent in our study.

According to the SUCRA ranking in our study, different proprietary Chinese patent medicines had advantages in improving different outcomes, which may involve the simultaneous use of multiple drugs in clinical practice. Therefore, when the therapeutic effect is well established, economic evaluation such as cost-benefit analysis should be combined to provide valuable insights into the feasibility of these combined therapies. And for western doctors, treatment based on syndrome differentiation may be difficult, if the decision is only according to the outcomes, the syndrome will be ignored, and there may be wrong medication.

Looking forward, it is imperative to expand the linguistic and cultural scope of research. Future endeavors should incorporate studies from diverse linguistic and cultural backgrounds, ensuring a comprehensive global perspective. A deeper exploration into the molecular mechanisms through experimental research could elucidate the cellular interactions when blending these traditional medicines with western antihypertensives. Broadening clinical trials to target a wider variety of hypertensive patient profiles can pave the way to determine the best combinations for specific patient needs. Moreover, once therapeutic efficacy is well established, economic assessments such as cost-benefit analyses could offer valuable insights into the financial viability of these combined treatments, especially in settings where resources are limited.

However, the silver lining in this study is the innovative comparison of the curative effects of different Chinese patent medicines when combined with a specific type of antihypertensive western medicine. This provides clinicians with a more personalized approach to treatment, bolstered by our evidence-based analysis. As a beacon for future research, this study underscores the significant potential of integrating traditional practices with modern medicine, paving the way for more harmonized and effective healthcare solutions.

## 5 Conclusion

In conclusion, the results of our network meta-analysis provide evidence of the need for EH. This thorough review decisively underscores the nuanced therapeutic potentials and variances when integrating Chinese patent medicine with CCBs in the treatment of EH. The effectiveness of such combinations distinctly depends on the particular Chinese patent medicine and the unique clinical presentations of patients. Compared to traditional western therapy, the addition of Chinese patent medicine can simultaneously reduce cardiovascular risk factors, lower blood pressure, improve eradication rates and reduce side effects. Notably, Tianma Gouteng Granule and Songling Xuemaikang Capsule combined with CCB have the most prominent overall efficacy. The results of this study should be referenced by policymakers and the formulation of clinical practice guidelines, and should be applied in clinical practice in treatment of essential hypertension.

## Figures and Tables

**Table:** 

**ACEI**	angiotensin converting enzyme inhibitors
**Ad_event**	adverse drug reaction events
**ARB**	angiotensin II receptor blockers
**BPV**	blood pressure variability
**C**	control group
**CAV1**	caveolin 1
**CCB**	calcium channel blockers
**CI**	confidence interval
**CNKI**	China National Knowledge Infrastructure
**CrI**	credible interval
**CVD**	cardiovascular disease
**D**	day
**DBP**	diastolic blood pressure
**E**	experimental group
**EH**	essential hypertension
**ET-1**	vascular endothelin-1
**IGF1R**	insulin like growth factor 1 receptor
**IL-6**	interleukin-6
**JY_rate**	antihypertensive effective rate
**LDL-C**	low-density lipoprotein cholesterol
**MD**	mean difference
**NMA**	network meta-analysis
**OR**	odds ratio
**PPARγ**	peroxisome proliferator-activated receptor γ
**PRISMA**	Preferred Reporting Items for Systematic Reviews and Meta-Analysis
**PSRF**	potential scale reduction factor
**QGJY**	Qinggan Jiangya Capsule
**QJDH**	Qingju Dihuang Pill
**QLDX**	Qiangli Dingxuan Tablet
**RAAS**	rein angiotensin aldosterone system
**RCTs**	randomized controlled trials
**RD**	risk difference
**ROB2**	risk of bias 2
**SBP**	systolic blood pressure
**SD**	standard deviation
**SLXM**	Songling Xuemaikang Capsule
**SUCRA**	surface under the cumulative ranking
**Syn_score**	TCM syndrome score
**T**	treatment time
**TC**	total cholesterol
**TCM**	Traditional Chinese Medicine
**TG**	triglyceride
**TMGT**	Tianma Gouteng Granule
**TNFα**	tumor necrosis factor-α
**VIP**	China Science and Technology Journal Database
**W**	week
**XMT**	Xinmaitong Capsule

## Data Availability

The raw data supporting the conclusions of this article will be made available by the authors, without undue reservation.
