# Supplementary material for: Chinese patent medicine combined with calcium channel blockers in the treatment of essential hypertension:a Bayes network meta-analysis and systematic review
Source: Front Pharmacol. 2024 Mar 15;15:1321405. doi: 10.3389/fphar.2024.1321405 (PMC10978809; doi:10.3389/fphar.2024.1321405)
Supplement: Supplementary file 1 [file Table4.pdf]

Supplementary appendix

(Figures)

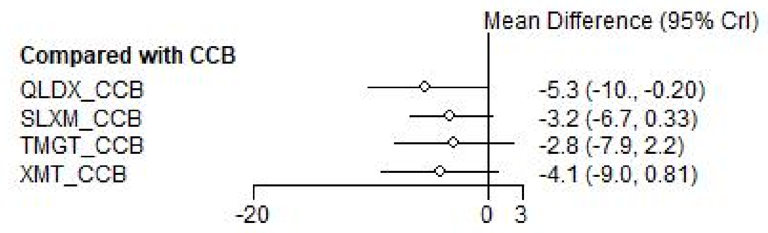

Figure S1 Forest plot for Syn_score

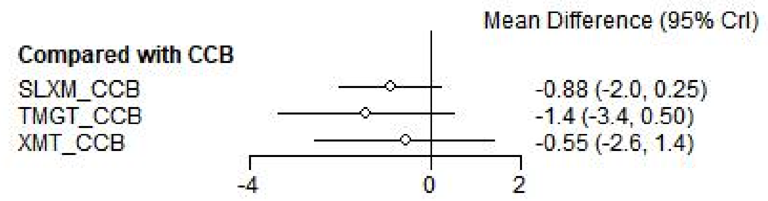

Figure S2 Forest plot for TC

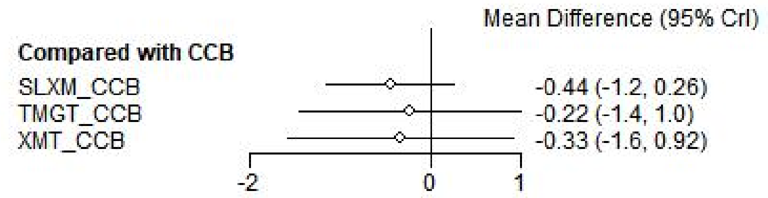

Figure S3 Forest plot for TG

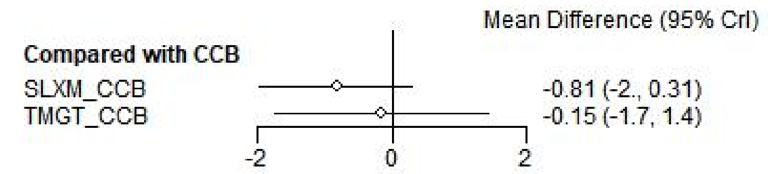

Figure S4 Forest plot for LDL-C

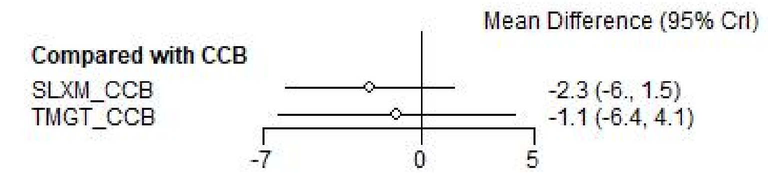

Figure S5 Forest plot for SBPV

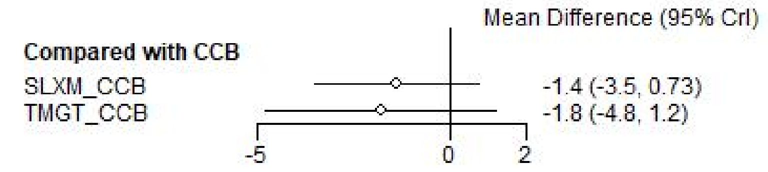

Figure S6 Forest plot for DBPV

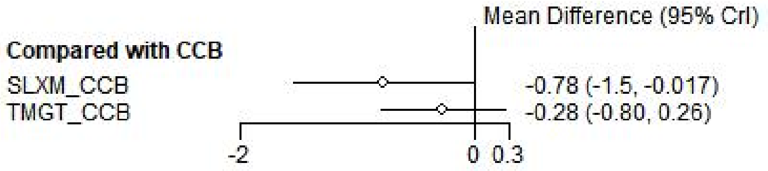

Figure S7 Forest plot for TNF-α

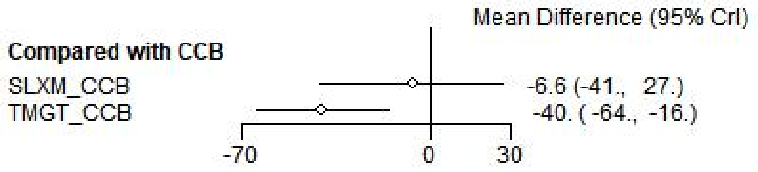

Figure S8 Forest plot for IL-6

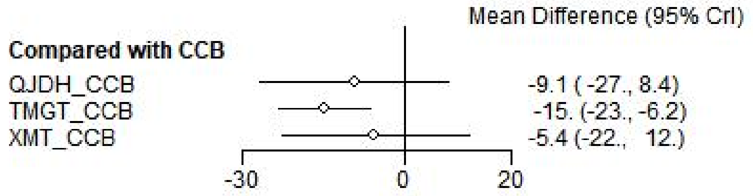

Figure S9 Forest plot for ET-1

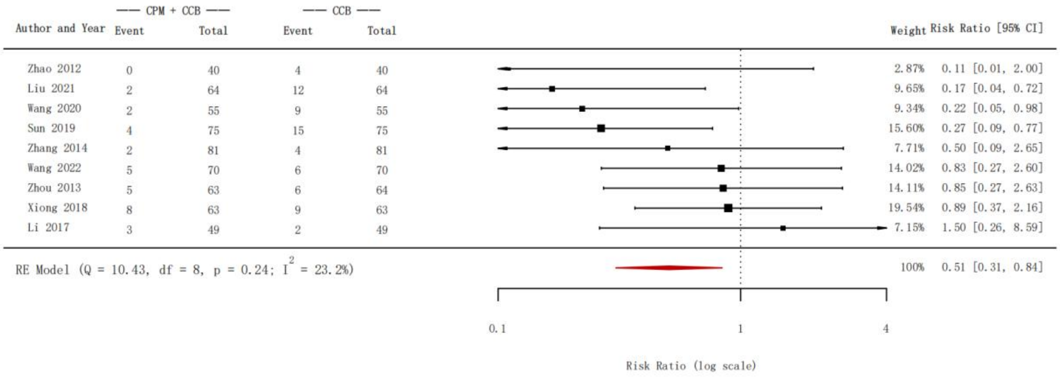

Figure S10 Forest plot for adverse drug reaction events

A.

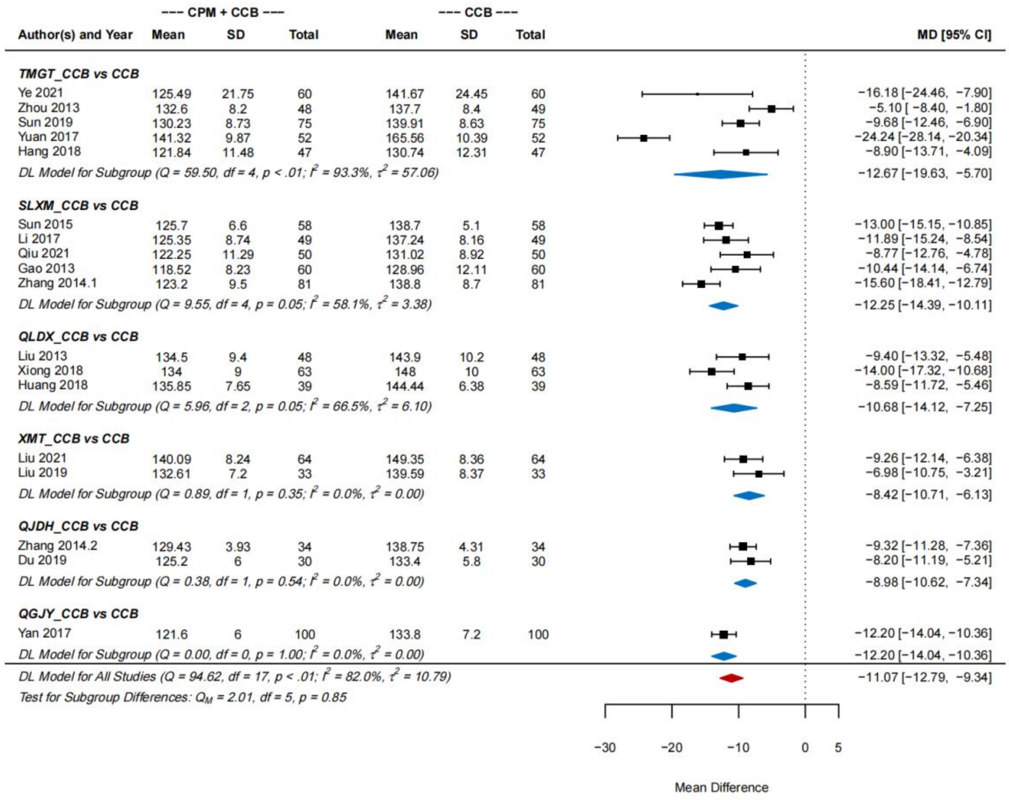

B.

Author(s) and Year	--- CPM + CCB --- Mean	SD	Total	--- CCB --- Mean	SD	Total	MD [95% CI]
TMGT_CCB vs CCB							
Ye 2021	73.91	8.65	60	85.25	9.66	60	−11.34 [−14.62, −8.06]
Zhou 2013	82.7	5.7	48	83.2	6	49	−0.50 [−2.83, 1.83]
Sun 2019	84.61	8.38	75	85.53	7.18	75	−0.92 [−3.42, 1.58]
Yuan 2017	82.31	3.58	52	89.91	4.56	52	−7.60 [−9.18, −6.02]
Hang 2018	80.43	5.56	47	89.01	5.82	47	−8.58 [−10.88, −6.28]
DL Model for Subgroup ($Q = 56.14$, $df = 4$, $p < .01$; $I^2 = 92.9\%$, $\tau^2 = 17.61$)							−5.74 [−9.58, −1.90]
SLXM_CCB vs CCB							
Sun 2015	78.5	4.1	58	89.8	4.6	58	−11.30 [−12.89, −9.71]
Li 2017	79.41	7.36	49	88.19	7.69	49	−8.78 [−11.76, −5.80]
Qiu 2021	74.02	5.83	50	77.25	5.38	50	−3.23 [−5.43, −1.03]
Gao 2013	81.49	10.12	60	87.97	9.95	60	−6.48 [−10.07, −2.89]
Zhang 2014.1	78.1	6.2	81	89.1	6.3	81	−11.00 [−12.92, −9.08]
DL Model for Subgroup ($Q = 40.59$, $df = 4$, $p < .01$; $I^2 = 90.1\%$, $\tau^2 = 12.06$)							−8.24 [−11.48, −5.00]
QLDX_CCB vs CCB							
Liu 2013	75.1	5.3	48	84.4	6.2	48	−9.30 [−11.61, −6.99]
Xiong 2018	75	5	63	86	7	63	−11.00 [−13.12, −8.88]
Huang 2018	85.85	4.8	39	89.59	4.9	39	−3.74 [−5.89, −1.59]
DL Model for Subgroup ($Q = 23.92$, $df = 2$, $p < .01$; $I^2 = 91.6\%$, $\tau^2 = 13.73$)							−8.01 [−12.39, −3.63]
XMT_CCB vs CCB							
Liu 2021	85.97	2.78	64	91.35	3.12	64	−5.38 [−6.40, −4.36]
Liu 2019	75.42	7.03	33	79.8	8.11	33	−4.38 [−8.04, −0.72]
DL Model for Subgroup ($Q = 0.27$, $df = 1$, $p = 0.61$; $I^2 = 0.0\%$, $\tau^2 = 0.00$)							−5.31 [−6.29, −4.32]
QJDH_CCB vs CCB							
Zhang 2014.2	77.92	2.17	34	86.83	2.52	34	−8.91 [−10.03, −7.79]
Du 2019	80.3	2.6	30	88.1	3.5	30	−7.80 [−9.36, −6.24]
DL Model for Subgroup ($Q = 1.28$, $df = 1$, $p = 0.26$; $I^2 = 22.2\%$, $\tau^2 = 0.14$)							−8.49 [−9.55, −7.44]
QGJY_CCB vs CCB							
Yan 2017	73.6	9	100	82.6	7.3	100	−9.00 [−11.27, −6.73]
DL Model for Subgroup ($Q = 0.00$, $df = 0$, $p = 1.00$; $I^2 = 0.0\%$, $\tau^2 = 0.00$)							−9.00 [−11.27, −6.73]
DL Model for All Studies ($Q = 169.24$, $df = 17$, $p < .01$; $I^2 = 90.0\%$, $\tau^2 = 8.47$)							−7.22 [−8.67, −5.77]
Test for Subgroup Differences: $Q_M = 2.47$, $df = 5$, $p = 0.78$							

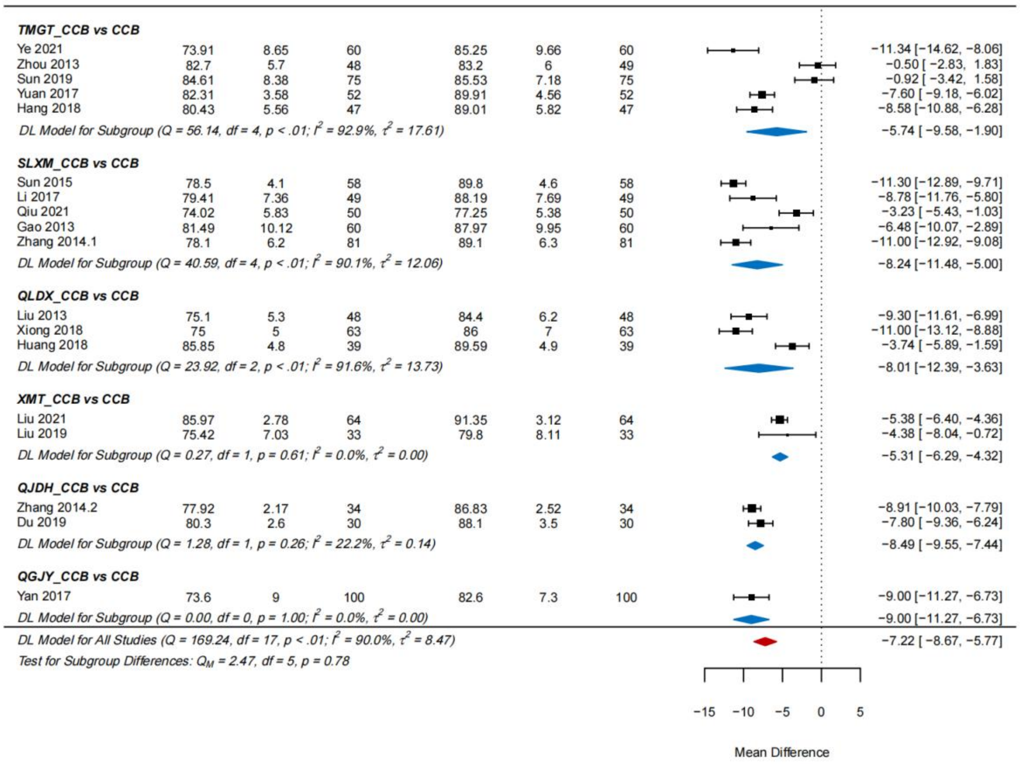

C.

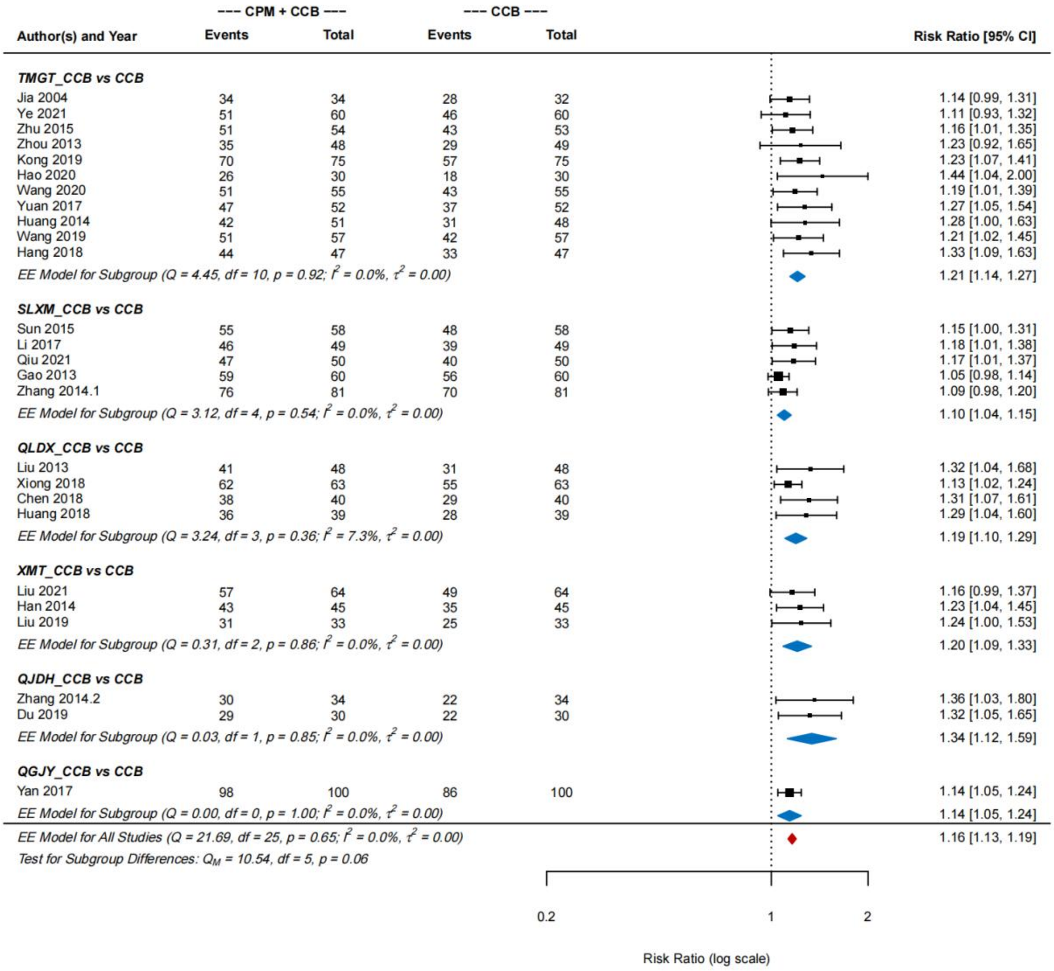

Figure S11 Subgroup analysis

Notes:A. SBP; B. DBP; C. antihypertensive effective rate

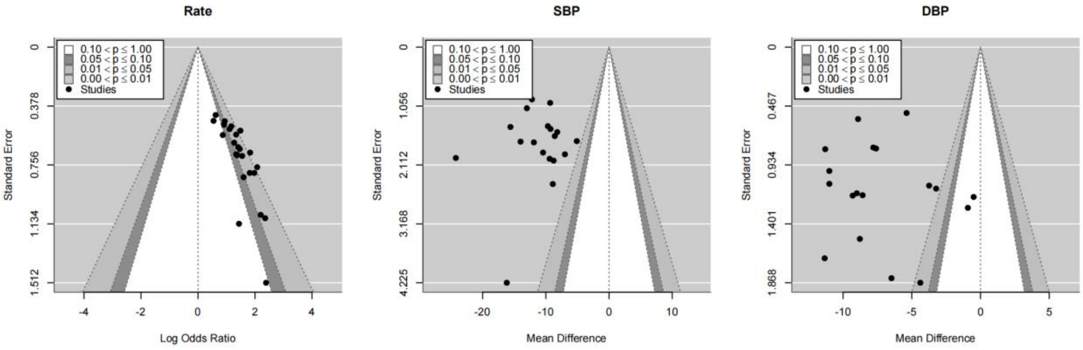

Figure S12 Contour-enhanced funnel plot

Notes:Rate: antihypertensive effective rate

A.

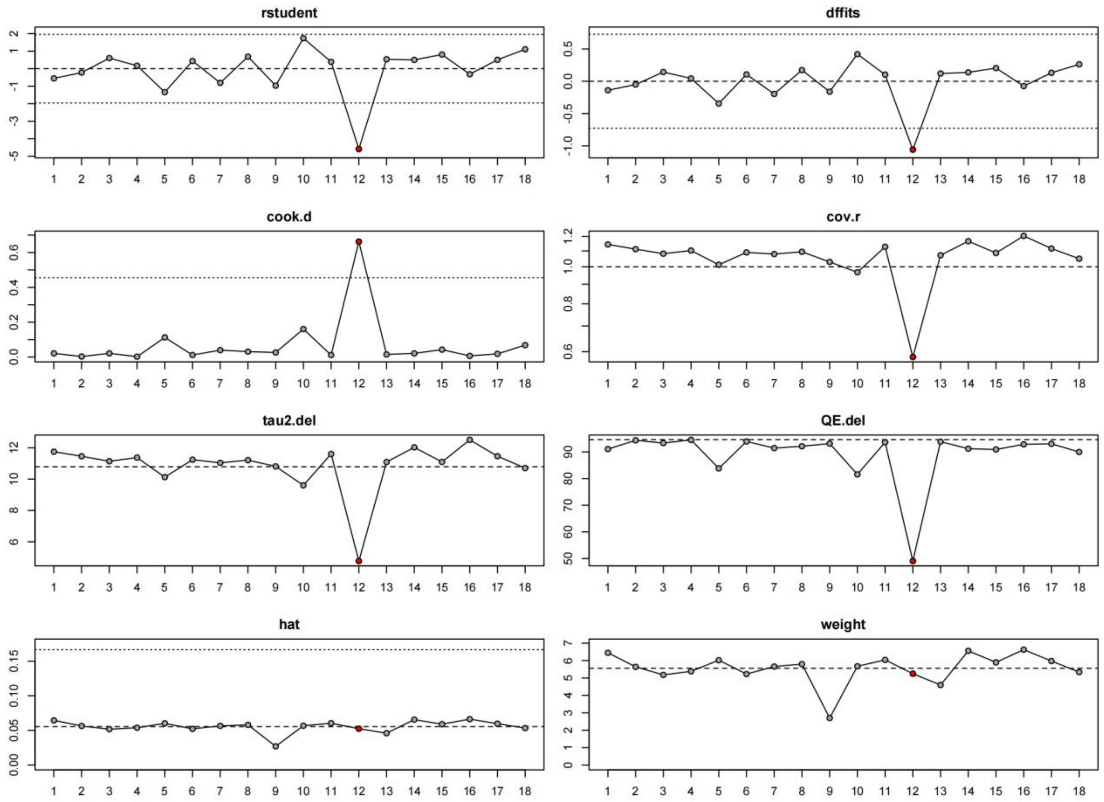
rstudent
dffits
cook.d
cov.r
tau2.del
QE.del
hat
weight

B.

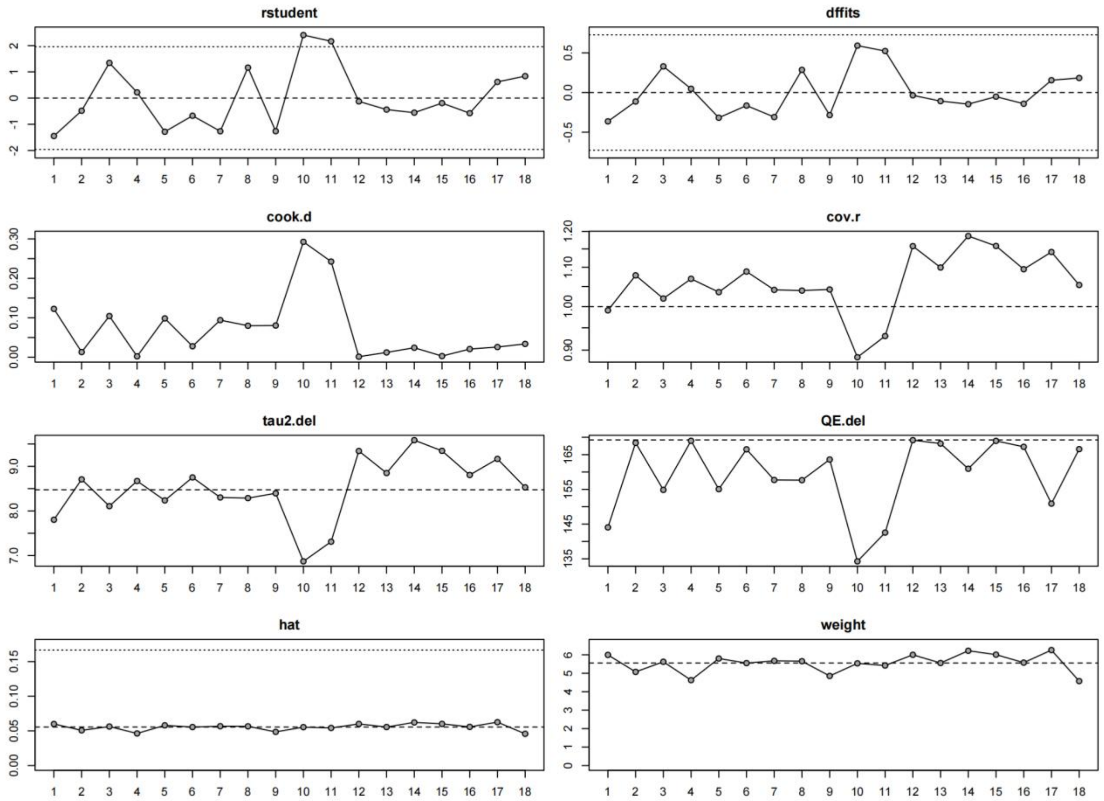
rstudent
dffits
cook.d
cov.r
tau2.del
QE.del
hat
weight

C.

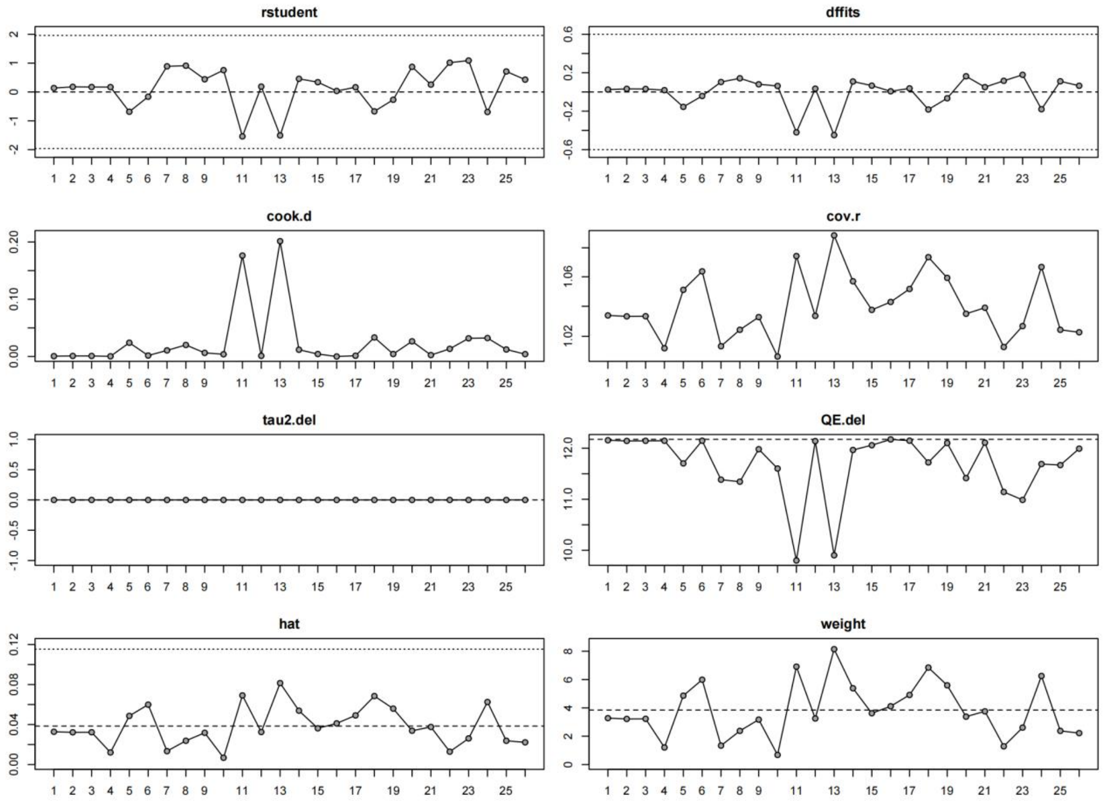

Figure S13 Plot of Influence Diagnostics

Notes:A. SBP; B. DBP; C. antihypertensive effective rate
