# Supplementary material for: Chinese patent medicine combined with calcium channel blockers in the treatment of essential hypertension:a Bayes network meta-analysis and systematic review
Source: Front Pharmacol. 2024 Mar 15;15:1321405. doi: 10.3389/fphar.2024.1321405 (PMC10978809; doi:10.3389/fphar.2024.1321405)
Supplement: Supplementary file 2 [file Table2.DOCX]

**Annex on the safety of Chinese patent medicine**

**(sourced from literature)**

**1.Songling Xuemaikang Capsule**

Meng T, Wang P, Xie X, Li T, Kong L, Xu Y, Cao K, Gao Y, He Q, Lai X. Efficacy and safety of Songling Xuemaikang capsule for essential hypertension: A systematic review and meta-analysis of randomized controlled trials. Phytomedicine. 2022 Dec;107:154459. doi: 10.1016/j.phymed.2022.154459. Epub 2022 Sep 20. PMID: 36183476.

We conducted and reported this systematic review according to the PRISMA statement . The protocol was registered in the International Prospective Register of Systematic Reviews (PROSPERO) database (CRD42022311801).

1.1 Inclusion criteria and Exclusion criteria

RCTs that met the following criteria were included.

(1) Type of studies: RCTs investigating the efficacy and safety of Songling Xuemaikang Capsule for hypertension.

(2) Type of participants: Patients diagnosed with essential hypertension.

(3) Type of interventions: The experiment group was treated with Songling Xuemaikang Capsule or a combination with conventional antihypertensive medicines. The effects were compared to those observed in the placebo, conventional treatment, or no treatment groups.

(4) Type of outcomes: The primary outcomes were BP parameters, including systolic blood pressure(SBP), diastolic blood pressure(DBP), and 24-hour AMBP (24-h AMBP). Safety was measured as the occurrence of AEs.

Studies that met any of the following criteria were excluded.

(1) Studies that did not report blood pressure. (2) Crucial baseline data of participants were incomplete. (3) Interventions were not reported clearly.

1.2 Search strategy

Two independent reviewers conducted a systematic literature search for publications evaluating the efficacy and safety of Songling Xuemaikang Capsule for hypertension in seven electronic databases, including PubMed, Embase, the Cochrane Library, the Chinese National Knowledge Infrastructure (CNKI), Chinese Science and Technology Journals Database (VIP), Chinese Biomedical Literature Database (CBM), and the Wanfang Database, from these database built to March 21, 2022, without language limitations. The following search terms were used to identify potential articles containing MeSH (medical subject headings) and nonMeSH terms: ("Hypertension" OR "Hypertensions" OR "Blood Pressure, High" OR "Blood Pressures, High" OR "High Blood Pressure" OR "High Blood Pressures") and ("Songlingxuemaikang" OR "Songling Xuemaikang" OR "Song Ling Xue Mai Kang").

Additionally, we manually searched for references cited in similar previously published systematic reviews to ensure that no relevant studies were omitted.

1.3 Adverse events

19 comparative trials of Songling Xuemaikang Capsule combined with antihypertensive drugs and antihypertensive drugs reported adverse events, 6 of which had no adverse events in either experiment group or control group. The remaining 13 trials showed no significant difference in the rate of AEs between the two groups (OR = 0.79; 95% CI: 0.50, 1.27; *p* = 0.33), with no heterogeneity (Cochrane Q test = 5.14, p = 0.95, I^2^ = 0%) .

In the comparative trials of Songling Xuemaikang Capsule monotherapy and antihypertensive drugs, 3 trials reported adverse events. Meta-analysis revealed that both groups were well tolerated by patients with hypertension (OR = 0.31; 95% CI: 0.05, 1.82; *p* = 0.20), with significant heterogeneity (Cochrane Q test = 4.40, *p* = 0.11, I^2^ = 55%) .

The most common AEs included gastrointestinal symptoms, palpitation, skin rash, fatigue, and facial flushing. One trial reported four serious adverse events (SAEs), including acute appendicitis, lacunar cerebral infarction, numbness, and pneumonia, with two SAEs in the Songling Xuemaikang Capsule group and two in the groups.

**2.Qiangli Dingxuan Tablet**

Lin J, Wang Q, Zhong D, Zhang J, Yuan T, Wu H, Li B, Li S, Xie X, An D, Deng Y, Xian S, Xiong X, Yao K. Efficacy and safety of Qiangli Dingxuan tablet combined with amlodipine besylate for essential hypertension: a randomized, double-blind, placebo-controlled, parallel-group, multicenter trial. Front Pharmacol. 2023 Jul 10;14:1225529. doi: 10.3389/fphar.2023.1225529.

This was a randomized, double-blind, placebo-controlled, parallel-group, multicenter trial, which was done at 5 centers throughout China between March 2021 and June 2022. The trial protocol was approved by the Ethics Committee of Guang ‘anmen Hospital, China Academy of Chinese Medical Sciences (approval number: 2021-008-KY). The trial complied with the Declaration of Helsinki and was registered in ClinicalTrials.gov (Unique identifier: NCT05521282). The reporting in this article follows the Consolidated Standards of Reporting Trials. All participants were required to sign an informed consent form. After confirming that the participants provided informed consent and met the inclusion and exclusion criteria, a random number was assigned to the participants using a randomized system. This study had a

double-blind design, and the participants, investigators, and anyone involved in the analysis or interested in the trial were unaware of the trial drug class. Drugs can only be distinguished by drug number (random code).

2.1 Participants

The inclusion criteria: Men and women aged 18–75 years,diagnosed with grade 1 to 2 essential hypertension (SBP,140–179 mmHg and DBP, 90–109 mmHg) and with TCM syndrome diagnosis of hyperactivity of liver yang (The main TCM symptoms of the patients include vertigo, headache, and impetuosity, and the secondary symptoms include the red face,red eyes, dry mouth, bitterness in the mouth, constipation, etc).

Hypertension was diagnosed according to the 2018 Chinese guidelines for the management of hypertension . TCM syndrome diagnosis standard according to the

instructional principle of the latest Chinese herbal medicine to clinical research .

The Exclusion criteria: (1)Patients with secondary hypertension; (2)Patients with severe cardiovascular conditions such as coronary atherosclerotic heart disease, acute exacerbation of chronic heart failure, malignant arrhythmia, valvular heart disease,

cardiomyopathy, and other significant cardiovascular disorders; (3)Patients with acute cerebrovascular diseases including cerebral infarction and cerebral hemorrhage; (4)Patients with severe psychological disorders, intellectual disabilities, or language

impairments that hinder full cooperation with the study or completion of the study; (5)Patients with known allergies to Qiangli Dingxuan Tablet, excipients, or similar ingredients of the trial drug; (6)Patients with suspected or documented history of alcohol or drug abuse; (7)Pregnant or lactating women, or those who have recently planned or are unwilling to use contraceptive measures.

2.2 Safety evaluation

Investigators were responsible for recording all adverse events (AE) that occurred during the study. The duration, severity, and causal relationship of each AE to the study drug should be assessed, and the cause, as well as the actions and results taken to address the AE, should be documented. Clinical laboratory safety assessment

included blood routine tests, liver function tests, kidney function tests, and urine routine tests.

2.3 Results about safety

The AE was presented in Table 1. Based on the SAS, the Qiangli Dingxuan Tablet group has 8 patients (8 cases, including 1 case of the gastrointestinal system, 3 cases of the urinary system, 3 cases of the endocrine system, and 1 case of other systems) with AE occurred, and the placebo group has 9 patients (13 cases, including 8 cases of the blood system, 1 case of liver system, 1 case of the gastrointestinal system, 1 case of the urinary system, and 2 cases the endocrine system) . Relevance judgment through AE and test drugs, the Qiangli Dingxuan Tablet group has 5 patients (5 cases, including 1 case of the gastrointestinal system, 1 case of the urinary system, 2 cases of the endocrine system, and 1 case of other systems) with adverse events. The placebo group has 3 patients (3 cases, including 2 cases of the blood system, and 1 case of the gastrointestinal system) with adverse events. There was no significant difference in the incidence of AE and adverse events between the two groups (p>0.05). No significant differences were observed in blood routine test, urine routine test, liver function, and renal function (p > 0.05) (Table 2).

|  | Qiangli Dingxuan Tablet group | Placebo group | P value |
| --- | --- | --- | --- |
| All adverse events | 8 (6.02) | 9 (6.67) | 0.8268 |
| The blood system | 0 (0.00) | 8 (5.93) | 0.0070 |
| The liver system | 0 (0.00) | 1 (0.74) | 1 |
| The gastrointestinal system | 1 (0.75) | 1 (0.74) | 1 |
| The urinary system | 3 (2.26) | 1 (0.74) | 0.3685 |
| The endocrine system | 3 (2.26) | 2 (1.48) | 0.6828 |
| Other systems | 1 (0.75) | 0 (0.00) | 0.4963 |

Table 1 Adverse events reported during this study, n (%) (analysis based on safety analysis set)DT Placebo group vTable 2 Laboratory parameters (analysis based on safety set).

| Outcomes | Visit | Qiangli Dingxuan Tablet group | | Placebo group | | *P* value |
| --- | --- | --- | --- | --- | --- | --- |
|  |  | n (missing) | Mean (SD)/ n (%) | n (missing) | Mean (SD)/ n (%) |  |
| Blood routine test | | | | | | |
| HGB, g/L | Baseline | 124(9) | 147.66 (15.19) | 127(8) | 148.78 (13.65) | 0.4893 |
|  | 12 weeks | 90(43) | 146.50 (15.55) | 94(41) | 144.45 (15.35) | 0.4938 |
| RBC, 10^12^/L | Baseline | 124(9) | 4.85 (0.43) | 127(8) | 4.84 (0.46) | 0.6325 |
|  | 12 weeks | 90(43) | 4.73 (0.60) | 94(41) | 4.66 (0.57) | 0.3387 |
| WBC, 10^9^/L | Baseline | 124(9) | 6.60 (1.68) | 127(8) | 6.72 (2.03) | 0.8503 |
|  | 12 weeks | 90(43) | 6.70 (1.52) | 94(41) | 6.86 (1.99) | 0.9569 |
| NEUT, 10^9^/L | Baseline | 124(9) | 3.90 (1.28) | 127(8) | 3.71 (1.15) | 0.1334 |
|  | 12 weeks | 90(43) | 4.01 (1.62) | 94(41) | 4.13 (1.85) | 0.9901 |
| LY, % | Baseline | 124(9) | 33.41 (8.03) | 127(8) | 35.37 (8.06) | 0.0547 |
|  | 12 weeks | 90(43) | 33.97 (7.56) | 94(41) | 35.39 (9.41) | 0.3586 |
| PLT, 10^9^/L | Baseline | 124(9) | 240.79 (75.55) | 127(8) | 245.89 (60.92) | 0.4630 |
|  | 12 weeks | 90(43) | 240.42 (71.47 | 94(41) | 243.99 (61.90) | 0.8713 |
| Liver function test | | | | | | |
| ALT, U/L | Baseline | 126(7) | 23.57 (9.24) | 132(3) | 24.12 (11.88) | 0.6426 |
|  | 12 weeks | 90(43) | 20.94 (9.32) | 94(41) | 20.31 (8.98) | 0.4914 |
| AST, U/L | Baseline | 128(5) | 22.11 (5.70) | 134(1) | 22.16 (6.76) | 0.5692 |
|  | 12 weeks | 90(43) | 20.58 (5.02) | 94(41) | 21.31 (5.62) | 0.5360 |
| TBIL, μmol/L | Baseline | 126(7) | 14.78 (4.49) | 129(6) | 14.97 (5.48) | 0.6200 |
|  | 12 weeks | 90(43) | 14.12 (4.66) | 94(41) | 14.53 (4.79) | 0.3055 |
| DBIL, μmol/L | Baseline | 125(8) | 2.53 (1.01) | 128(7) | 2.62 (1.18) | 0.7193 |
|  | 12 weeks | 90(43) | 2.56 (0.89) | 94(41) | 2.81 (1.36) | 0.2231 |
| TP, g/L | Baseline | 127(6) | 72.86 (3.93) | 129(6) | 73.17 (4.86) | 0.6509 |
|  | 12 weeks | 90(43) | 72.01 (7.32) | 94(41) | 72.38 (6.16) | 0.7765 |
| ALP, U/L | Baseline | 117(16) | 78.36 (23.31) | 117(18) | 80.97 (21.14) | 0.1887 |
|  | 12 weeks | 90(43) | 79.17 (21.94) | 93(42) | 80.28 (22.20) | 0.6612 |
| Kidney function test | | | | | | |
| BUN, mmol/L | Baseline | 131(2) | 5.24 (1.17) | 127(8) | 5.25 (2.11) | 0.2962 |
|  | 12 weeks | 90(43) | 5.11 (1.42) | 94(41) | 5.03 (1.36) | 0.6388 |
| Scr, μmol/L | Baseline | 131(2) | 66.05 (14.63) | 129(6) | 65.92 (13.68) | 0.9205 |
|  | 12 weeks | 90(43) | 67.47 (28.09) | 94(41) | 64.91 (14.45) | 0.7722 |
| BUA, μmol/L | Baseline | 131(2) | 347.55 (88.05) | 129(6) | 350.44 (82.23) | 0.6752 |
|  | 12 weeks | 90(43) | 341.47 (89.29) | 94(41) | 343.87 (90.10) | 0.9713 |
| Urine routine test | | | | | | |
| Urine protein, ≥（+） | Baseline | 127(6) | 6(4.72) | 127(8) | 7(5.51) | 0.6324 |
|  | 12 weeks | 123(10) | 2(1.62) | 111(24) | 4(3.60) | 0.3231 |
| Urine glucose, ≥（+） | Baseline | 127(6) | 2(1.57) | 127(8) | 1(0.79) | 0.6399 |
|  | 12 weeks | 123(10) | 2(1.62) | 111(24) | 3(2.70) | 0.2972 |
| Urine erythrocyte, ≥（+） | Baseline | 127(6) | 18(14.17) | 127(8) | 19(14.96) | 0.5954 |
|  | 12 weeks | 123(10) | 8(6.50) | 111(24) | 10(9.01) | 0.4084 |
| Urine leukocyte, ≥（+） | Baseline | 127(6) | 15(11.81) | 127(8) | 15(11.81) | 0.5907 |
|  | 12 weeks | 123(10) | 8(6.50) | 111(24) | 11(9.91) | 0.3521 |

HGB, hemoglobin; RBC, red blood cell count; WBC, white blood cell count; PLT, blood platelet count; NEUT, neutrophil count; LY, lymphocyte; ALT, glutamic pyruvic transaminase; AST, glutamic oxalacetic transaminase; TBIL, total bilirubin; DBIL, direct bilirubin; TP, total protein; ALP, alkaline phosphatase; BUN, blood urea nitrogen; Scr, serum creatinine; BUA, blood uric acid.

**3.Tianma Gouteng Granule**

LIU Menglin，FAN Genhao，ZHANG Huailiang.Systematic evaluation and trial sequential analysis of Tianma Gouteng Granules combined with anti-hypertensive drugs in treatment of essential hypertension[J].China Journal of Chinese Materia Medica,2021,46(06):1511-1522.DOI:10.19540/j.cnki.cjcmm.20200702.501.

To systematically evaluate the efficacy and safety of Tianma Gouteng Granules combined with conventional antihypertensive drugs in the treatment of essential hypertension．

3.1 Literature source

The clinical randomized controlled trials (RCTs) on the treatment of essential hypertension with Tianma Gouteng Granules combined with conventional antihypertensive drugs were searched in PubMed，EMbase，Cochrane Library，VIP，CNKI，Wanfang，SinoMed since the establishment of the databases to April 2020 ，and meta-analysis was conducted by using RevMan 5. 3．

3.2 Inclusion Criteria

Study type: Clinical randomized controlled trial of Tianma Gouteng Granules in the treatment of essential hypertension.

Subjects: According to 《Chinese Guidelines for the Prevention and Treatment of Hypertension》, patients with SBP≥140mm Hg and/or DBP≥90 mmHg in the resting, non-pharmacological state, and exclusion of secondary factors can be diagnosed as having hypertension, regardless of their age, gender, and history of smoking and alcohol consumption.

Intervention: The control group was treated with conventional antihypertensive drugs only, and the experiment group was treated with the same antihypertensive drugs as the control group, including calcium antagonists (CCB), angiotensin-converting enzyme inhibitors (ACEI), angiotensin II receptor antagonists (ARB), diuretics, and β-receptor blockers.

Observations: The primary outcome indicators included SBP, DBP, and the secondary outcome indicators included clinical efficacy, adverse events/events etc.

3.3 Exclusion Criteria

Conference papers; studies with missing data that cannot be utilized or with serious errors in the data; reviews and animal experiments, etc.; only one duplicate publication will be retained.

3.4 Results about safety

A total of 15 RCTs were included，involving a total of 1508 patients．

Adverse events were reported in 7 papers, of which 6 had adverse events and 1 had nothing recorded. The heterogeneity test showed a high degree of heterogeneity among the studies(P=0.02，I^2^ = 64%), therefore, meta-analysis was performed using a random effects model, and the results showed no statistically significant difference between the experiment group and the control group in the incidence of adverse events,as shown in Fig.1．No liver and kidney dysfunction occurred．Trial sequential analysis showed that the studies accumulatively included for clinical efficacy crossed the traditional threshold，further affirming its clinical efficacy．The clinical application of Tianma Gouteng Granules combined with conventional western medicine in the treatment of hypertension and accompanying symptoms has clear efficacy and certain safety，so it is recommended for clinical application．


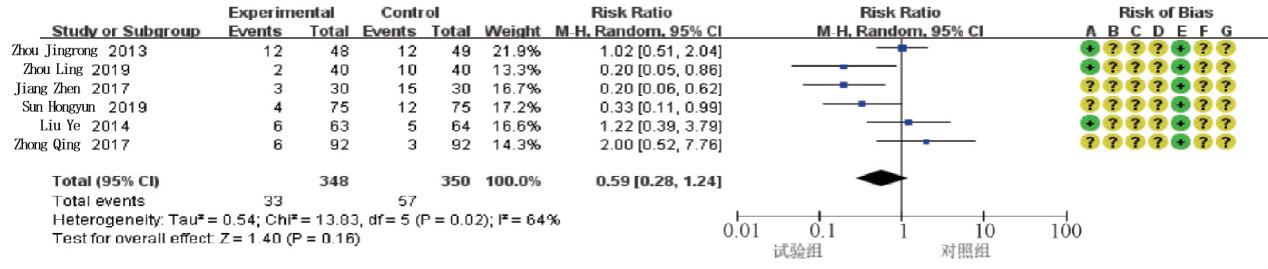


Fig.1 Forest plot of adverse event

**4.Qiju Dihuang Pill**

Guo Lei, Gao Jiankai. A randomized parallel controlled study of Qiju Di Huang Wan combined with nifedipine in the treatment of hypertension with liver and kidney yin deficiency[J]. Heilongjiang Medicine Journal,2019,32(04):818-820.DOI:10.140 3 5/j.cnki.hljyy.2019.04.027.

4.1 Data and Methods

General Data Study time: January 2016- January 2018; study object: 100 patients with liver and kidney Yin deficiency hypertension. Group method: 100 patients were randomly divided according to digital randomization into control group and experiment group consisting of 50 patients . The comparison of the general data between the two groupsis comparable,as shown in Table 3(P> 0.05).

Table 3 Comparison of the two groups of general data

| Group | n | age | male | female | disease course(year) |
| --- | --- | --- | --- | --- | --- |
| Experiment group | 50 | 62.71 ± 10.42 | 21 | 29 | 3.18 ± 1.79 |
| Control group | 50 | 62.09 ± 10.51 | 22 | 28 | 3.05 ± 1.84 |

4.2 Inclusion criteria

(1) met the diagnostic criteria of liver and kidney yin deficiency hypertension; (2) and not taking antihypertensive drugs recently; (3) informed about the study and actively cooperated.

4.3 Exclusion criteria

(1) pregnancy and lactation patients; (2) drug allergy; (3) patients not cooperating with the study.

4.4 Compare adverse events between two groups

Comparing the incidence of adverse events between the two groups, the difference between the two groups was not statistically significant (P＞0.05), of which one case of nausea and vomiting, one case of rash occurred in the control group, and the total incidence rate of adverse events was 4.00%.

In the control group, one case of nausea and vomiting, one case of skin rash occurred, and the total incidence rate of adverse events was 4.00%. In the experiment group, 2 cases of abdominal distension and 1 case of nausea occurred, and the total incidence rate of adverse events was 6.00%.

**5.Qinggan Jiangya Capsule**

Yuan Hui,Zhang Dawu,Zheng Yuan etc. Efficacy and Safety of Qinggan Jiangya Capsules in the Treatment of Essential Hypertension: a Meta-analysis[J].Chinese Journal of Integrative Medicine on Cardio-Cerebrovascular Disease, 2021, 19(20): 3455-3460.

5.1 Inclusion criteria

Study type: RCTs. The study subjects were clearly diagnosed as patients with essential hypertension.

Interventions: the control group gave the recommended (including diuretics, ACEI, ARB, β blockers, CCB and other drugs) or placebo combined with western medicine; the experiment group was combined with Qinggan Jiangya Capsule on the basis of the control group. Outcomes: ①clinical efficacy; ②SBP; ③DBP;④ safety evaluation, such as adverse drug reactions and adverse events.

5.2 Exclusion criteria

Did not mention specific course of treatment; interventions were mixed: other traditional Chinese medicines(TCM), acupuncture and other TCM treatments; no valid data or missing data; the article was published repeatedly.

5.3 Literature Search and Screening

Search CBM, Wanfang Database (Wanfang), CNKI, VIP, EMbase, PubMed, and Cochrane Library. Search from the database establishment until November 1,2020. Chinese search terms: Qinggan Jiangya Capsule, essential hypertension, hypertension; English search terms: Qinggan Jiangya, Qinggan Jiangya capsule, hypertension, highbloodpres-sure, essentialhypertension, highbloodpressures. All retrieved documents were screened repeatedly using NoteExpress software, and two researchers read the title, abstract and full text again. When the disagreement occurred, they were negotiated with the third researcher and relevant data were extracted.

5.4 Methodological quality evaluation

Evaluates random sequence generation, allocation concealment, blindness, outcome data integrity, selective reporting study, other bias, and makes high risk, low risk and uncertain risk judgment.

5.5 Statistic analysis

The meta-analysis of the data was performed using RevMan5.3 software. Effect size selection relative hazard for dichotomy data (relativerisk, RR), effect size selection mean variance for continuous data (meandifference, MD), quantitative data were performed using the normalized mean variance (standardized mean difference, SMD). All data with 95% confidence intervals (95%CI). Heterogeneity analysis was conducted by I^2^ test, I^2^ <30%, fixed-effect model for meta analysis; 30%≤I^2^<75%, ，eta analysis using random effects model; I^2^＞75%, sensitivity analysis and subgroup analysis. This meta-analysis used a funnel plot for publication bias analysis for＞10 literatures included in the literature.

5.6 Results about adverse events

A total of 5 studies reported adverse events, one study reported a case of transaminase elevation in the experiment group, 1 case of distal limb edema, dizziness, tachycardia, cough, control dizziness, mild nausea, tachycardia and cough; 1 study reported no adverse effects in groups, 2 cases of headache, 2 cases of face flushing; one study reported 2 cases of mild dry cough, dizziness and headache in the experiment group, 4 group of mild dry cough, head, halo and headache; one study reported 2 cases of mild ankle swelling and 1 case of dizziness and redness in both experiment group and control group; one study reported no adverse effects in the experiment group. In the control group, 1 case had facial flushing and 3 cases had headache. Meta-analyzed using a fixed-effect model( P=0.34, I^2^=11%). The results showed that the incidence of adverse events in the experiment group was lower than that in the control group with no statistically significant [RR=0.55,95%CI (0.27,1.13), P = 0.10]. See Figure 3 for details.


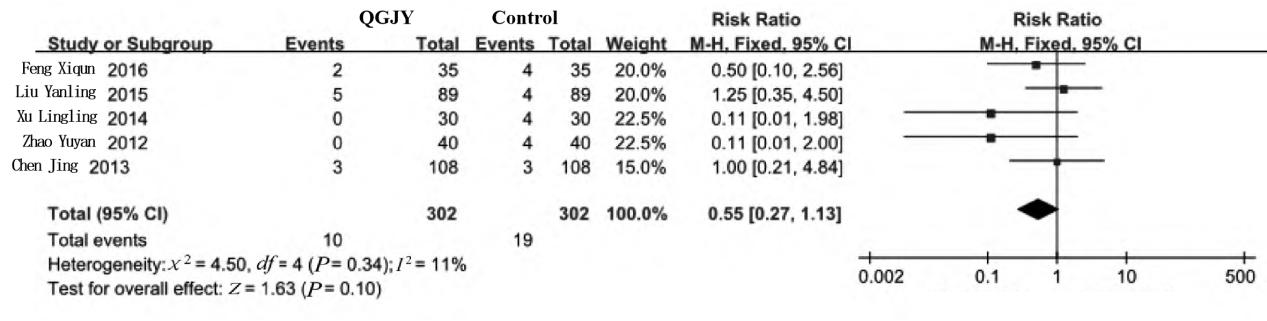


Fig.3 Forest plot for the meta-analysis of Adverse events

1. **Xinmaitong Capsule**

Yuan Huishu,Dai Fangguo,Geng Naizhi etc.Evaluation of a randomized controlled, double-blind, double-dummy and multicenter clinical trial on the efficacy and safety of Xinmaitong Capsule in the treatment of mild and moderate essential hypertension (blood stasis with Yin deficiency and Yang hyperactivity syndrome)[J]. China Medicine and Pharmacy,2023,13(03):17-20.

6.1 Data and Methods

Using randomized, double blind, double simulation, multicenter clinical trial methods. Choose 235 patients with mild and moderate essential hypertension (blood stasis block and Yin deficiency Yang hyperactivity syndrome) from 6 hospitals from December 30,2016 to April 18,2020 for the study object. Random number tables were used to divide into two groups. There was no statistically significant (P> 0.05) and were comparable, as shown in Table 4. This study was approved by the medical ethics of the Second Affiliated Hospital of Heilongjiang University of Traditional Chinese Medicine (Ethics approval number: Lun [2016] No.67, Second Hospital of Traditional Chinese Medicine).

Table 4 Comparison of the two groups of general data

| Group | n | breathing rate/min | pulse/min | SBP(mmHg) | DBP(mmHg) |
| --- | --- | --- | --- | --- | --- |
| experiment group | 176 | 18.1±2.2 | 71.9±9.7 | 153.0±10.0 | 94.3±10.9 |
| Control group | 59 | 18.5±2.4 | 69.9±8.0 | 151.1±8.8 | 94.1±7.9 |
| t |  | 1.181 | 1.429 | 1.300 | 0.130 |
| *p* |  | 0.890 | 0.171 | 0.193 | 0.895 |

6.2 Inclusion criteria

①met the diagnostic criteria for essential hypertension, both mild and moderate; ②met TCM syndrome differentiation; ③was stable and capable of communication and communication; the ④sitting position, the systolic blood pressure 140 mmHg and / or diastolic blood pressure 90 mmHg, and the blood pressure was lower than 180 / 110 mmHg.

6.3 Exclusion criteria

①Secondary hypertension caused by aortic coarctation, pheochromocytoma, etc.; ②grade 3 hypertension or with complications of hypertension;③pregnant, lactating women or drug allergy.

6.4 Methods

Control group: Xinkeshu tablets (produced by Shandong Huahua Pharmaceutical Technology Co., LTD. Batch number: 0160563, valid until April 2019; Batch number: 0181240, valid until November 2021, specification: 0.31 g / tablet) ,4 tablets / times, oral, 3 times / d, taken after meals, continuous medication for 12 weeks.

Experiment group: Xinmotong Capsule (produced by Guizhou Yibai Pharmaceutical Co., LTD. Batch number: 160701, valid until June 2018; Batch number: 180518, valid until April 2020, specification: 0.48g/grain) 3 times, oral, 3 times / d, continuous medication for 12 weeks.

6.5 Statistic analysis

Using SPSS 26.0 statistical software, measurement data are expressed as mean ±standard deviation (‾x ± s), t-test, count data are expressed as [n (%)], **χ2** test, P <0.05 is statistically significant.

6.5 Results about safety

The total incidence of adverse events in the experiment group was 7.39% (13 / 176), and the total incidence in the control group was 8.47% (5 / 59) in the control group. The total incidence rate between the two groups (**χ2**=1.261, P=0.780). The main manifestations are mild urinary department infection, cold, mild dyslipidemia and so on. It is suggested that the drug safety of patients with mild and moderate essential hypertension is high, and most patients can benefit from it.
