# Supplementary material for: Chinese patent medicine combined with calcium channel blockers in the treatment of essential hypertension:a Bayes network meta-analysis and systematic review
Source: Front Pharmacol. 2024 Mar 15;15:1321405. doi: 10.3389/fphar.2024.1321405 (PMC10978809; doi:10.3389/fphar.2024.1321405)
Supplement: Supplementary file 3 [file Table5.DOCX]

**The chemical composition names of Chinese patent medicine**

**(Latin names and associated families)**

| **Chinese patent medicine** | **Herb Chinese name** | **Herb Latin name** | **Family** |
| --- | --- | --- | --- |
| Songling Xuemaikang Capsule | 松叶 | Pinea Wolf | Pine genus |
|  | 葛根 | Pueraria lobata (Willd.) Ohwi | Fabaceae |
|  | 珍珠层粉 | Pteria martensii(Dunker) | Pearl oysters |
| Qiangli Dingxuan Tablet | 天麻 | Gastrodia elata Blume | Orchidaceae |
|  | 杜仲 | Eucommia ulmoides Oliv. | Eucommiaceae |
|  | 野菊花 | Chrysanthemum indicum L. | Asteraceae |
|  | 杜仲叶 | Eucommia ulmoides Oliv. | Orchidaceae |
|  | 川芎 | Ligusticum striatum DC. | Apiaceae |
| Tianma Gouteng Granule | 天麻 | *Gastrodia elata* Bl. | Orchidaceae |
|  | 钩藤 | *Uncaria rhynchophylla* Miq. | Rubiaceae |
|  | 石决明 | Haliotis diversicolor Reeve | Haliotis |
|  | 栀子 | *Gardenia jasminoides* J.Ellis | Rubiaceae |
|  | 黄芩 | Scutellaria baicalensis Georgi | Lamiaceae |
|  | 牛膝 | Achyranthes bidentata Bl. | Amaranthaceae |
|  | 杜仲 | *Eucommia ulmoides* Oliv. | Eucommiaceae |
|  | 益母草 | Leonurus japonicus Houtt. | Lamiaceae |
|  | 桑寄生 | Taxillus chinensis(DC.)Danser | Loranthaceae |
|  | 首乌藤 | *Polygonum muliflorum* Thunb. | Polygonaceae |
|  | 茯苓 | *Poria cocos*(Schw.)Wolf | Polyporeaceae |
| Qiju Dihuang Pill | 枸杞子 | *Lycium barbarum* L. | Solanaceae |
|  | 菊花 | *Chrysanthemum morifolium* (Ramat.) Hemsl. | Asteraceae |
|  | 熟地黄 | *Rehmannia glutinosa* (Gaertn.) DC. | Orobanchaceae |
|  | 山茱萸 | Cornus officinalis Siebold & Zucc. | Cornaceae |
|  | 牡丹皮 | Paeonia suffruticosa Andrews | Paeoniaceae |
|  | 山药 | Dioscorea oppositifolia L. | Dioscoreaceae |
|  | 茯苓 | *Poria cocos*(Schw.)Wolf | Polyporeaceae |
|  | 泽泻 | *Alisma plantago-aquatica* L. | Alismataceae |
| Qinggan Jiangya Capsule | 何首乌 | *Polygonum muliflorum* Thunb. | Polygonaceae |
|  | 夏枯草 | *Prunella grandiflora* (L.) Turra | Lamiaceae |
|  | 槐花 | *Styphnolobium japonicum* (L.) Schott | Fabaceae |
|  | 桑寄生 | *Taxillus chinensis*(DC.)Danser | Loranthaceae |
|  | 丹参 | *Salvia miltiorrhiza* Bunge | Lamiaceae |
|  | 葛根 | *Pueraria lobata* (Willd.) Ohwi | Fabaceae |
|  | 泽泻 | *Alisma plantago-aquatica* L. | Alismataceae |
|  | 小蓟 | Cirsium setosum (Willd.) Besser ex M.Bieb. | Asteraceae |
|  | 远志 | *Polygala tenuifolia* Willd. | Polygalaceae |
|  | 川牛膝 | *Cyathula officinalis* K.C.Kuan | Amaranthaceae |
| Xinmaitong Capsule | 当归 | *Angelica sinensis* (Oliv.) Diels | Apiaceae |
|  | 丹参 | *Salvia miltiorrhiza* Bunge | Lamiaceae |
|  | 毛冬青 | *Ilex pubescen*s Hook. & Arn. | Ilex genus |
|  | 粉葛 | *Pueraria lobata* (Willd.) Ohwi | Fabaceae |
|  | 牛膝 | *Achyranthes bidentata* Blume | Amaranthaceae |
|  | 钩藤 | *Uncaria rhynchophylla* Miq. | Rubiaceae |
|  | 槐花 | *Sophora japonica* L. | Fabaceae |
|  | 三七 | *Panax notoginseng* (Burkill) F.H.Chen | Araliaceae |
|  | 决明子 | *Cassia tora var. obtusifolia* (L.) Haines | Fabaceae |
|  | 夏枯草 | *Prunella grandiflora* (L.) Turra | Lamiaceae |
