# Supplementary material for: Chinese patent medicine combined with calcium channel blockers in the treatment of essential hypertension:a Bayes network meta-analysis and systematic review
Source: Front Pharmacol. 2024 Mar 15;15:1321405. doi: 10.3389/fphar.2024.1321405 (PMC10978809; doi:10.3389/fphar.2024.1321405)
Supplement: Supplementary file 4 [file Table3.DOCX]

**Chemical components of Chinese patent medicine**

**(data from liquid chromatography-mass spectrometry in literature)**

1. **Identification of constituents in Songling Xuemaikang Capsule**

HAO Xiaofeng, ZHAO Manxi, XIAO Yunchuan etc.Serum Pharmacochemistry of Songling Xuemaikang Capsules: Based on UPLC-MS/MS[J].Modern Chinese Medicine,2022,24(06):1042-1051.DOI:10.13313/j.issn.1673-4890.20210809004.

Songling Xuemaikang Capsule(Chengdu Kanghong Pharmaceutical Group Co.,Ltd, Lot No.Z109600230,human daily dose at 3 tablets/three times).

100g Songling Xuemaikang Capsule was griybd to powder. Add 10 mL distilled water , and the extract was ultrasonicated for 10 min (600 W, 40 kHz), filter from 0.45 μm membrane filtration, take 10 μL of filtrate for UPLC-MS/MS analysis.

UPLC conditions are as follows: C_18_ RRHD column (150 mm×2.1 mm, 1.8 μm), and 0.1% formic acid in water-0.1% formic acid in acetonitrile as mobile phase. MS data were collected in both positive and negative modes. The column temperature was set at 30℃, and the flow rate was 0.20 mL/min. The mobile phase consisted of solvent A (0.1% formic acid in water) and solvent B (0.1% formic acid in acetonitrile). The gradient for plasma was set as follows: 0-3min, 5%B; 3-15 min,5%-10%B；15-25 min，10%-20%B；25-40 min，20%-40%B；40-45 min，40%-100%B；45-50 min，100%B；50-51 min，100%-5%B；51-60 min，5%B）.

Acquisition in both positive and negative ion modes. Acquisition in both positive and negative ion modes. Positive ion mode: spray voltage is 3.5 kV, sheath gas pressure is 15 arb, auxiliary gas pressure is 5 arb, purge gas pressure is 0 arb, capillary temperature is 275℃, capillary voltage is 10 V, and lens voltage is 80 V; negative ion mode: spray voltage is 5000 V, sheath gas pressure is 15 arb, auxiliary gas pressure is 5 arb, purge gas pressure is 0 arb, capillary temperature is 275℃, capillary voltage is 10 V, lens voltage is 100 V. The second-order mass spectra were collected in data-dependent mode, the three most responsive peaks in the full scan spectrum were used for second-order mass spectrometry analysis, the collision gas was helium, the collision energy was 35 eV, and the full scan range was m/z 200 to 800.

79 chemical components were preliminarily identified from Songling Xuemaikang Capsule, and the specific information is shown in Table1. The mass spectra in the positive and negative ion modes of rat administered plasma samples and blank plasma samples are shown in Figure 1~2.


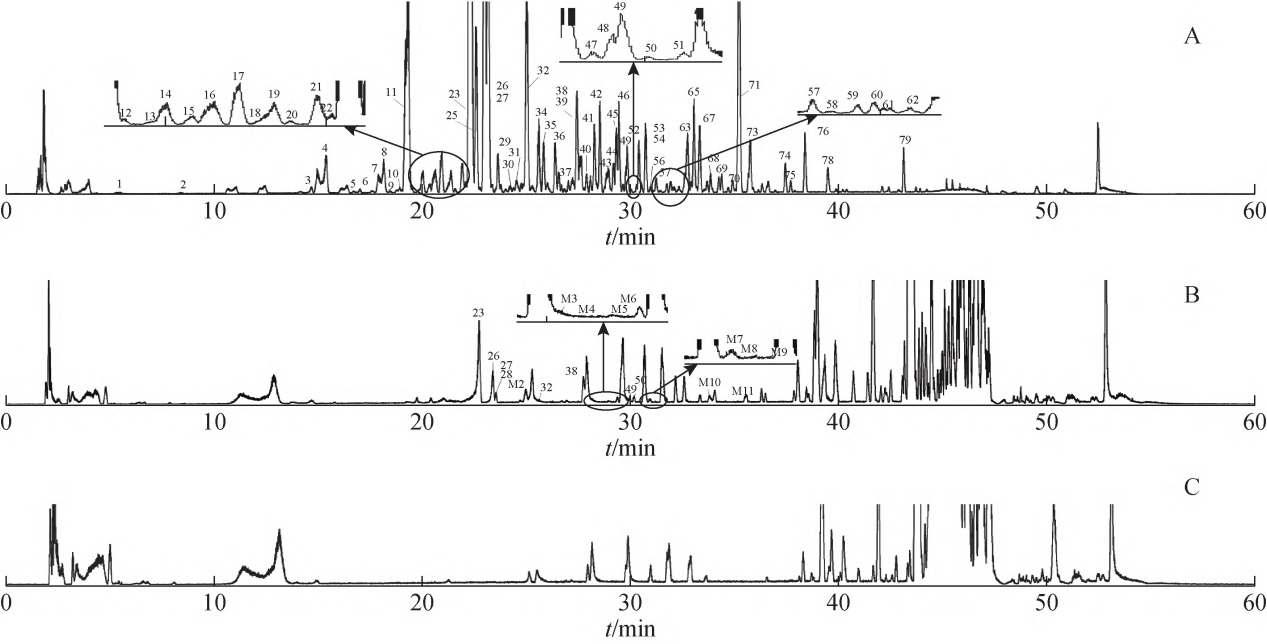


Fig. 1 Ion flow diagram in positive ion mode in the plasma of the pine age blood blood capsule, rats in the administration group and rats in the control group

Note: A. Songling Xiekang Capsule; B. Rat plasma of administration group; C. Rat plasma of control group; as in Figure 2.


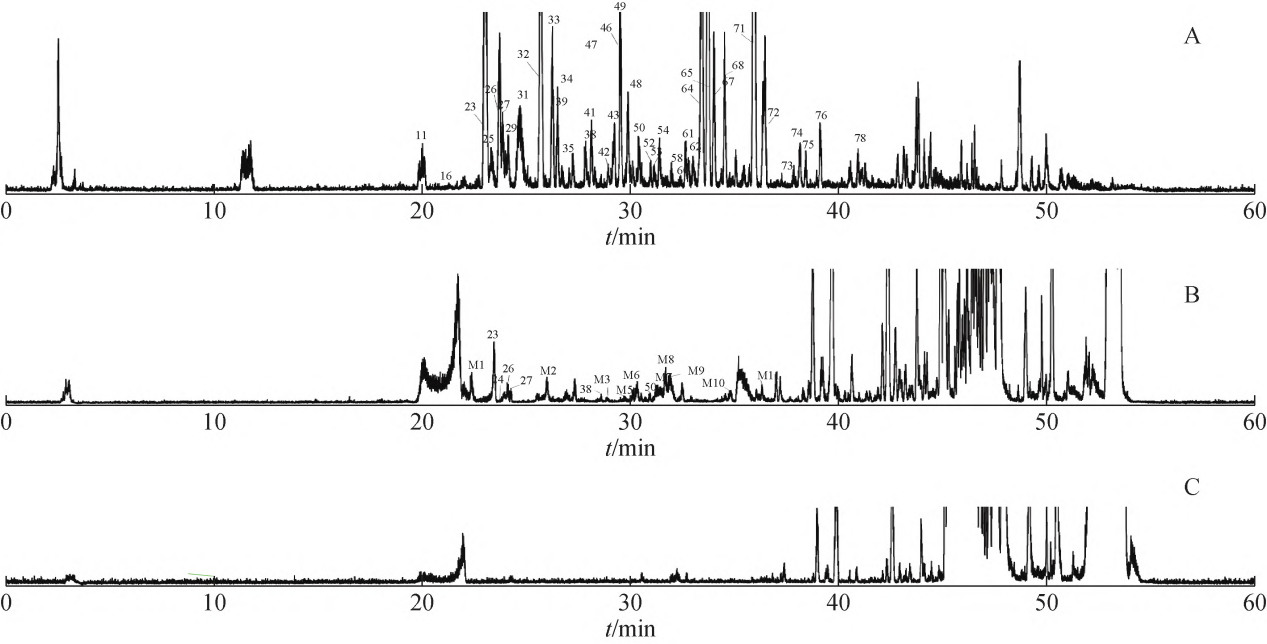


Fig. 2 Ion flow pattern in plasma anion mode of rats, plasma in the administration group and rats in the control group

Table 1. UPLC-MS/MS analysis of components of Songling Xuexue Kang Capsule

| Peak | Compound name | Source | tR/min | MS、MS2（m/z） | |
| --- | --- | --- | --- | --- | --- |
|  |  |  |  | Cationic mode | Anion mode |
| 1 | — | Fresh pine leaf | 4.97 | 399[M+H]^+^，381，351 |  |
| 2 | — | Fresh pine leaf | 7.73 | 518[M]^+^，339，147 |  |
| 3 | 3'-hydroxy-4'-O-glucosepuerarin | kudzu | 13.84 | 595[M+H]^+^，577，433 | 593[M-H]^–^，431，311 |
| 4 | Puerarin4'-O-glucoside | kudzu | 14.60 | 579[M+H]^+^，561，417，399 | 577[M-H]^–^，457 |
| 5 | 3'-methoxy-puerarin4'-O-glucoside | kudzu | 16.26 | 609[M+H]^+^，447 |  |
| 6 | Genistein-7-gentiobioside | kudzu | 16.48 | 595[M+H]^+^，433，271 |  |
| 7 | 4'-o-glucose-puerarinapigenin | kudzu | 17.21 | 711[M+H]^+^，579，561，417，399 | 709[M–H]^–^，457 |
| 8 | Daidzein4',7-diglucoside | kudzu | 17.41 | 579[M+H]^+^，417，255 | 623[M+HCOOH–H]^–^，415 |
| 9 | Catechin | Fresh pine leaf | 17.83 | 291[M+H]^+^，273 | 289[M–H]^–^ |
| 10 | Puerarin - 7-O-glucoside | kudzu | 18.21 | 579[M+H]^+^，417，399，381，351， 321，297 |  |
| 11 | 3'-hydroxypuerarin | kudzu | 18.47 | 433[M+H]^+^，415，397，367，313 | 431 [M–H]^–^，311 |
| 12 | 3'-methoxy-4' -o-glucose daidzein | kudzu | 18.97 | 609[M+H]^+^，447，285 | 653 [M+HCOOH–H]^–^，445 |
| 13 | 3'-hydroxy-4' -O-glucose puerarin | kudzu | 19.26 | 595[M+H]^+^ ，433，415 | 593 [M–H]^–^，473 |
| 14 | Genistein 8-c-celery glucoside | kudzu | 19.52 | 565[M+H]^+^ ，433，415，313 |  |
| 15 | 3'-hydroxy -4' -O-glucose-6"-o-cainose | Fresh pine leaf | 19.80 | 344 [M+NH_4_]^+^，327，165 |  |
| 16 | Puerarin | kudzu | 20.01 | 727 [M+H]^+^，709，433，595，565 | 725 [M–H]^–^，473 |
| 17 | 3'-hydroxy-6"-O-xylopuerarin | kudzu | 20.42 | 565 [M+H]^+^，433，415 | 563 [M–H]^–^，311，283 |
| 18 | 6"-O-glucose-puerarin | kudzu | 20.59 | 579 [M+H]^+^，561，417，399 |  |
| 19 | Genistein 4', 7-di-glucoside | kudzu | 20.96 | 595 [M+H]^+^，433，271 | 639 [M+HCOOH–H]^–^，431，593 |
| 20* | (6S,7E,9R)- Macrobiotin | Fresh pine leaf | 21.21 | 387 [M+H]^+^，207，369 | 431 [M+HCOOH–H]^–^，385 |
| 21 | Sophoricoside | kudzu | 21.37 | 433 [M+H]^+^，271 |  |
| 22 | Physosine-4 '-o-glucoside | kudzu | 21.52 | 727 [M+H]^+^，595，709，565，433，271 |  |
| 23* | Puerarin | kudzu | 21.70 | 417 [M+H]^+^，399，381，363，351，321，297 | 415 [M–H]^–^，295，267 |
| 24 | Genistein 8-C-glucoside | kudzu | 21.70 |  | 431 [M–H]^–^，311，283 |
| 25 | Puerarin xyloside | kudzu | 22.12 | 549 [M+H]^+^，417，399，381，363，351，321，297 | 547 [M–H]^–^，295，267 |
| 26 | 3'-methoxypuerarin | kudzu | 22.51 | 447 [M+H]^+^，429，411，393，381，351，327 | 445 [M–H]^–^，325，297 |
| 27 | Puerarin apigenin | kudzu | 22.70 | 549 [M+H]^+^，417，399，381，363，351，321，297 | 547 [M–H]^–^，295，267 |
| 28 | β-sitosterol | Fresh pine leaf、kudzu | 22.87 | 432 [M+NH_4_]^+^，415 |  |
| 29 | 3'-methoxy-6"-O-xylopuerarin | kudzu | 23.26 | 579 [M+H]^+^，447，429 | 577 [M–H]^–^，325 |
| 30 | (-) -hyacinthoside | kudzu | 23.70 | 549 [M+H]^+^，417，255 |  |
| 31 | 8-C-glucosyl puerarin | kudzu | 24.05 |  | 445 [M–H]^–^，325 |
| 32* | Daidzin | kudzu | 24.45 | 417 [M+H]^+^，255 | 461 [M+HCOOH–H]^–^，415，253 |
| 33* | Massonin B | Fresh pine leaf | 25.22 | 510 [M+NH_4_]^+^， 493， 475， 463，445，427，409，317 | 537 [M+HCOOH–H]^–^，491，345，315 |
| 34 | Daidzein | kudzu | 25.38 | 447 [M+H]^+^，285 | 491 [M+HCOOH–H]^–^，445，283 |
| 35 | Cycloisolicinol-9-O-glucoside | Fresh pine leaf | 25.70 | 542 [M+NH_4_]^+^，363，345，327 | 523 [M–H]^–^，361，331 |
| 36 | 3'-methoxy-6"-o-sericin | kudzu | 25.91 | 565 [M+H]^+^，433，415，397 |  |
| 37 | Conical sandaltine | kudzu | 26.56 | 463 [M+H]^+^，445，427，409，397，367，343 | 461 [M–H]^–^，341 |
| 38 | Daidzein 4 '-o-glucoside | kudzu | 26.86 | 417 [M+H]^+^，399，351，297，255 | 415 [M–H]^–^，295，253 |
| 39 | Genistein 4 '-(6 "-o-malonyl glucoside) | kudzu | 26.86 | 519 [M+H]^+^，271 |  |
| 40 | Isobarinin-9 '-O-glucoside | Fresh pine leaf | 27.28 | 540 [M+NH_4_]^+^，331，313 |  |
| 41 | Iris flavone-7-O-xylosyl-8-C-glucoside | kudzu | 27.72 | 595 [M+H]^+^，463，445，343 | 593 [M–H]^–^，341，326，298 |
| 42* | Isobarinin-9'-o-arabinoside | Fresh pine leaf | 27.99 |  | 491 [M–H]^–^，359，341，311 |
| 43 | Physogenin | kudzu | 27.99 | 565 [M+H]^+^，433，547，271 | 609 [M+HCOOH–H]^–^，563，269 |
| 44 | 6-C-methyl-vanillin - 7-O-glucoside | kudzu | 28.25 | 465 [M+H]^+^，303 | 463 [M–H]^–^，301 |
| 45 | Puerarin B | kudzu | 28.45 | 637 [M+H]^+^，619，475，313 | 681 [M+HCOOH–H]^–^ |
| 46 | genistein | kudzu | 28.64 | 433 [M+H]^+^，271 | 431 [M–H]^–^，311 |
| 47 | 4'-methoxy-6"-O-xylopuerarin | kudzu | 28.70 | 563 [M+H]^+^，431，413，311 | 561 [M–H]^–^，309，281 |
| 48 | cedrusin | Fresh pine leaf | 28.91 | 347 [M+H]^+^，329，311 | 345 [M–H]^–^ |
| 49 | 4'-methoxy-6"-o-sericin | kudzu | 29.15 | 563 [M+H]^+^，431，413，311 | 561 [M–H]^–^，309，281 |
| 50 | 4'-methoxypuerarin | kudzu | 29.46 | 431 [M+H]^+^，413，395，377，365，335，311 | 429 [M–H]^–^，309 |
| 51 | 8(7s,r)-4,9'-dihydroxy-3,3'-dimethoxy7,8-dihydro coumarone-1'-propyl new wooden fat element-9-O-glycosidase | Fresh pine leaf | 29.61 | 540 [M+NH_4_]^+^，331，493 | 521 [M–H]^–^，491 |
| 52 | 6"-O-malonyl-4'-methoxygenin | kudzu | 30.04 | 533 [M+H]^+^，285 | 283 [M–H–MalonylGlu]^–^，268 |
| 53 | Dihydrocerasin | kudzu | 30.22 | 449 [M+H]^+^，287 | 447 [M–H]^–^，285 |
| 54 | Sesquimarocanol A | kudzu | 30.51 |  | 601 [M+HCOOH–H]^–^，555 |
| 55 | — | kudzu | 30.77 | 507 [M+H]^+^，303 | 505 [M–H]^–^，463 |
| 56 | Yemuoside YM2 | kudzu | 30.77 | 688 [M+NH_4_]^+^，671，509 | 669 [M–H]^–^，507 |
| 57 | Naringenin-7-O-glucoside | kudzu | 30.86 |  | 433 [M–H]^–^，271 |
| 58 | 3'-hydroxyirisflavin | kudzu | 31.01 | 479 [M+H]^+^，317 | 477 [M–H]^–^，449，357，315 |
| 59 | (–)-massoniresinol | Fresh pine leaf | 31.17 | 393 [M+H]^+^，375 |  |
| 60 | 6"-o-acetyldaidzein | kudzu | 31.38 | 459 [M+H]^+^，255 | 503 [M+HCOOH–H]^–^，457，253 |
| 61 | 6"-o-acetyl-isoquercitrin | kudzu | 31.56 | 507 [M+H]^+^，303 | 505 [M–H]^–^ |
| 62 | glycyrrhizin | kudzu | 31.80 | 563 [M+H]^+^，431，269 | 607 [M+HCOOH–H]^–^，561，267 |
| 63 | Puerarin C | kudzu | 32.20 | 475 [M+H]^+^，313 |  |
| 64 | 6"-o-malonyl genistein | kudzu | 32.42 | 519 [M+H]^+^，271 | 269 [M–H–MalonylGlu]^–^ |
| 65 | 4',7-dihydroxy-3'-methoxyisoflavone 8-C apiosin-glucoside | kudzu | 32.86 | 579 [M+H]^+^，447，429，327 | 577 [M–H]^–^ |
| 66* | Massonin D | Fresh pine leaf | 32.94 | 524 [M+NH_4_]^+^，477，459，441 | 551 [M+HCOOH–H]^–^，505，341 |
| 67* | Ononin | kudzu | 32.99 | 431 [M+H]^+^，269，254 | 475 [M+HCOOH–H]^–^，267，252 |
| 68 | symplolignanoside A | Fresh pine leaf | 33.69 | 655 [M+H]^+^，493，331 | 653 [M–H]^–^，491 |
| 69 | isononin | kudzu | 33.97 | 431 [M+H]^+^，269 |  |
| 70 | 8-methoxyononidine | kudzu | 34.42 | 461 [M+H]^+^，299，28 |  |
| 71* | daidzein | kudzu | 34.63 | 255 [M+H]^+^，237 |  |
| 72 | 4',5, 7-trihydroxy-isoflavone-6-methylether-7-O-xylose-glucoside | kudzu | 35.39 | 579 [M+H]^+^，447，285 | 623 [M+HCOOH–H]^–^，577，283 |
| 73 | 6"-o-malonoside | kudzu | 35.97 | 517 [M+H]^+^，269，254 | 267 [M–H–MalonylGlu]^–^，252 |
| 74 | 3'-methoxydaidzein | kudzu | 37.12 | 447 [M+H]^+^，285 | 491 [M+HCOOH–H]^–^，283 |
| 75 | massonianoside C | Fresh pine leaf | 37.29 | 507 [M+H]^+^ | 505 [M–H]^–^ |
| 76 | Isobarinin-9-O-xyloside | kudzu | 38.12 | 493 [M+H]^+^，331 | 491 [M–H]^–^，329 |
| 77 | 6"-o-acetylononidine | kudzu | 38.34 | 473 [M+H]^+^，269 |  |
| 78 | 6"-o-malonyl-chickpea A | kudzu | 39.53 | 533 [M+H]^+^，285 | 283 [M–H–MalonylGlu]^–^，268 |
| 79 | Resinol-8-O-glucoside | Fresh pine leaf | 42.34 | 521 [M+H]^+^，359 |  |

1. **Identification of constituents in Qiangli Dingxuan Tablet**

Luo Huanhuan;Feng Zeyu, et al.;Simultaneous determination of thirteen constituents in Qiangli Dingxuan Tablets by HPLC[J].Chinese Traditional Patent Medicine,2022,44(01):23-27.

Qiangli Dingxuan Tablet (Shaanxi Hanwang Pharmaceutical Co., Ltd, Lot No. Z61020139, human daily dose at 4-6 tablets/three times).

Test solution: Take 10 tablets of this product, remove the film coating, grind fine, precise weighing about 0.5g. Add 50mL of 50% methanol and sonicate (250 W, 40 kHz) for 30 min.

Control solution: 1ml methanol with gastrodin 212. 50 μg, gallic acid 233. 00 μg,5-hydroxymethyl furfural 136. 50 μg;para-hydroxybenzyl alcohol 208. 00 μg, neochlorogenic acid 493. 00 μg, chlorogenic acid 224. 50 μg, cryptochlorogenic acid 227. 50 μg, parisin B 125. 00 μg, pinolinol diglucoside 122. 00 μg, parisin C 160. 50 μg, parisin A 154. 00 μg, Ligustilide 225. 00 μg, linarin 234 μg.

Negative sample solution preparation: According to the prescription of this product, respectively Deficiency of Tianma, Cortex Eucommiae, Cortex Eucommiae Leaf, Chrysanthemum and Rhizoma Ligustici Chuanxiong were obtained. Negative samples were prepared according to the method like “Test solution”.

UPLC conditions are as follows: Lamdo Stamsil C_18_ RRHD column (250 mm×4.6 mm, 5 μm). Mobile phase acetonitrile（A）-0.05% phosphoric acid（B）. The gradient for plasma was set as follows: 0-2min, 3%-7%A; 2-20 min, 7%A；20-30 min，7%-12%A；30-90 min，12%-30%A；90-100 min，30%-60%A）.The column temperature was set at 20℃, and the flow rate was 0.70 mL/min. Detection wavelength: 220, 280 nm; injection volume: 5 μL.


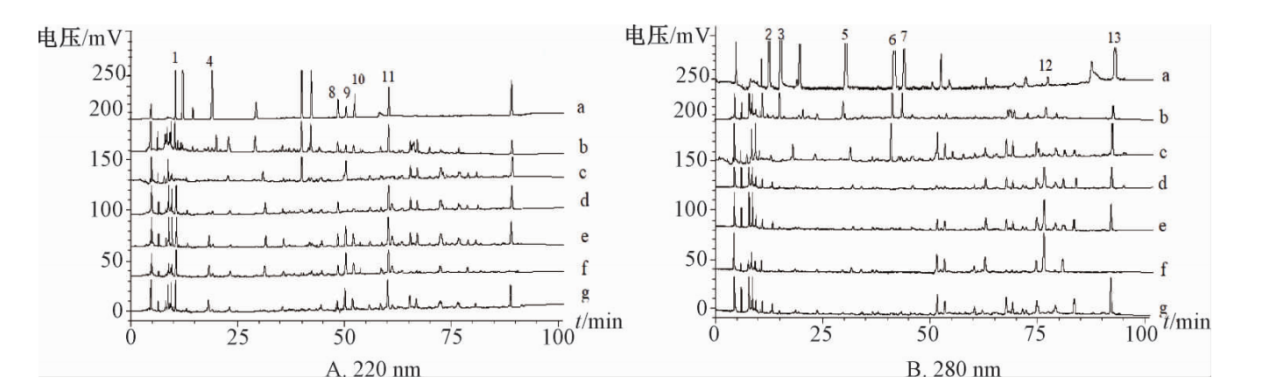


Fig. 3 HPLC chromatograms of various constituents

Note: a- g are control samples, test samples, *Gastrodia elata* Blume negative samples, *Eucommia ulmoides* Oliv. leaf of *Eucommia ulmoides* Oliv. negative samples, *Chrysanthemum indicum* L. negative samples, *Ligusticum striatum* DC. negative samples.

1.gastrodia elata ; 2. gallic acid; 3. 5-hydroxymethyl furfural; 4. para-hydroxybenzyl alcohol; 5．neochlorogenic acid; 6.chlorogenic acid; 7. cryptochlorogenic acid; 8．parisin B; 9．pinolinol diglucoside; 10．parisin C; 11．parisin A; 12．Ligustilide; 13. linarin

Table 2 Content determination

| Peak | Component Formula | Formula | Content (mg/g) |
| --- | --- | --- | --- |
| 1 | Gastrodin | C_13_H_18_O_7_ | 2.21±0.00 |
| 2 | Gallic acid | C_7_H_6_O_5_ | 1.38±0.08 |
| 3 | 5-Hydroxymethylfurfural | C_6_H_6_O_3_ | 0.38±0.09 |
| 4 | p-Hydroxybenzyl Alcohol | C_7_H_8_O_2_ | 0.39±0.08 |
| 5 | 5-Caffeoylquinic acid | C_16_H_18_O_9_ | 6.32±0.07 |
| 6 | Chlorogenic acid | C_16_H_18_O_9_ | 1.45±0.49 |
| 7 | 4-Dicaffeoylquinic Acid | C_16_H_18_O_9_ | 1.39±0.00 |
| 8 | Parishin **B** | C_32_H_40_O_19_ | 2.36±0.20 |
| 9 | Pinoresinol Diglucoside | C_32_H_42_O_16_ | 0.62±0.01 |
| 10 | [Parishin C](https://www.chemsrc.com/en/cas/174972-80-6_1198928.html) | C_32_H_40_O_19_ | 0.91±0.05 |
| 11 | [Parishin A](https://www.chemsrc.com/en/cas/174972-80-6_1198928.html) | C_45_H_56_O_25_ | 3.31±0.02 |
| 12 | Z-Ligustilide | C_12_H_14_O_2_ | 0.03±0.00 |
| 13 | Linarin | C_28_H_32_O_14_ | 1.30±0.03 |

1. **Identification of constituents in Tianma Gouteng Granule**

Zhang H, Wang L, Lu B, Qi W, Jiao F, Zhang H, Yuan D. Metabolite profiling and quantification of phytochemicals of Tianma-Gouteng granule in human and rat urine using ultra high performance liquid chromatography coupled with tandem mass spectrometry. J Sep Sci. 2019 Sep;42(17):2762-2770. doi: 10.1002/jssc.201900029

Tianma Gouteng Granule (Chengdu Jiuzhitang Jinding Pharmaceutical, Lot No. Z51021084, human daily dose at 15 g/three times)was purchased from Guoda Pharmacy Shenyang Branch Store (Shenyang, China). Fifteen reference compounds were used in this study.

Three male healthy Chinese volunteers (age range: from 20 to 25) fasted for 12 h (from 8:00 pm to 8:00 am) before the experiment. Blank urine samples were collected for 3 h before oral administration. The volunteers received a single oral administration of Tianma Gouteng Granule at dose of 5g (1 packet, dissolved in 100 mL water) according to instruction for taking Tianma Gouteng Granule. During the collection, 100 mL of drinking water per hour with three low-fat meals in a day after oral administration. Their urine samples were collected each 2h. After the volume of individual collected samples was measured and recorded, an aliquot of 1% by volume was taken from individual samples to make a pooled urine sample for 0–24 h. The urine sample was centrifuged at 3500 rpm for 10 min at 4℃, and the supernatant were then stored at −20℃ until additional extraction and analysis.

For metabolite identification, the chromatography separation was performed on a Waters ACQUITY HSS C18 column (2.1×100 mm, 1.8 µm, Waters, Milford, MA, USA)at 40^◦^C. The mobile phase consisted of (A) water containing 0.1% formic acid and (B) acetonitrile. The optimized elution conditions were as follows: 0~2.5 min, 5~18%(B); 2.5~3.5 min, 18~19% (B); 3.5~5.0 min, 19~20% (B);5~7 min, 20~22% (B); 7~10 min, 22~30% (B); 10~13 min,30~55% (B); 13~15 min, 55~99% (B); 15~15.1 min, 99~5%(B); 15.1~16.0 min, 5% (B). The flow rate was maintained at 0.4 mL/min, and 2 µL of sample solution was injected in each run.

Leucine-enkephalin was used as the lock mass to generate a [M+H]^+^ ion (m/z 556.2271),[M-H]^−^ ion (m/z 554.2615) in the LockSpray mode at a concentration of 50 pg/µL at an infusion flow rate of 10 µL/min. The ESI source was operated in both positive and negative ionization modes, with the capillary voltage being set at 2.5 and −2.0 kV, respectively. The cone voltage was set to 45 V. Source and desolvation temperatures were set at 130 and 450^◦^C, respectively. The cone and nebulization gas flows were 50 and 800 L/h, respectively. All data collected in centroid mode were acquired using MasslynxTM NT 4.1 software (Waters). The MS*^E^* experiment was carried out in two scan functions. Function 1, m/z 50–1500, 0.2 s scan time, and 6 eV collision energy, was to obtain the quasi-molecular ion and the elemental composition of the target compounds. Function 2, m/z 100–1200, 0.2 s scan time, and a collision energy ramp of 20–40 eV, was to acquire the MS fragmentation data of target compounds from a single run.

The MS/MS data was acquired in multiple reaction monitoring mode coupled with an ESI source in the positive and negative ion mode using an AB SCIEX API4000TM (AB SCIEX, USA). Seven transitions between the precursor ion of seven prototype components and their seven most abundant product ions were monitored for quantitative determination as follows, m/z 285.1 > 123.0 (GAS), m/z 387.1 > 225.2(GPS), m/z 445.1 > 269.2 (BCL), m/z 459.2 > 283.0 (WGS), m/z 269.1 > 225.0 (ED), m/z 312.2 > 181.2 (LEO), and m/z 385.6 > 160.3 (RIN). To increase sensitivity, the ion source temperature was set at 450◦C, and the positive and negative ion spray voltage was set at 5.5 and −4.5 kV, respectively. Ion source gas 1 and 2 used as the nebulizing and drying gases were set at 50 psi. Curtain gas was set at 25 psi.

**Table** **3** Compound-dependent parameters in UPLC-MS/MS analysis.

| Compounds | Q1 (m/z) | Q3 (m/z) | Dwell time (ms) | DP | CE | CXP |
| --- | --- | --- | --- | --- | --- | --- |
| GAS | 285.1 | 123.0 | 100.00 | -70 | -16 | -6 |
| GPS | 387.1 | 225.2 | 100.00 | -57 | -13.5 | -15 |
| GPS-Glu | 401.1 | 387.1 | 100.00 | -57 | -13.5 | -15 |
| BCL | 445.1 | 269.2 | 100.00 | -61 | -23.5 | -15 |
| BCLE | 269.0 | 195.1 | 100.00 | -110 | -35 | -11 |
| WGS | 459.2 | 283.0 | 100.00 | -60 | -22.5 | -15 |
| WGS-SO_3_ | 539.1 | 459.2 | 100.00 | -60 | -22.5 | -15 |
| WGSE-SO_3_ | 363.1 | 283.0 | 100.00 | -60 | -22.5 | -15 |
| demethyl-WGS | 445.1 | 283.0 | 100.00 | -60 | -22.5 | -15 |
| ED | 269.1 | 225.0 | 100.00 | -90 | -36 | -13 |
| RIN | 385.6 | 160.3 | 100.00 | 101 | 42.0 | 10 |
| IRN | 385.5 | 160.3 | 100.00 | 99 | 48 | 15 |
| LEO | 312.2 | 181.2 | 100.00 | 91 | 30 | 11 |
| LEO-Glu | 488.0 | 312.2 | 100.00 | 91 | 30 | 11 |
| IR (IS) | 675.4 | 513.2 | 100.00 | -117 | -9 | -16 |
| CBP (IS) | 237.2 | 194.3 | 100.00 | 90 | 24 | 12 |

**Table 4** The components and metabolites identified in human and rat urine after a single oral administration of TGG by using UPLC-Q-TOF-MS method.

| No. | t*_R_*  (min) | Formula | Positive mode (m/z) | | Negative mode (m/z) | | Identification | Source | Urine | |
| --- | --- | --- | --- | --- | --- | --- | --- | --- | --- | --- |
|  |  |  | [M+H]^+^ | MS/MS | [M-H]^-^ | MS/MS |  |  | Rat | Human |
|  |  |  | (error, ppm) |  | (error, ppm) |  |  |  |  |  |
| P1 | 1.15 | C_14_H_20_O_9_ | n.d. | n.d. | 331.1046(5.1)  [M-H+HCOOH]^-^ | 285,123 | gastrodin | G | + | + |
| P2 | 1.15 | C_16_H_24_O_11_ | n.d. | n.d. | 391.1234(-1.5) | 229,167 | shanzhiside | GF | + | - |
| P3 | 1.56 | C_16_H_22_O_10_ | n.d. | n.d. | 373.1115(-5.3) | 211,191,167 | geniposidic acid | E,GF | + | + |
| M1 | 1.69 | C_17_H_26_O_11_ | n.d. | n.d. | 451.1429(-6.2)  [M-H+HCOOH]^-^ | 243 | shanzhiside methyl ester | GF | + | - |
| P4 | 1.70 | C_15_H_20_O_10_ | n.d. | n.d. | 359.0966(-3.3) | 197 | glucosyringic acid | G | + | - |
| P5 | 1.75 | C_16_H_22_O_11_ | n.d. | n.d. | 403.1241(0.3) | 389,241 | deacetylasperulosidic acid methyl ester | GF | + | - |
| P6 | 1.88 | C_17_H_24_O_11_ | n.d. | n.d. | 403.1236(-1.0) | 241,223 | gardenoside | GF | + | - |
| M2 | 2.01 | C_26_H_30_O_15_ | n.d. | n.d. | 581.1484(3.9) | 405,243 | TSG-glucuronide | PM | + | - |
| P7 | 2.04 | C_19_H_24_O_13_ | n.d. | n.d. | 459.1134(-1.1) | 173,129 | parishin E | G | + | + |
| M3 | 2.41 | C_27_H_32_N_2_O_10_ | 545.2165(5.5) | 369,351 | n.d. | n.d. | demethyl-ICO-glucuronide | UR | + | + |
| M4 | 2.44 | C_14_H_21_N_3_O_5_ | 488.1893(2.2） | 312,198 | n.d | n.d | leonurine-10-O-β-D-glucuronide | L | + | + |
| P8 | 2.51 | C_23_H_34_O_15_ | n.d. | n.d. | 549.1823(0.7) | 225 | genipin gentobioside | GF | + | + |
| M5 | 2.69 | C_15_H_20_O_8_ | n.d. | n.d. | 327.1088(2.4) | 285,123 | acetyl-gastrodin | G | + | - |
| M6 | 2.70 | C_27_H_34_N_2_O_10_ | 547.2297(0.9) | 371,353 | n.d. | n.d. | demethyl-IRN-glucuronide | UR | + | + |
| M7 | 2.72 | C_11_H_14_O_8_S | n.d | n.d | 305.0364(5.90) | 273,225 | genipin-1-O-sulfate | GF,E | + | - |
| M8 | 2.73 | C_16_H_12_O_5_ | n.d. | n.d. | 123.0436(-8.1) | 105 | p-hydroxybenzyl alcohol | G | + | + |
| M9 | 2.88 | C_26_H_30_O_15_ | n.d. | n.d. | 581.1476(-5.1) | 405,243 | TSG-glucuronide | PM | + | + |
| M10 | 2.93 | C_17_H_22_O_11_ | n.d. | n.d. | 401.1118(-3.2) | 225,176 | genipin-1-O-glucuronide | GF,E | + | + |
| P9 | 2.95 | C_32_H_40_O_19_ | n.d. | n.d. | 727.2078(-1.1) | 459,441,423 | parishin B | G | + | + |
| P10 | 2.96 | C_17_H_24_O_10_ | n.d. | n.d. | 433.1326(-4.6)  [M-H+HCOOH]^-^ | 387,225,207 | geniposide | GF,E | + | + |
| M11 | 2.98 | C_27_H_26_O_17_ | 623.1250(0.3) | 447,271 | 621.1072(-3.2) | 445,269 | 5,6,7-trihydroxyflavone-6-O-glucuronide-7-O-glucuronide | S | + | + |
| M12 | 3.06 | C_26_H_30_O_15_ | n.d. | n.d. | 581.1481(-4.7) | 405,243 | TSG-glucuronide | PM | + | + |
| P11 | 3.13 | C_32_H_40_O_19_ | n.d. | n.d. | 727.2123(5.1) | 459,441,423 | parishin C | G | + | + |
| M13 | 3.18 | C_27_H_32_N_2_O_10_ | 545.2145(1.8) | 369 | n.d. | n.d. | demethyl-COR-glucuronide | UR | + | + |
| M14 | 3.29 | C_21_H_18_O_14_S | n.d. | n.d. | 525.0330(-0.3) | 445,269 | 5,6,7-trihydroxyflavone-6-O-Sulfuric acid ester-7-O-glucuronide | S | + | - |
| M15 | 3.43 | C_27_H_34_N_2_O_10_ | 547.2306(2.5) | 371,160 | n.d. | n.d. | demethyl-RIN-glucuronide | UR | + | + |
| P12 | 3.52 | C_14_H_21_N_3_O_5_ | 312.1578(6.0) | 154,198 | n.d. | n.d. | leonurine | L | + | + |
| M16 | 3.53 | C_21_H_24_N_2_O_4_ | 369.1771(-5.6) | 351,267 | n.d. | n.d. | demethyl-ICO | UR | + | + |
| M17 | 3.55 | C_28_H_30_O_18_ | 655.1469(-6.2) | 303,273 | 653.1350(-0.6) | 477,301,271 | methyl-hyperoside-glucuronide | GF,T,L | + | + |
| M18 | 3.57 | C_28_H_28_O_18_ | 653.1322(-4.9) | 461,285,270 | 651.1156(-5.1) | 459,283,268 | wogonoside-5-O-hydroxyl-glucuronide | S | + | - |
| M19 | 3.67 | C_27_H_26_O_17_ | 623.1262(2.2) | 447,271 | 621.1096(0.6) | 445,269 | demethyl-wogonoside-8-O-glucuronidation | S | + | - |
| M20 | 3.68 | C_20_H_21_O_10_ | n.d. | n.d. | 421.1151(3.8) | 245,162 | reduction-2,3,5,4＇-tetrahydroxystilbene-2-O-β- glucuronide | PM | + | + |
| M21 | 3.69 | C_21_H_26_N_2_O_4_ | 371.1967(-1.0) | 339,269 | n.d. | n.d. | demethyl-IRN | UR | + | + |
| P13 | 3.92 | C_20_H_22_O_9_ | n.d. | n.d. | 405.1211(6.1) | 162,242 | 2,3,5,4＇-tetrahydroxystilbene-2-O-β- D-glucoside (TSG) | PM | + | - |
| P14 | 3.96 | C_21_H_20_O_12_ | 465.1036(0.6) | 303,273 | 463.0840(-2.8) | 301,271,255 | hyperoside | GF,T,L | + | + |
| P15 | 3.99 | C_26_H_28_O_13_ | 549.1607(-0.1) | 495,465,393 | 547.1473(3.8) | 529,487,457 | 6-*C*-arabinose-8-*C*-glucose-chrysin | S | + | + |
| M22 | 4.37 | C_20_H_22_O_9_ | n.d. | n.d. | 405.1171(-3.70) | 285,243,225 | 2,3,5,4＇-tetrahydroxystilbene-2-O-β- D-glucoside-keto form | PM | + | - |
| P16 | 4.45 | C_26_H_28_O_13_ | 549.1612(0.7) | 233,113 | 547.1457(0.9) | 529,487,457 | 6-*C*-glucose-8-*C*-arabinose-chrysin | S | + | + |
| M23 | 4.59 | C_21_H_26_N_2_O_3_ | 355.2050(7.88) | 293,144 | n.d. | n.d. | reduction-demethyl-GM/HTE | UR | + | + |
| M24 | 5.10 | C_27_H_26_O_17_ | 623.1245(-0.4) | 447,271 | 621.1059(-5.3) | 445,269 | emodin-glucuronide-glucuronide | PM | + | + |
| M25 | 5.17 | C_27_H_28_O_16_ | 609.1425(-5.0) | 433,271 | 607.1308(1.4) | 431,269 | 5,6,7-trihydroxyflavone-6-O-glucose-7-O-glucuronide | S | + | + |
| M26 | 5.60 | C_28_H_28_O_17_ | 637.1409(0.6) | 461,285,270 | 635.1218(-4.7) | 459,283,268 | wogonoside -5-O-glucuronide | S | + | + |
| M27 | 5.82 | C_22_H_22_O_12_ | n.d. | n.d. | 477.1039(1.2) | 301,271 | methyl-hyperoside | GF,T,L | + | + |
| P17 | 6.07 | C_22_H_26_N_2_O_4_ | 383.1949(-5.7) | 351,319,267 | n.d. | n.d. | isocorynoxeine(ICO) | UR | + | + |
| M28 | 6.16 | C_21_H_24_O_9_ | n.d. | n.d. | 419.1323(-4.5) | 243,162 | methyl-TSG | PM | + | + |
| M29 | 6.21 | C_28_H_28_O_18_ | 653.1352(-0.6) | 477,301,286 | 651.1165(-6.4) | 475,299,284 | 5,2´-dihydroxy-6´-methoxy-flavone-7-O-glucuronide- glucuronidation | S | + | + |
| M30 | 6.27 | C_28_H_28_O_18_ | 653.1324(-4.5) | 477,271 | 651.1151(-7.0) | 475,445,269 | hydroxyl-methyl-baicalin glucuronidation | S | + | - |
| M31 | 6.46 | C_22_H_20_O_14_S | n.d. | n.d. | 539.0503(1.30) | 459,283,268 | wogonoside sulfation | S | + | - |
| M32 | 6.60 | C_21_H_24_O_9_ | n.d. | n.d. | 419.1327(-3.5) | 243,162 | methyl -TSG | PM | + | - |
| P18 | 6.75 | C_22_H_28_N_2_O_4_ | 385.2124(-0.7) | 353,321,241 | n.d. | n.d. | isorhynchophylline(IRN) | UR | + | + |
| P19 | 6.86 | C_22_H_26_N_2_O_4_ | 383.1970(-0.3) | 351,319,267 | n.d. | n.d. | corynoxeine(COR) | UR | + | + |
| M33 | 7.03 | C_18_H_16_O_7_ | 345.0971(-0.5) | 327,285 | 343.0839(-5.5) | 313,273,164 | 5,2´-dihydroxy-7,8,6´-trimethoxy flavone | S | + | + |
| P20 | 7.25 | C_21_H_18_O_11_ | 447.0919(-1.8) | 271,353,169 | 445.0768(-0.6) | 269,175,113 | baicalin | S | + | + |
| M34 | 7.34 | C_21_H_24_O_9_ | n.d. | n.d. | 419.1337(-1.19) | 243,162 | methyl -TSG | PM | + | + |
| M35 | 7.78 | C_22_H_20_O_12_ | 477.1055(4.6) | 447,271 | 475.0879(0.4) | 445,269 | hydroxyl-methyl-baicalin | S | + | + |
| M36 | 7.82 | C_21_H_24_N_2_O_3_ | 353.1864(-0.2) | 170,144 | n.d. | n.d. | demethyl-GM | UR | + | + |
| P21 | 7.97 | C_22_H_28_N_2_O_4_ | 385.2178(-6.2) | 353,321,241 | n.d. | n.d. | rhynchophylline(RIN) | UR | + | + |
| M37 | 8.55 | C_21_H_24_N_2_O_3_ | 353.1870(1.4) | 170,144 | n.d. | n.d. | demethyl-hirsuteine | UR | + | + |
| M38 | 8.59 | C_22_H_20_O_11_ | n.d. | n.d. | 459.0944(3.70) | 268,283 | 5,6,7-trihydroxy-6-O-methoxyflavone -7-O-glucuronide | S | + | - |
| P22 | 8.84 | C_21_H_18_O_11_ | 447.0923(-0.8) | 271,177 | 445.0788(3.8) | 269,225,175 | apigenin-7-O-*β*-D-glucuronide | S | + | + |
| P23 | 9.28 | C_21_H_18_O_10_ | 431.0968(-0.8) | 225,153 | 429.0820(-0.7) | 253 | chrysin-7- O-*β*-D-glucuronide | S | + | + |
| P24 | 9.32 | C_22_H_20_O_11_ | 461.1078(-1.3) | 285,270 | 459.0915(-2.6) | 283,268 | oroxylin A-7-O-*β-*D- glucur onide | S | + | + |
| M39 | 9.48 | C_21_H_18_O_11_ | n.d. | n.d. | 445.0766(-1.12) | 269 | emodin 3-O-*β*-D-glucuronide | PM | + | + |
| P25 | 9.58 | C_22_H_20_O_12_ | 477.1040(1.5) | 301,286 | 475.0888(-2.6) | 284,299 | 5,2´-dihydroxy-6´-methoxy-flavone-7-O-glucuronide | S | + | + |
| M40 | 9.83 | C_21_H_18_O_11_ | 447.0925(-0.4) | 271 | 445.0751(-4.4) | 269 | demethy-wogonoside | S | + | + |
| P26 | 10.01 | C_22_H_20_O_11_ | 461.1086(-0.4) | 285,270 | 459.0933(1.3) | 283,268,175 | wogonoside | S | + | + |
| P27 | 10.05 | C_21_H_20_O_10_ | 433.1125(-2.3) | 271,162 | 431.1013(1.3) | 269,225 | emodin-8-O-*β*-D-glucoside | PM | + | + |
| P28 | 10.84 | C_22_H_26_N_2_O_3_ | 367.2024(0.5) | 335,170,122 | n.d. | n.d. | geissoschizine methyl ether(GM) | UR | + | + |
| M41 | 10.98 | C_16_H_12_O_8_S | 365.0355(6.5) | 285,270 | 363.0169(-1.6) | 283,268 | wogonin sulfation | S | + | + |
| M42 | 11.12 | C_16_H_12_O_5_ | 285.0755(-2.8) | 270,240 | 283.0587(-6.7) | 268,238 | oroxylin A | S | + | + |
| P29 | 11.31 | C_22_H_26_N_2_O_3_ | 367.2051(7.9) | 335,170,144 | n.d. | n.d. | hirsuteine | UR | + | - |
| P30 | 11.35 | C_16_H_12_O_6_ | 301.0718(2.0) | 284 | n.d. | n.d. | 5,7,4´-trihydroxy-8-methoxyflavone | S | + | - |
| M43 | 11.37 | C_23_H_26_O_9_ | n.d. | n.d. | 445.1116(-4.2) | 269,225 | methyl-emodin-8-O-*β*-D-glucoside | PM | + | + |
| P31 | 11.38 | C_15_H_10_O_5_ | 271.0629(8.5) | 253,225,171 | 269.0464(5.2) | 251,223,197 | baicalein | S | + | + |
| P32 | 11.66 | C_22_H_28_N_2_O_3_ | 369.2208(8.1) | 337,226,170 | n.d. | n.d. | hirsutine | UR | + | + |
| P33 | 12.86 | C_16_H_12_O_5_ | 285.0745(-6.3) | 270,179 | 283.0603(-1.0) | 268,175,192 | wogonin | S | + | + |
| P34 | 12.97 | C_19_H_18_O_8_ | 375.1060(-5.3) | 360,345.285 | n.d. | n.d. | 5,2´-dihydroxy-6,7,8,6´-tetramethoxy flavone | S | + | - |
| P35 | 14.10 | C_15_H_10_O_5_ | n.d. | n.d. | 269.0454(1.4) | 225,240 | emodin | PM | + | - |

P: absorbed prototype components, M: metabolites, t*_R_*: retention time, G: Gastrodiae Rhizoma, UR: Uncariae Ramulus Cum Uncis, S: Scutellariae Radix, GF: Gardeniae Fructus, E: Eucommia Cortex, T: Taxilli Herba, PM: Polygoni Multiflori Caulis, L: Leonuri herba, n.d., not detected.

Figure 4 Base-peak intensity chromatograms of blank rat/human urine samples identified by UPLC-Q/TOF MS. (A and B) blank rat urine samples in positive and negative mode; (C and D) blank human urine samples in positive and negative mode.

Figure 5 Base-peak intensity chromatograms of blank rat/human urine samples identified by UPLC-Q/TOF MS. (A and B) blank rat urine samples in positive and negative mode; (C and D) blank human urine samples in positive and negative mode.

Figure 6 The extract ion chromatograms of reference standards.


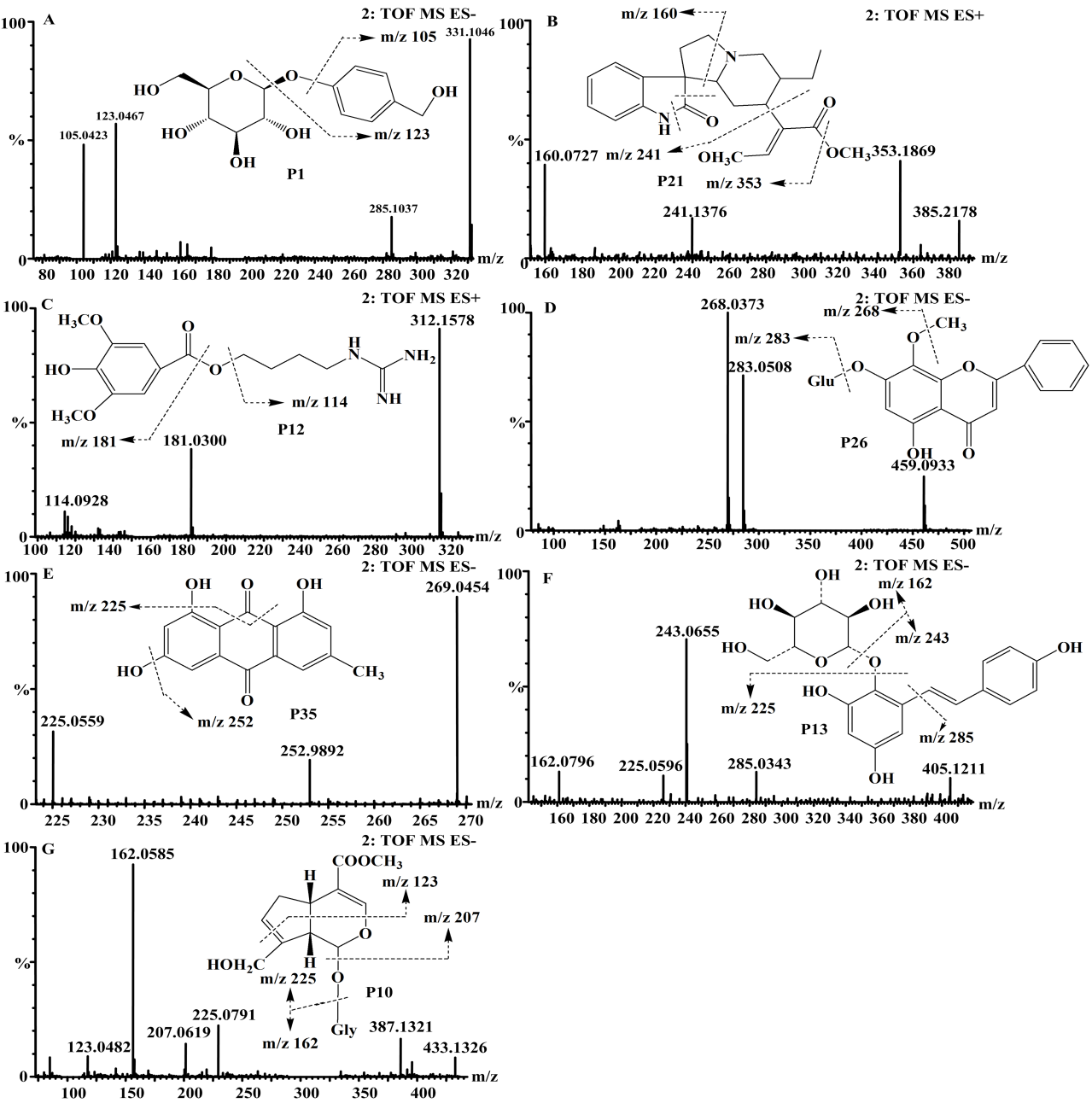


**Figure 7** The product ion spectra and proposed fragmentation pathways of P21 (B), P12(C) in positive ion mode and P1(A) , P26 (D) , P35 (E) , P13 (F) , P10 (G) in negative ion mode.

**A**

**c**

**M14**

**b**

**c**

**M30**

**M25**

baicalein **(P31)**

**M38**

**h**

**M35**

**M11**

**c**

baicalin **(P20)**

**e**

**f**

**B**

e

**a,c**

**c**

wogonin **(P33)**

**M41**

**a**

wogonoside **(P26)**

**e**

**M19**

**M40**

**M26**

**h**

**c**

**M18**

**M31**

**C**

**k**

5,2´-dihydroxy-6,7,8,6´-tetra-

methoxy flavone **(P34)**

**M33**

**D**

5,2´-dihydroxy-6´-methoxy-

flavone-7-O-glucuronide **(P25)**

**M29**

**c**

**E**

hyperoside **(P14)**

**M27**

**b**

**c,h**

**M17**

**F**

**M16**

**a,c**

**isocorynoxeine (P17)**

**M13**

**M3**

**corynoxeine (P19)**

**a**

**c**

**g**

isorhynchophylline **(P18)**

**G**

**a**

**a,c**

**M15**

**M21**

**M6**

**c**

**g**

rhynchophylline **(P21)**

**g**

**H**

**a**

**M23**

**M37**

geissoschizine

methyl ether **(P28)**

hirsuteine **(P29)**

**M36**

**d**

**d**

**a**

or

**I**

**M4**

leonurine **(P12)**

**c**

**J**

geniposide **(P10)**

**M7**

**M10**

**j,c**

**j,e**

**K**

**M1**

shanzhiside **(P2)**

**b**

**M**

gastrodin **(P1)**

**j**

**i**

**M8**

**M5**

**N**

**M2/M9/M12**

**M22**

**c**

**g**

**c,d**

**b**

2,3,5,4＇-tetrahydroxystilbene-2-O-β-D-glucoside **(P13)**

**M28/M32/34**

**M20**

**Figure 8** The proposed metabolic pathways of flavonoid-related metabolites (A–E), alkaloid-related metabolites (F–I), iridoid glycoside-related metabolites (J, K), anthraquinone-related metabolites (L), phenol-related metabolites (M) and stilbene-related metabolites (N) in rat urine. (a. demethylation, b. methylation, c. glucuronidation (Glu), d. reduction, e. sulfation, f. glyconic (Gly), g. isomerization; h.hydroxylation, i. Acetylation, j. deglucoside, k. demethoxylation.).


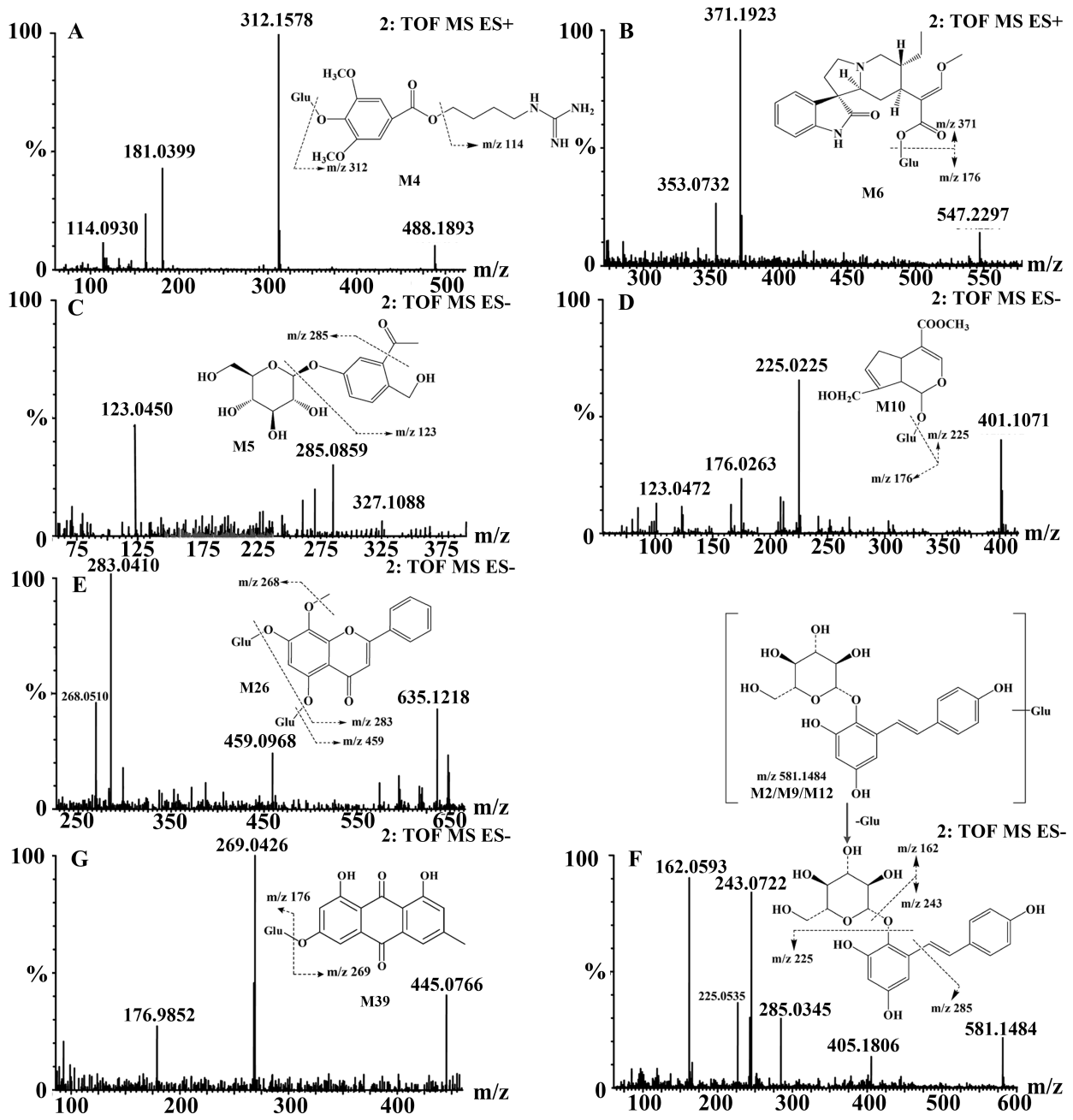


Figure 9 The product ion spectra and proposed fragmentation pathways of M4 (A) , M6 (B) , detected in positive ion mode, and M5 (C) , M1 (D) , M26 (E) , M28/M32/M34 (F) , and M39 (G) detected in negative ion mode.


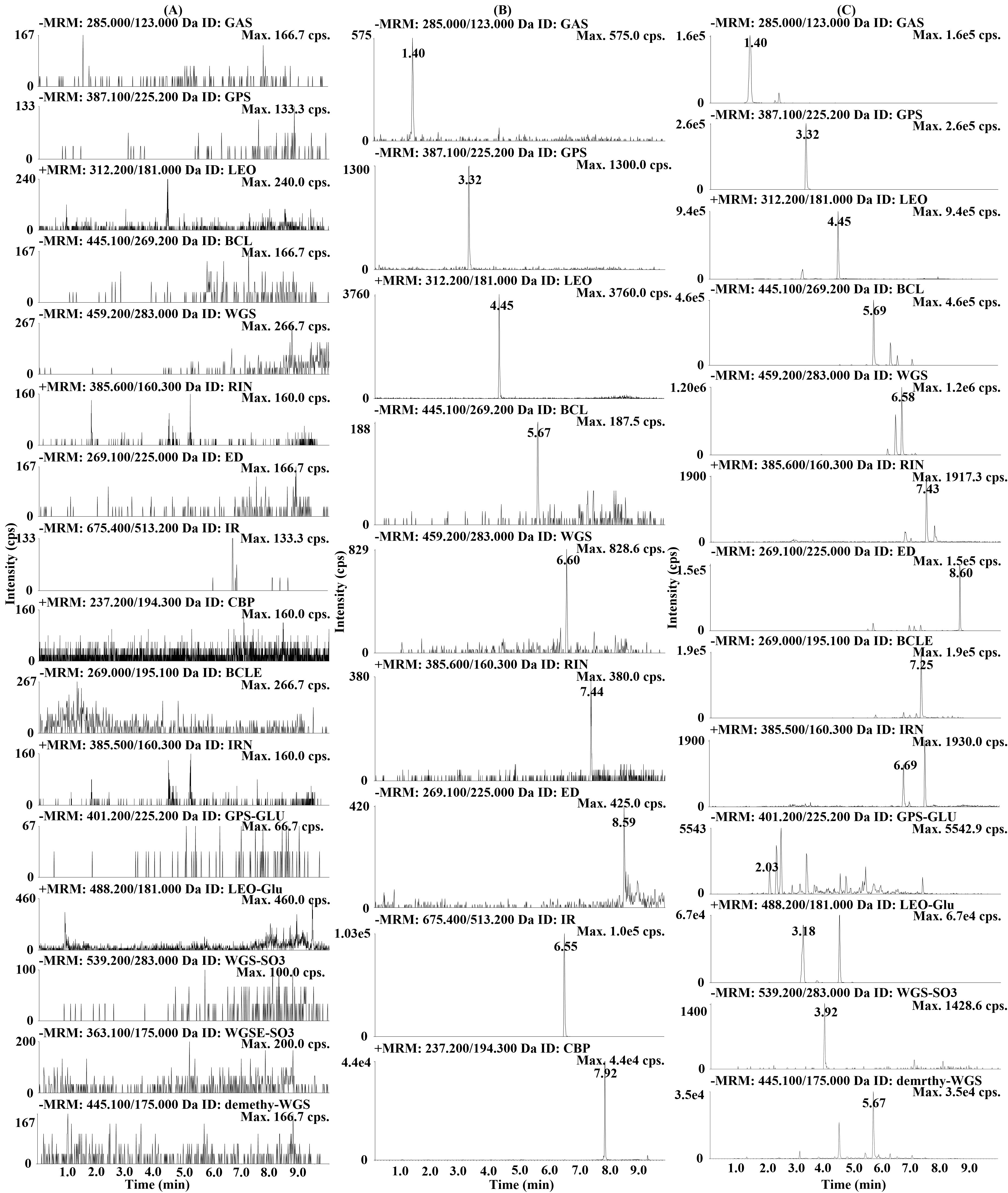


Figure 10 Representative MRM chromatograms of blank rat urine (A), blank rat urine spiked with analytes and IS (B) and rat urine sample after oral administration of TGG with IS (C). CBP and IR are internal standards of positive and negative ion modes, respectively.

1. **Identification of constituents in Qiju Dihuang Pill**

Deng Junjie, Xie Zheyu,He Lina. Simultaneous determination of contents of 11 components in Qiju Dihuang Pills(Condensed) by HPLC-MS/MS[J].Northwest Pharmaceutical Journal,2021,36(05):713-717.

Qiju Dihuang Pill (Zhongjing Wanxi Pharmaceutical Co., Ltd, Lot No. Z41021905, human daily dose at 8 pills/three times).

Take this product 2.0g, add 50mL of methanol with a volume fraction of 70%, heat and reflux for 1h, cool it, weigh the mass again, make up for the lost mass with methanol with a volume fraction of 70%, ultra-high-speed centrifugation, and take the supernatant as the test solution.

Chromatographic column: AgilentPorshellEC-C_18_(50 mm×4.6 mm, 2.7 μm) column, mobile phase: acetonitrile-1 mL·L^-1^. Formic acid (gradient elution, see Table 5), column temperature: 30℃, flow rate: 0.5mL·min^-1^, injection volume: 2μL.

Ion source: electrospray ion source; Dry gas and collision gas: nitrogen (mass fraction≥ 99.999%); dry gas temperature: 300℃; Drying gas velocity: 6L·min^-1^; Scan mode: Simultaneous scanning of positive and negative ions; Capillary voltage: 4000 V (+), 3500 V (-); In order to improve the detection sensitivity, a time-based multiple reaction monitoring (MRM) mode, ionization mode, retention time, constant ionization pair, fragmentation voltage, and collision energy (CE) are used, as shown in Table 6.

Table5 The method of gradient elution

| t/min | Acetonitrile/% | 1ml/L Formic acid/% |
| --- | --- | --- |
| 0-2 | 2 | 98 |
| 2-3 | 2→15 | 98→85 |
| 3-15 | 15→25 | 85→75 |
| 5-16 | 25→90 | 75→10 |
| 16-25 | 90 | 10 |

Table 6 Ionization mode,retention time,quantitative ions, fragment and collision energy of 11 components

| Peak | Component Formula | t/min | Formula | Ionization mode | MS | MS/MS | CE/V |
| --- | --- | --- | --- | --- | --- | --- | --- |
| 1 | Betaine | 1.082 | C_5_H_11_NO_2_ | [M+H]^+^ | 118.1 | 58.2 | 30 |
| 2 | Chlorogenic acid | 5.977 | C_16_H_18_O_9_ | [M-H]^-^ | 353.1 | 191.9 | 15 |
| 3 | Luteolin-7-O-glucoside | 9.770 | C_21_H_20_O_11_ | [M-H]^-^ | 447.1 | 285.0 | 25 |
| 4 | 3,5-di-O-caffeoylquinic acid | 10.838 | C_25_H_24_O_12_ | [M-H]^-^ | 515.2 | 191.1 | 35 |
| 5 | Verbascoside | 9.837 | C_29_H_36_O_15_ | [M-H]^-^ | 623.3 | 461.2 | 30 |
| 6 | Morroniside | 5.929 | C_17_H_26_O_11_ | [M+COOH]^-^ | 451.2 | 243.1 | 15 |
| 7 | Loganin | 6.665 | C_17_H_26_O_10_ | [M+COOH]^-^ | 435.2 | 227.1 | 10 |
| 8 | Paeonol | 17.034 | C_9_H_1_0O_3_ | [M-H]^-^ | 165.1 | 150.1 | 18 |
| 9 | Allantoin | 1.148 | C_4_H_6_N_4_O_3_ | [M-H]^-^ | 157.1 | 97.0 | 10 |
| 10 | Pachymic acid | 19.306 | C_33_H_52_O_5_ | [M+H]^+^ | 529.4 | 451.4 | 15 |
| 11 | 23-Acetatelisol B | 19.424 | C_32_H_50_O_5_ | [M+H]^+^ | 515.4 | 437.3 | 15 |


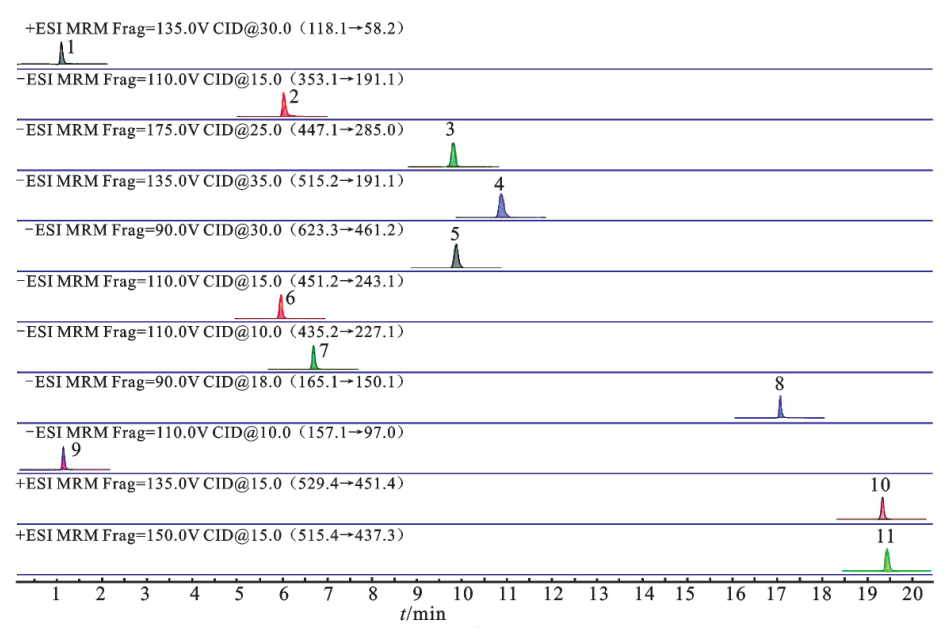


A.


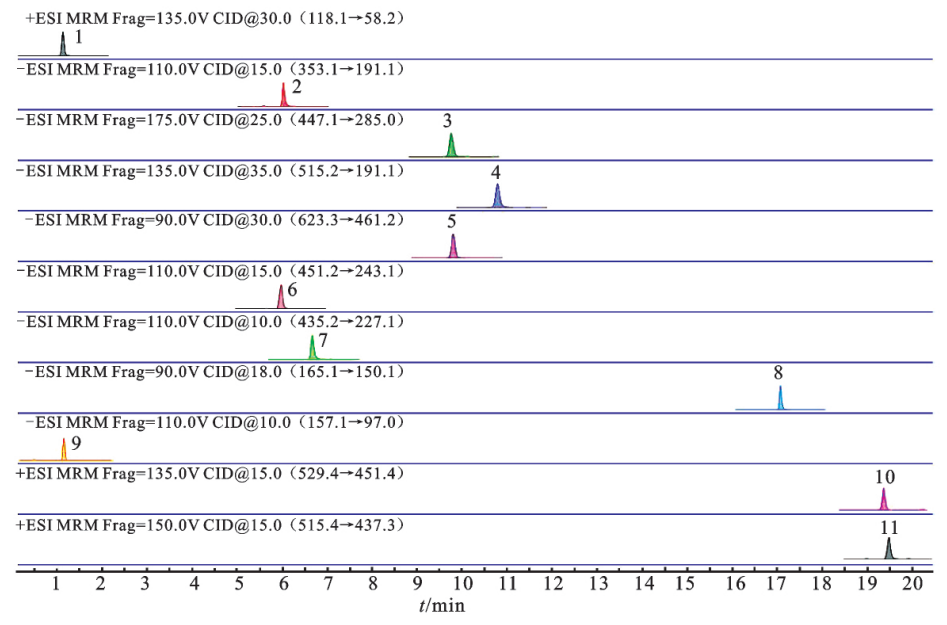


C.

B.


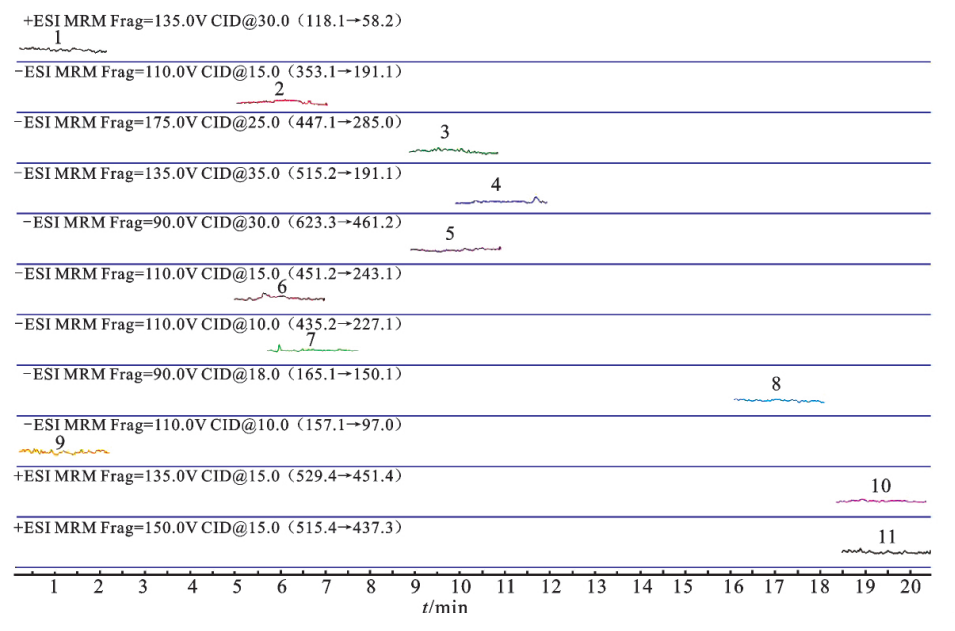


Figure 11 Extracting ions chromatogram of 11 components

Notes:A. Mixed reference material; B. Test solution; C. Control solution

1. Betaine; 2. Chlorogenic acid; 3. Luteolin-7-O-glucoside; 4. 3,5-di-O- caffeoyl- quinic acid; 5. Verbascoside; 6. Morroniside; 7. Loganin;8. Paeonol; 9. Allantoin; 10.Pachymic acid; 11. 23-Acetatelisol B

1. **Identification of constituents in Qinggan Jiangya Capsule**

Jian Juhui,Dai Lingjie,Wang Weidong. Simultaneous determination of 10 components in Qinggan Jiangya capsules by HPLC-QAMS[J].Chinese Journal of Pharmaceutical Analysis,2021,41(03):533-542. DOI:10.16155/j.0254-1793. 2021.03. 20.

Qinggan Jiangya Capsule (Beijing Hongtianli Pharmaceutical Co.,Lot No. Z20093712, huan daily dose at 3 capsules/three times).

Test solution: Take this product 1.0g, add 25mL of 70% ethanol solution, ultrasonically extracted (power 250 W; frequency 40 kHz) for 30 min, cool it, make up for the lost mass with 70% ethanol solution.

Control solution: An appropriate amount of each control product was taken and made into a 70% ethanol solution. The concentrations were 2.034, 1.872, 7.636, 3.528, 0.092, 0.176, 0.108, 0.764, 0.128, 0.212 mg/mL in 70% ethanol. Pipette 2.5 mL of each of the above single control solutions in 70% ethanol solution, dilute to scale in a 50 mL flask to get the components concentration : 101.7, 93.6, 381.8, 176.4, 4.6, 8.8, 176.4, 4.6, 8.8, 5.4, 38.2, 6.4 and 10.6 mg/mL.

Negative sample solution: According to the formula ratio of Qinggan Jiangya Capsule and preparation method to prepare solutions that are missing each traditional Chinese medicine separately. Then the negative sample solution was made according to the above method.

Phenomsil C18 (4.6 mm×250mm, 5 μm) chromatographic column: mobile phase A is acetonitrile, and mobile phase B is 0.1% phosphoric acid aqueous solution, with gradient elution (0~11.0 min, 30.0% A; 11.0~17.0 min, 30.0% A→41.0% A; 17.0~29.0 min, 41.0% A→52.0% A; 29.0~47.0 min, 52.0% A→78.0% A; 47.0~56.0 min, 78.0% A→83.0% A; 56.0~65.0 min, 83.0% A→30.0% A). Detection wavelengths are 320 nm (0~17.0 min, detecting 2, 3, 5, 4'- tetrahydroxydiphenylethylene-2-O-β-D-glucopyranoside) , 250 nm (17.0~29.0 min, detecting 3'-hydroxy puerarin, puerarin, and 3'-methoxy puerarin) [9-10] , 208 nm (29.0~47.0 min, detecting Alisol F, Alisol A, 24-acetyl Alisol A, and 23-acetyl Alisol B) [11-12] and 270 nm (47.0~65.0 min, detecting cryptotanshinone and tanshinone IIA) [13]; flow rate 1.0 mL·min-1; column temperature: 30 ℃; injection volume 10 μL. Absorb solutions 10μL separately into a high-performance liquid chromatograph for determination according to law, and the results are shown in the Figure 12. The results showed that the baseline of the recorded chromatographic peaks was stable, the separation between the chromatographic peaks of the components to be measured and the adjacent chromatographic peaks were >1.5, the number of theoretical plates was≥4500 according to the chromatographic peaks of each component, and the negative samples were not measured for the 10 components in the Qinggan Jiangya Capsule.

Table 7 Relative retention time value

| Peak | Component Formula | t_R_/min | Formula |
| --- | --- | --- | --- |
| 1 | 2，3，5，4′-tetrahydroxystilbene -2-O-β-D-glucoside | 0.61 | C_30_H_30_O_12_ |
| 2 | puerarin | - | C_21_H_20_O_9_ |
| 3 | 3′ -hydroxypuerarin | 0.84 | C_21_H_20_O_10_ |
| 4 | 3′-methoxypuerarin | 1.13 | C_22_H_22_O_10_ |
| 5 | cryptotanshinone | 2.17 | C_19_H_20_O_3_ |
| 6 | tanshinone ⅡA | 2.30 | C_19_H_18_O_3_ |
| 7 | alisol F | 1.45 | C_30_H_48_O_5_ |
| 8 | alisol A | 1.58 | C_30_H_50_O_5_ |
| 9 | alisol A-24-acetate | 1.75 | C_32_H_52_O_6_ |
| 10 | alisol B 23-acetate | 1.95 | C_32_H_50_O_5_ |


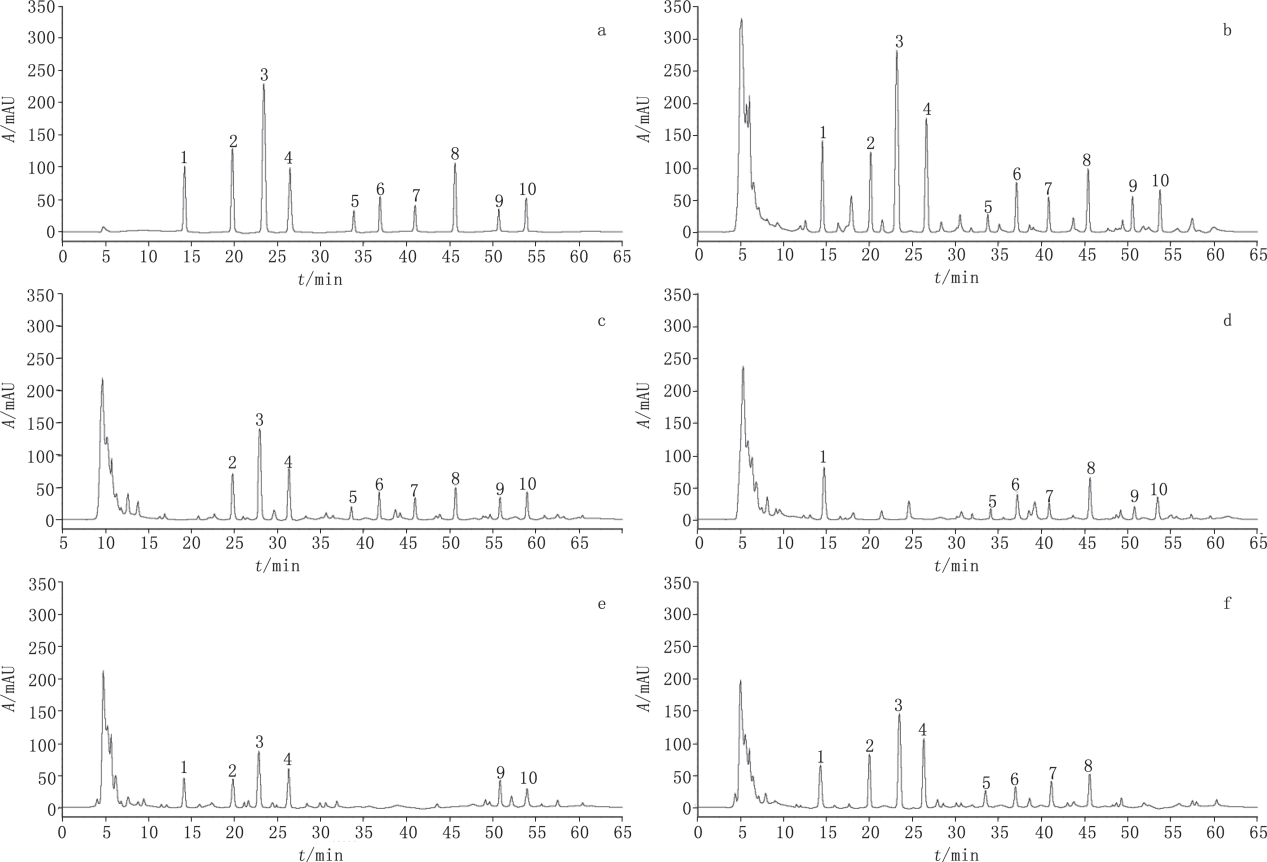


Fig. 12 HPLC chromatograms of mixed reference substances（a），sample（b），negative sample without Polygoni Multiflori Radix Praeparata（c）， negative sample without Puerariae Lobatae Radix and Cyathulae Radix（d），negative sample without Alismatis Rhizoma（e）and negative sample without Salviae Miltiorrhizae Radix Et Rhizoma（f）

*Notes*:1. 2，3，5，4’-tetrahydroxystilbene-2-O-β-D-glucoside; 2. 3’-hydroxypuerarin; 3. puerarin; 4. 3’-methoxypuerarin; 5. alisol F; 6. alisol A; 7. alisol A-24-acetate;

alisol B 23-acetate; 9. cryptotanshinone;10. tanshinone ⅡA

1. **Identification of constituents in Xinmaitong Capsule**

Li Mingyue. HPLC fingerprint and chemical pattern recognition of Xinmaitong capsules[J]. Chinese Journal of Pharmaceutical Analysis,2020,40(06):1104-1112. DOI:10.16155/j.0254-1793.2020.06.20.

Xinmaitong Capsule(Jiangxi Jimin Xinxin Pharmaceutical Co., Ltd, Lot No. Z20153001, human daily dose at 4 capsules/three times; Guizhou yibai pharmaceutical co., LTD, Lot No. Z20060448, human daily dose at 3 capsules/three times).

Test solution: Take 1.0 g of the contents , add 50 mL of 60% methanol water, ultrasound (250 W, 40 kHz) for 30 min, cool them to room temperature, weigh them again and make up for the lost amount with 60% methanol water. Shake and filter through a 0.45 μm microporous membrane.

Agilent SB-C_18_（250 mm× 4.6 mm, 5μm；filler: octadecylsilane bonded silica gel）. Mobile phase: acetonitrile(A)-0.05% phosphoric acid in water (B), gradient elution (0~3 min,5%A； 3~15 min，5%A → 14%A； 15~35 min，14%A →25%A； 35~65 min，25%A → 65%A； 65~70 min，65%A → 5%A）. Flow rate: 1.0 mL·min^-1^. Detection wavelength: 254 nm. Column temperature: 35℃. Injection volume: 20 μL.

After integrating the chromatograms according to the same method, the chromatograms were imported in AIA format into the "Fingerprint Similarity Evaluation System of Traditional Chinese Medicines" (version 2012A). The chromatogram of sample S1 was used as the reference spectrum, and the time window width was set at 0.2. The HPLC fingerprints of 19 batches of samples were superimposed on the control fingerprints (R) in Fig. 13, and a total of 25 peaks were identified. A total of 25 common peaks were identified.


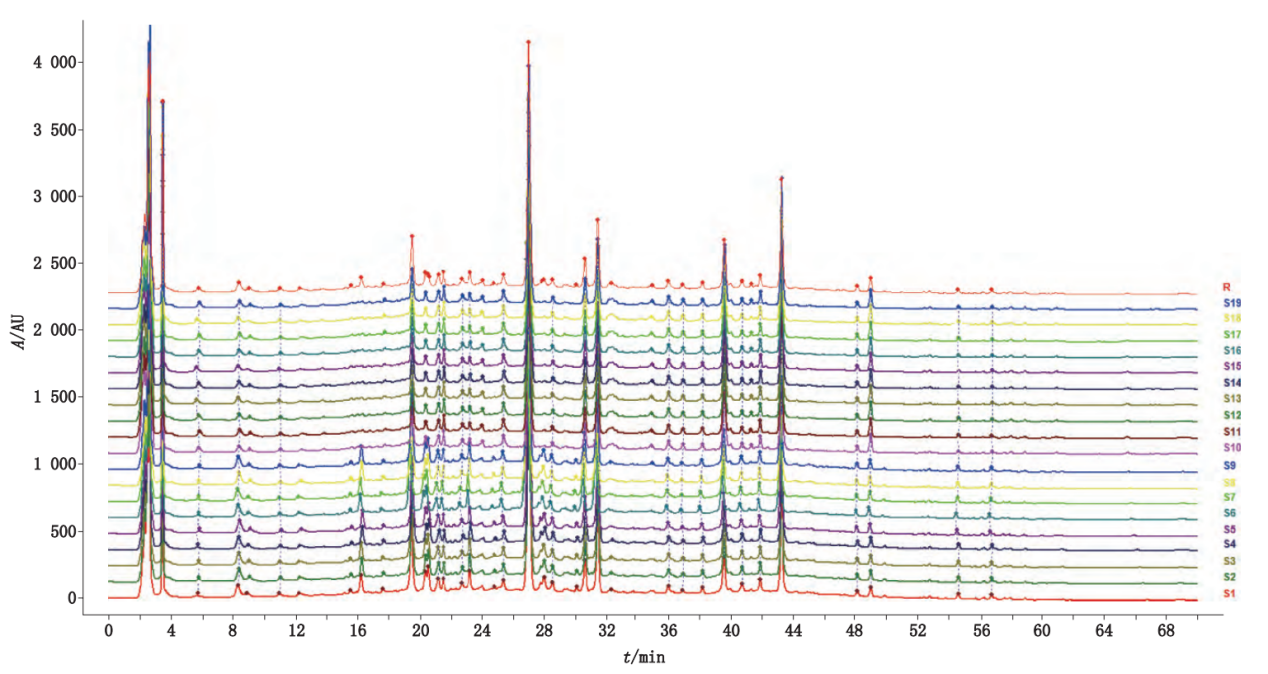


Fig. 13 HPLC fingerprints for 19 batches of Xinmaitong capsules


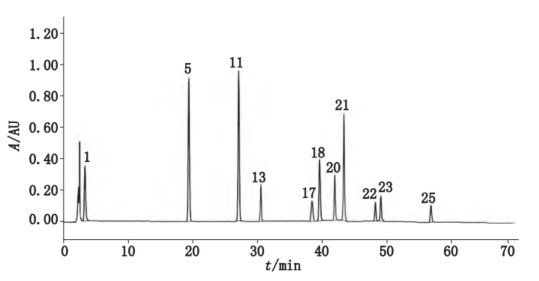


Fig. 14 HPLC chromatogram of mixed reference substances

Notes：1．Rutoside；5．Ligustilide；11. puerarin；13. caffeic acid；17. salvianolic acid B；18. quercetin；20. tanshinone ⅡA；21. notoginsenoside R1；22. ginsenoside Rg1；ginsenoside Rb1；25. aurantio-obtusin


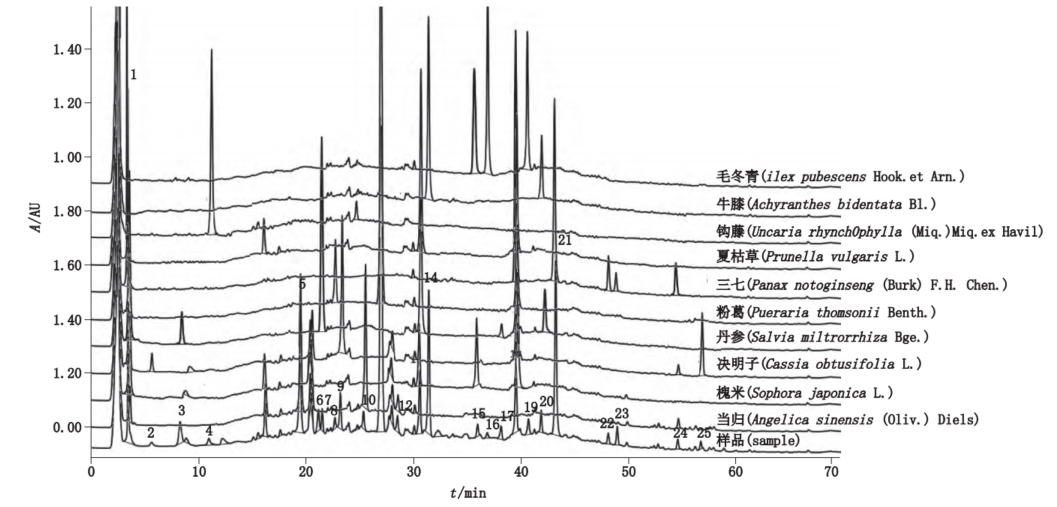


Fig. 15 HPLC chromatograms of Xinmaitong capsules and the single herb medicine
