# Supplementary material for: Chinese patent medicine combined with calcium channel blockers in the treatment of essential hypertension:a Bayes network meta-analysis and systematic review
Source: Front Pharmacol. 2024 Mar 15;15:1321405. doi: 10.3389/fphar.2024.1321405 (PMC10978809; doi:10.3389/fphar.2024.1321405)
Supplement: Supplementary file 6 [file Table6.DOCX]

Supplementary Material

# Supplementary Tables

**Supplementary Table1.** **SUCRA score heat map of outcomes**

| outcome | CCB | QGJY+CCB | QJDH+CCB | QLDX+CCB | SLXM+CCB | TMGT+CCB | XMT+CCB |
| --- | --- | --- | --- | --- | --- | --- | --- |
| SBP | 0.0017 | 0.6854 | 0.3415 | 0.6671 | 0.6323 | 0.8399 | 0.3320 |
| DBP | 0.0181 | 0.6197 | 0.4340 | 0.5510 | 0.8257 | 0.5753 | 0.4762 |
| JY_rate | 0.0003 | 0.8436 | 0.7124 | 0.6455 | 0.4436 | 0.3623 | 0.4923 |
| Syn_score | 0.0422 | - | - | 0.8577 | 0.4855 | 0.4391 | 0.6754 |
| TC | 0.1217 | - | - | - | 0.6183 | 0.8135 | 0.4465 |
| TG | 0.2283 | - | - | - | 0.7127 | 0.4862 | 0.5728 |
| LDL-C | 0.2302 | - | - | - | 0.8709 | 0.3989 | - |
| ad_event | 0.1986 | 0.9924 | - | 0.2930 | 0.3060 | 0.6197 | 0.5904 |
| BPV_SBP | 0.2004 | - | - | - | 0.7856 | 0.5140 | - |
| BPV_DBP | 0.0863 | - | - | - | 0.6588 | 0.7548 | - |
| TNF-α | 0.0506 | - | - | - | 0.9509 | 0.4985 | - |
| IL-6 | 0.1023 | - | - | - | 0.4222 | 0.9755 | - |
| ET-1 | 0.0996 | - | 0.5961 | - | - | 0.8890 | 0.4154 |

*Notes*：SBP：systolic blood pressure；DBP：diastolic blood pressure；JY_rate：antihypertensive effect rate；Syn_score：TCM syndrome score；TC：total cholesterol；TG：triglyceride；LDL-C：low-density lipoprotein cholesterol；ad_event：adverse reactions/events；BPV_SBP：systolic blood pressure variability；BPV_DBP：diastolic blood pressure variability；TNF-α：tumor necrosis factor-α；IL-6：interleukin-6；ET-1：vascular endothelin-1

**2 Supplementary figures**

**2.1 Sensitivity analysis**


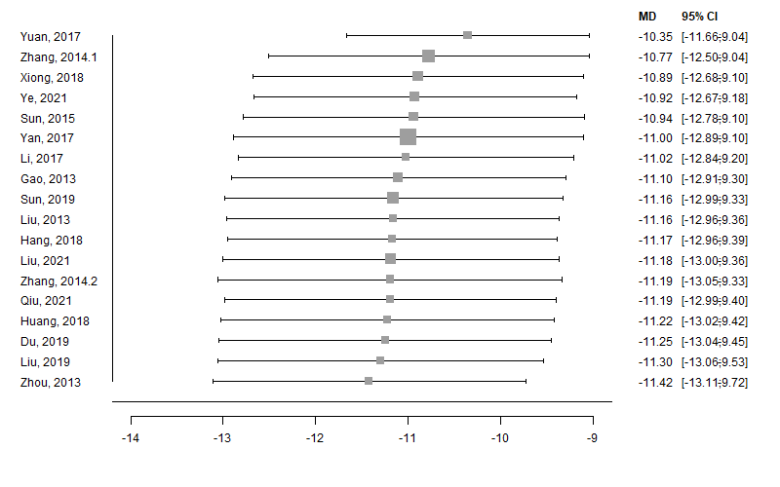


Fig.S1 Sensitivity analysis for SBP


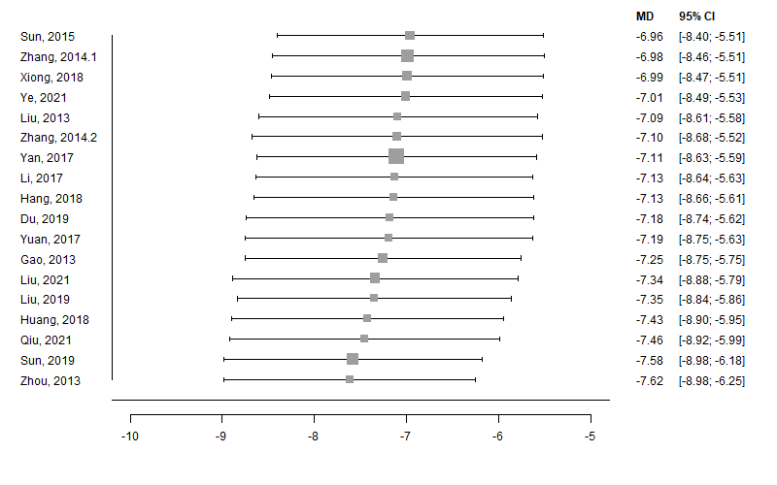


Fig.S2 Sensitivity analysis for DBP


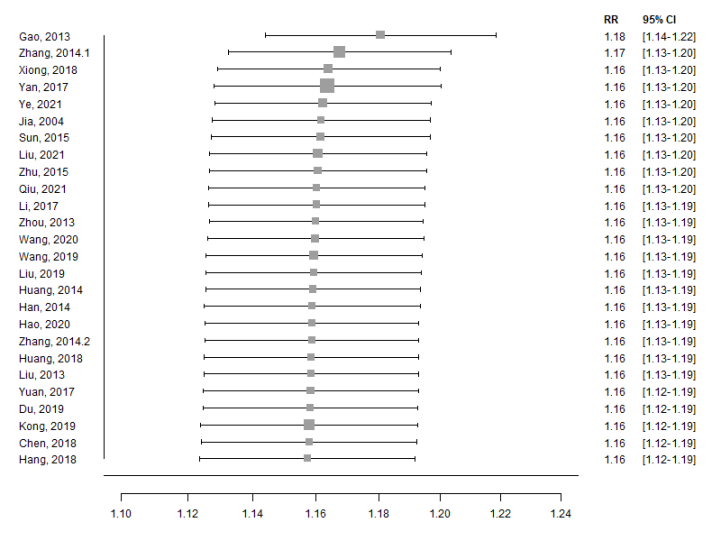


Fig.S3 Sensitivity analysis for antihypertensive rate


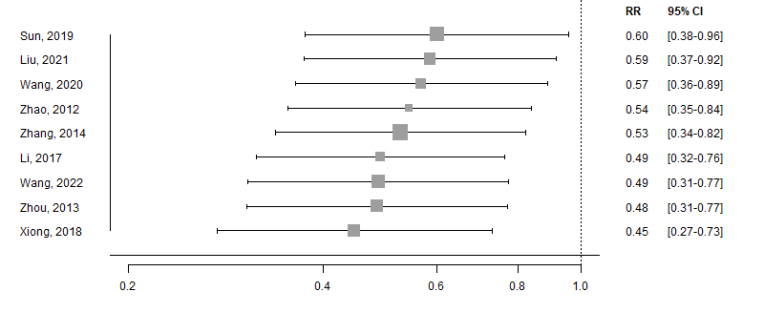


Fig.S4 Sensitivity analysis for adverse reaction/events rate

*Notes*：SBP：systolic blood pressure；DBP：diastolic blood pressure

We planned to perform subgroup analyses by age and interventions, cause these 2 variables might affect the patients’ response to Chinese patent medicines combined with CCB. However, during the data extraction we found that some studies did not report the age of patients so that we only performed subgroup analyses to examine whether there are significant differences in the effectiveness explore which interventions are more effective.

**2.2 Subgroup analysis**


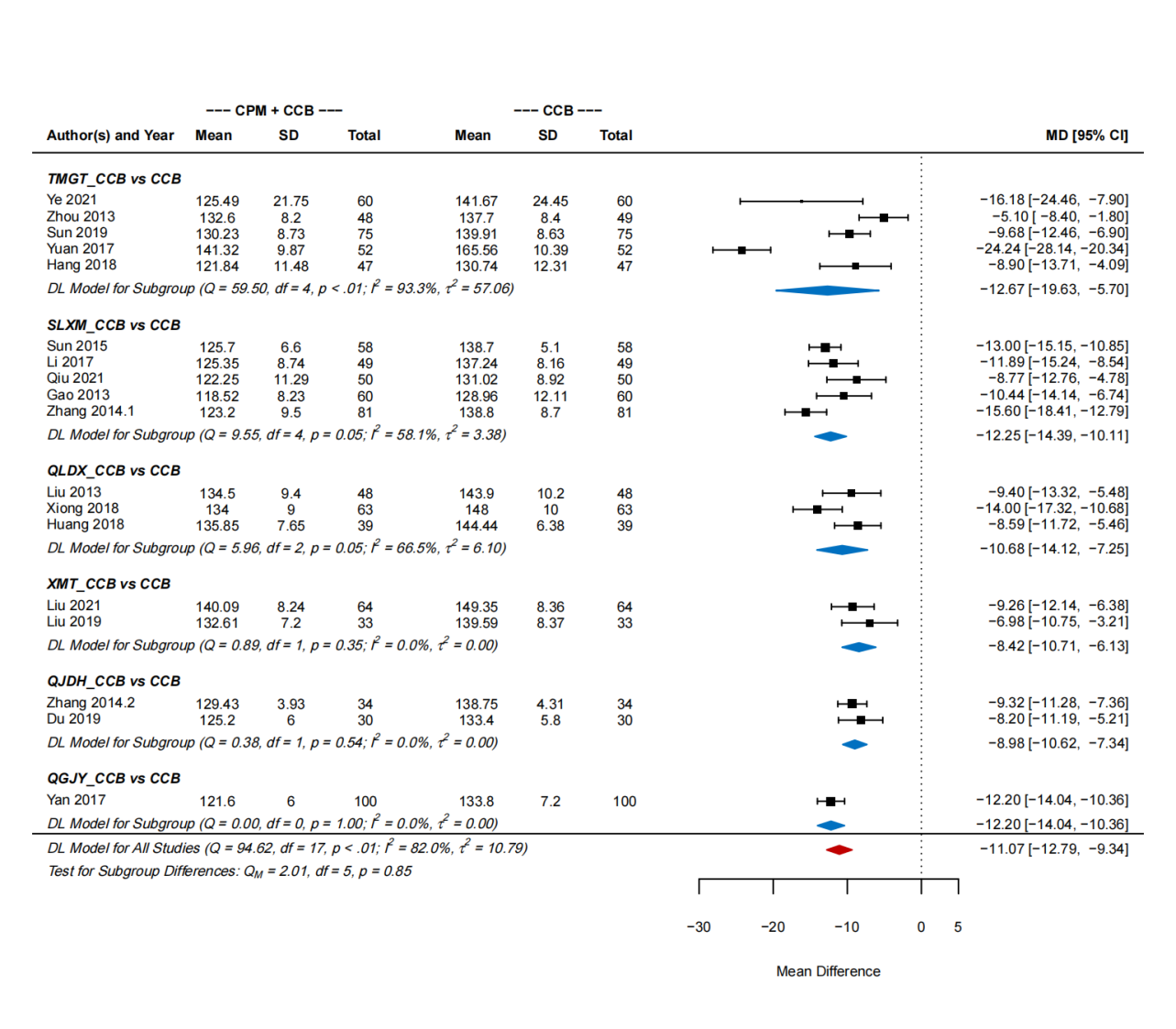


Fig.S5 Subgroup analysis for SBP


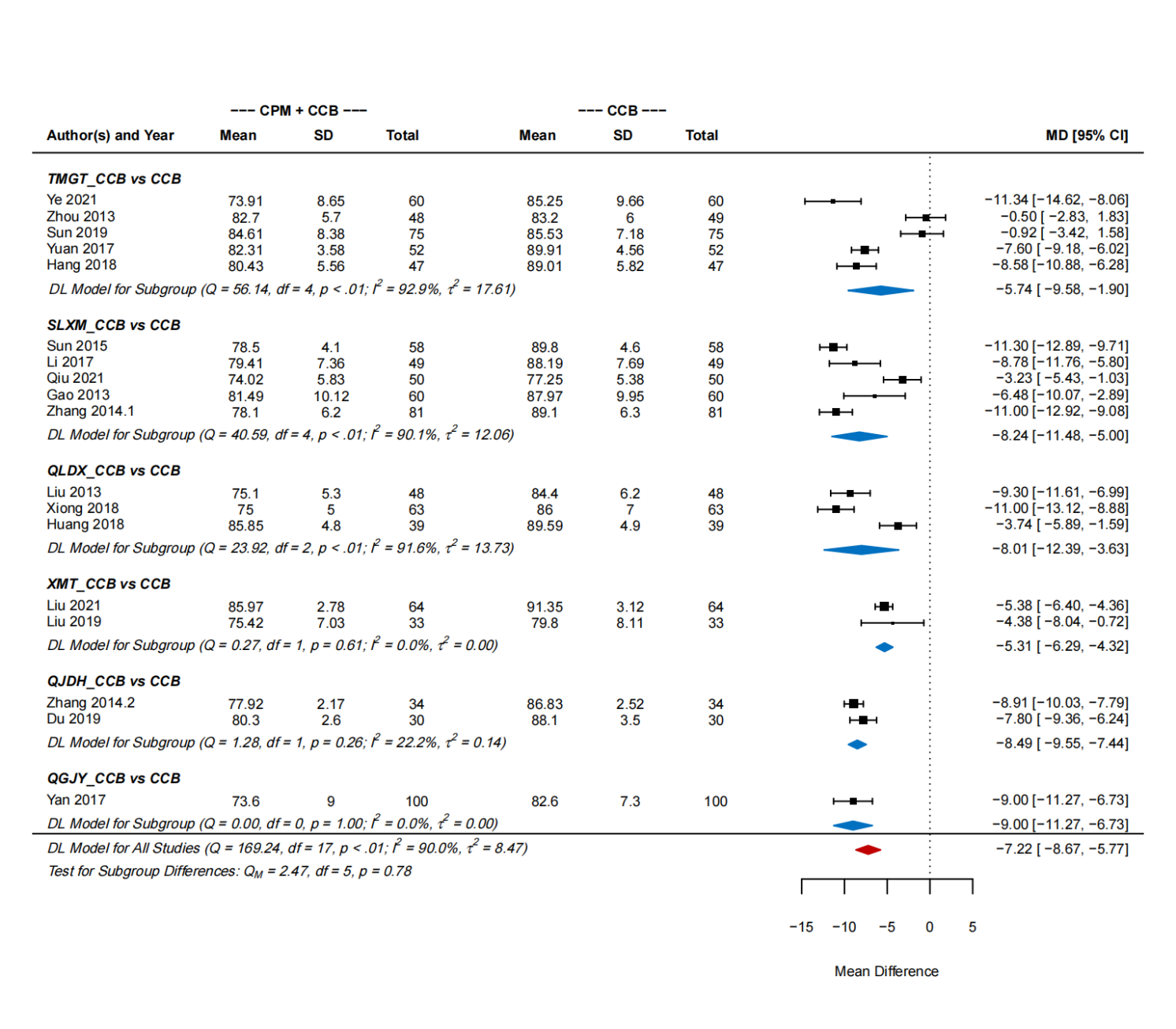


Fig.S6 Subgroup analysis for DBP


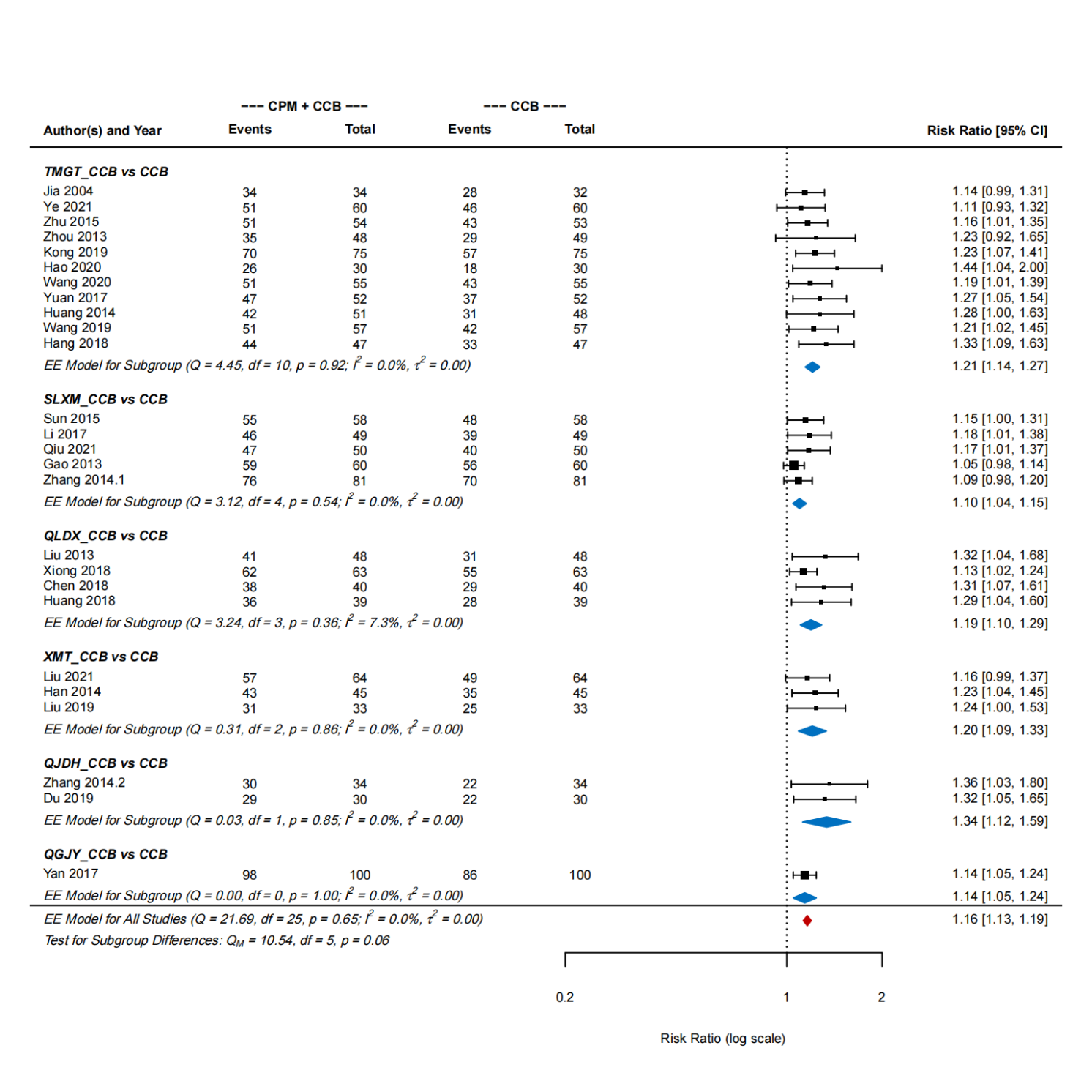


Fig.S7 Subgroup analysis for antihypertensive rate

*Notes*：SBP：systolic blood pressure；DBP：diastolic blood pressure
